# Inclusion of Ethical Issues in Dementia Guidelines: A Thematic Text Analysis

**DOI:** 10.1371/journal.pmed.1001498

**Published:** 2013-08-13

**Authors:** Hannes Knüppel, Marcel Mertz, Martina Schmidhuber, Gerald Neitzke, Daniel Strech

**Affiliations:** 1 Hannover Medical School, Hannover, Germany; 2 University of Mannheim, Mannheim, Germany; 3 University of Salzburg, Salzburg, Austria; University of Cambridge, United Kingdom

## Abstract

**Background:**

Clinical practice guidelines (CPGs) aim to improve professionalism in health care. However, current CPG development manuals fail to address how to include ethical issues in a systematic and transparent manner. The objective of this study was to assess the representation of ethical issues in general CPGs on dementia care.

**Methods and Findings:**

To identify national CPGs on dementia care, five databases of guidelines were searched and national psychiatric associations were contacted in August 2011 and in June 2013. A framework for the assessment of the identified CPGs' ethical content was developed on the basis of a prior systematic review of ethical issues in dementia care. Thematic text analysis and a 4-point rating score were employed to assess how ethical issues were addressed in the identified CPGs. Twelve national CPGs were included. Thirty-one ethical issues in dementia care were identified by the prior systematic review. The proportion of these 31 ethical issues that were explicitly addressed by each CPG ranged from 22% to 77%, with a median of 49.5%. National guidelines differed substantially with respect to (a) which ethical issues were represented, (b) whether ethical recommendations were included, (c) whether justifications or citations were provided to support recommendations, and (d) to what extent the ethical issues were explained.

**Conclusions:**

Ethical issues were inconsistently addressed in national dementia guidelines, with some guidelines including most and some including few ethical issues. Guidelines should address ethical issues and how to deal with them to help the medical profession understand how to approach care of patients with dementia, and for patients, their relatives, and the general public, all of whom might seek information and advice in national guidelines. There is a need for further research to specify how detailed ethical issues and their respective recommendations can and should be addressed in dementia guidelines.

*Please see later in the article for the Editors' Summary*

## Editors’ Summary

**Background:**

In the past, doctors tended to rely on their own experience to choose the best treatment for their patients. Faced with a patient with dementia (a brain disorder that affects short-term memory and the ability tocarry out normal daily activities), for example, a doctor would use his/her own experience to help decide whether the patient should remain at home or would be better cared for in a nursing home. Similarly, the doctor might have to decide whether antipsychotic drugs might be necessary to reduce behavioral or psychological symptoms such as restlessness or shouting. However, over the past two decades, numerous evidence-based clinical practice guidelines (CPGs) have been produced by governmental bodies and medical associations that aim to improve standards of clinical competence and professionalism in health care. During the development of each guideline, experts search the medical literature for the current evidence about the diagnosis and treatment of a disease, evaluate the quality of that evidence, and then make recommendations based on the best evidence available.

**Why Was This Study Done?:**

Currently, CPG development manuals do not address how to include ethical issues in CPGs. A health-care professional is ethical if he/she behaves in accordance with the accepted principles of right and wrong that govern the medical profession. More specifically, medical professionalism is based on a set of binding ethical principles—respect for patient autonomy, beneficence, non-malfeasance (the “do no harm” principle), and justice. In particular, CPG development manuals do not address disease-specific ethical issues (DSEIs), clinical ethical situations that are relevant to the management of a specific disease. So, for example, a DSEI that arises in dementia care is the conflict between the ethical principles of non-malfeasance and patient autonomy (freedom-to-move-at-will). Thus, healthcare professionals may have to decide to physically restrain a patient with dementia to prevent the patient doing harm to him- or herself or to someone else. Given the lack of guidance on how to address ethical issues in CPG development manuals, in this thematic text analysis, the researchers assess the representation of ethical issues in CPGs on general dementia care. Thematic text analysis uses a framework for the assessment of qualitative data (information that is word-based rather than number-based) that involves pinpointing, examining, and recording patterns (themes) among the available data.

**What Did the Researchers Do and Find?:**

The researchers identified 12 national CPGs on dementia care by searching guideline databases and by contacting national psychiatric associations. They developed a framework for the assessment of the ethical content in these CPGs based on a previous systematic review of ethical issues in dementia care. Of the 31 DSEIs included by the researchers in their analysis, the proportion that were explicitly addressed by each CPG ranged from 22% (Switzerland) to 77% (USA); on average the CPGs explicitly addressed half of the DSEIs. Four DSEIs—adequate consideration of advanced directives in decision making, usage of GPS and other monitoring techniques, covert medication, and dealing with suicidal thinking—were not addressed in at least 11 of the CPGs. The inclusion of recommendations on how to deal with DSEIs ranged from 10% of DSEIs covered in the Swiss CPG to 71% covered in the US CPG. Overall, national guidelines differed substantially with respect to which ethical issues were included, whether ethical recommendations were included, whether justifications or citations were provided to support recommendations, and to what extent the ethical issues were clearly explained.

**What Do These Findings Mean?:**

These findings show that national CPGs on dementia care already address clinical ethical issues but that the extent to which the spectrum of DSEIs is considered varies widely within and between CPGs. They also indicate that recommendations on how to deal with DSEIs often lack the evidence that health-care professionals use to justify their clinical decisions. The researchers suggest that this situation can and should be improved, although more research is needed to determine how ethical issues and recommendations should be addressed in dementia guidelines. A more systematic and transparent inclusion of DSEIs in CPGs for dementia (and for other conditions) would further support the concept of medical professionalism as a core element of CPGs, note the researchers, but is also important for patients and their relatives who might turn to national CPGs for information and guidance at a stressful time of life.

**Additional Information:**

Please access these Web sites via the online version of this summary at http://dx.doi.org/10.1371/journal.pmed.1001498.

- Wikipedia contains a page on clinical practice guidelines (note: Wikipedia is a free online encyclopedia that anyone can edit; available in several languages)
- The US National Guideline Clearinghouse provides information on national guidelines, including CPGs for dementia
- The Guidelines International Network promotes the systematic development and application of clinical practice guidelines
- The American Medical Association provides information about medical ethics; the British Medical Association provides information on all aspects of ethics and includes an essential tool kit that introduces common ethical problems and practical ways to deal with them
- The UK National Health Service Choices website provides information about dementia, including a personal story about dealing with dementia
- MedlinePlus provides links to additional resources about dementia and about Alzheimers disease, a specific type of dementia (in English and Spanish)
- The UK Nuffield Council on Bioethics provides the report Dementia: ethical issues and additional information on the public consultation on ethical issues in dementia care

## Introduction

Clinical practice guidelines (CPGs) are meant to improve standards of clinical competence and professionalism by referring explicitly to evidence-based information on benefits and harms [1]. Their development increasingly includes measures to strengthen their validity and accountability: patient participation [2], explicit procedures to grade the strength of recommendations [3], and the requirement to disclose and manage conflicts of interest [4]. While all these developments in guideline methodology are laudable, particularly from an ethical perspective, CPG development manuals worldwide still fail to address how to include disease-specific ethical issues (DSEIs): a search of leading CPG development manuals for the term “ethics” or “ethical” does not yield any information about how to identify and address clinical ethical situations that are relevant to the management of specific diseases [1],[5]–[8]. What has been addressed in some manuals is the need to realize that (a) different groups value outcomes differently and (b) values and preferences should be considered when making guideline recommendations.

How does a DSEI arise? Widely shared frameworks for medical professionalism and common approaches to morality in bioethics are all based on a set of prima facie binding ethical principles: respect for patient autonomy, beneficence, non-maleficence, and justice [9]–[11]. Once we accept that these ethical principles are relevant to health-related decision-making, a DSEI can arise from (a) neglect of one or more ethical principles, for example, “Insufficient consideration of patient autonomy and patient preferences in dementia care decisions”, or (b) conflicts between two or more ethical principles, for example, “Balancing the do-no-harm principle (non-maleficence) versus the freedom-to-move-at-will principle (patient autonomy) in decision-making for or against physical restraints on account of inappropriate patient behaviour.”

Awareness not only of the four general ethical principles but especially of relevant DSEIs, and competency in managing these DSEIs, are deeply intertwined with the concepts of clinical competence and professionalism of health care workers [9]. Furthermore, awareness of and competence with respect to DSEIs is important also for other caregivers, such as relatives of persons with dementia [12].

Although no current CPG development manual explains how to identify and integrate specific DSEIs, it is unclear whether and to what extent existing CPGs already address DSEIs. While some research has been conducted on the prevalence, content, and quality of ethical guidelines [13]–[15], to our knowledge no study has yet investigated in a systematic manner how CPGs vary in addressing DSEIs. Because ethical issues pervade dementia care, and much has been written on this topic [16]–[18], existing dementia care guidelines provide a good starting point to look more specifically at whether and how DSEIs are addressed.

The objective of this study was to assess the representation of DSEIs in national evidence-based CPGs on dementia care.

## Methods

### Identification of Clinical Practice Guidelines on Dementia Care

To maintain comparability, we restricted our analysis to national, general CPGs on dementia care and therefore did not analyze guidelines that address a specific aspect of dementia care. Further reasons to restrict our analysis to national guidelines addressing the whole spectrum of dementia care were the following: First, national CPGs (from institutions such as National Institute for Health and Clinical Excellence [NICE] in the United Kingdom or the American Psychiatric Association [APA] in the United States) must meet high standards for guideline development. Second, we believe that national CPGs should be most interested in addressing DSEIs as they often also address patients and relatives as potential readership.

We restricted our search to national guidelines written in English or German. To identify national CPGs we searched the following five guideline databases: (1) National Guideline Clearinghouse (United States), (www.guideline.gov), (2) National Library for Health on Guidelines Finder (United Kingdom), (www.library.nhs.uk/GuidelinesFinder/), (3) Canadian Medical Association Infobase (Canada), (www.cma.ca/index.php/ci_id/54316/la_id/1.htm), (4) G-I-N International Guideline Library (www.g-i-n.net), (5) AWMF (Germany) (www.awmf.org/leitlinien/leitlinien-suche.html). The date of the search was August 15, 2011. We complemented the search by contacting national psychiatric associations and societies in Switzerland and Austria, because no guidelines could be identified for these two German-speaking countries in the above-mentioned databases. We repeated the guideline search on June 10, 2013, but found neither additional national guidelines nor changes in the already-retrieved guidelines. Because a revised version of the Canadian dementia guideline (from 2012) currently undergoes a public consultation process this study assessed the still official guideline from 2007.

### Development of a Matrix of Ethical Issues in Dementia Care

Prior to this guideline assessment study we conducted a systematic review in Medline (restricted to English and German literature published between 2001 and 2011) and Google Books (restricted to the first 100 hits) to identify the full spectrum of ethical issues in dementia care. More detailed methodological information and the findings of this systematic review are published elsewhere [19]. In total, this systematic review retrieved 92 references that together mentioned a spectrum of 56 DSEIs in dementia care. The DSEIs were grouped under 33 mid-level categories that were themselves grouped under seven main categories.

For the purpose of this study the 56 DSEIs were reduced to 31 broader DSEIs (grouped under the same seven main categories). In most cases we broadened the content of a DSEI by referring to the original mid-level categories, e.g., the narrow DSEIs, (a) “Insufficient consideration of the patient as a person” and (b) “Insufficient consideration of existing preferences of the patient” were reduced to the broader DSEI “Adequate appreciation of the patient.” We further reduced the number of DSEIs where mid-level categories were sufficiently related (e.g., we synthesized the DSEIs “Adequate amount and manner of information” and “Consideration of cultural aspects” into the broader DSEI “Adequate consideration of the complexity of informing patients with dementia”). While the wording of some DSEIs is generic (e.g., “Responsible handling of costs and allocation of limited resources”), the corresponding text examples always highlight specific challenges related to dementia care. Text examples for all DSEIs have been published elsewhere [19].

This spectrum of 31 DSEIs was used as a framework for the assessment of ethical content in each CPG on dementia care.

The concept of DSEIs is rooted in the ethical theory of principlism [10] that forms the basis of many ethical and medical professionalism frameworks [9],[11] (see also the Introduction). Broadly drawn, the term covers all clinical (diagnostic or therapeutic) decisions that need to balance potential benefits and harms. In this regard almost all disease-specific clinical issues automatically become disease-specific ethical issues. We addressed this (unavoidable) challenge of “over-inclusiveness” by only considering a dementia-specific clinical issue to be a DSEI when we identified examples from the literature indicating an important controversy on how to balance benefits and harms. For example, the clinical decision on whether or not antipsychotic drugs should be used in dementia care was included as an example for the DSEI “Medication” because we found several text passages highlighting specific controversies and practice variations on how to balance benefits and harms regarding this clinical issue.

### Assessment of the Representation of Ethical Issues in Clinical Practice Guidelines on Dementia Care

The representation of all 31 DSEIs in each identified CPG was assessed according to standards in thematic text analysis [20]. All researchers were experienced in thematic text analysis and medical ethics. The academic background of the five researchers included training (degrees) and at least two years' practice in clinical psychiatry (DS), internal medicine (GN), philosophy (DS, MM, MS), public health (HK), social sciences (MM), and physiotherapy (HK). First, CPGs were read in full by at least two researchers independently to identify and extract text passages corresponding to one of the 31 DSEIs in dementia care. Secondly, for every individual CPG the same researchers independently assigned one of the following four possible ratings to each of the 31 DSEI: N, DSEI not addressed; I, DSEI implicitly addressed; E, DSEI explicitly addressed; and R, DSEI explicitly addressed with recommendations. For rating examples see Table 1.

**Table 1:** Text examples illustrating the ratings presented in Tables 3 and 4.

Rating Category	DSEI	Text Examples
N (not addressed)		n.a.
I (implicitly addressed)	“Thorough decision making on the indication for brain imaging (e.g., dealing with current lack of evidence that proves clinical validity of brain imaging in diagnosing dementia)”	“The use of a structural neuroimaging study, such as computerized tomography or magnetic resonance imaging (MRI) scan, is generally recommended as part of an initial evaluation, although clinical practice varies” (p. 16) APA [35]
E (explicitly addressed without recommendation)	“Adequate consideration of the complexity of diagnosing dementia (e.g., unclear cut off for MCI/mild cognitive impairment)”	“The development of dementia pathology occurs many years before the symptoms become obvious. Of interest is the transitional stage of cognitive impairment between normal aging and early AD, the state of mild cognitive impairment (MCI)” (p. 22) MOH Malay [30]
R (explicitly addressed with Recommendation)	“Adequate consideration of the complexity of informing patients with dementia (e.g., the amount and manner of information)”	“The patient and his or her family should be informed of the findings and their meaning by the physician. The setting of this conversation should be appropriate to the personal situation of the patient and the relatives. The nature and content of the education should be oriented to the individual's informational needs and wishes, as well as to the clinical needs of the patient” [translated by DS] (p. 24) DGPPN [29]

n.a., not applicable.

CPGs in category R were further assessed for whether the recommendation was justified in the text or not and whether citations supporting the recommendation were given or not.

After the independent text extraction and rating the researchers compared their results. Discrepancies between the resulting spreadsheets were identified in 51 (13.7%) of 372 ratings. These discrepancies were discussed and resolved by including at least one other researcher with training in both clinical medicine and medical ethics (DS, GN). The latter researchers also supported the validity check for another 32 ratings where both independent reviewers rated equally but still felt some uncertainty concerning the validity of their rating. For text examples from CPGs and their respective ratings, see the Findings section and Table S1.

## Results

In total we included 12 national CPGs from 12 countries in four continents (Table 2).

**Table 2:** Characteristics of included clinical practice guidelines.

Country	Association/Institution	Guideline Title	Publication	Pages
Australia [23]	Royal Australian College of General Practitioners (RACGP)	Care of Patients with Dementia in General Practice	2003	88
Austria [24]	Competence Center Integrated Care	Medizinische Leitlinie für die integrierte Versorgung Demenzerkrankter	2008	198
Canada [25]	Canadian Medical Association (CMA)	3rd Canadian Consensus Conference on Diagnosis and Treatment of Dementia	2007	29
United Kingdom [26],[27]	National Institute for Health and Clinical Excellence (NICE)	Dementia - Supporting people with dementia and their carers in health and social care	2006/2010	392+31
France [28]	Haute Autorité de Santé (HAS)	Alzheimer Disease and Related Conditions: Diagnosis and Treatment^a^	2008	27
Germany [29]	German Association for Psychiatry and Psychotherapy (DGPPN)/German Society of Neurology (DGN)	S3-Leitlinie “Demenzen”	2009	108
Malaysia [30]	Ministry of Health	Management of Dementia	2009	162
New Zealand [31]	New Zealand Guidelines Group (NZGG)	Guidelines for the Support and Management of People with Dementia	1998	47
Scotland [32]	Scottish Intercollegiate Guidelines Network (SIGN)	Management of Patients with Dementia A National Clinical Guideline	2006	57
Singapore [33]	Ministry of Health	Clinical Practice Guideline: Dementia	2007	91
Switzerland [34]	Expert panel Switzerland,	Konsensus zur Diagnostik und Betreuung von Demenzkranken in der Schweiz	2008	31
United States [35]	APA	Practice Guideline for the Treatment of Patients With Alzheimer Disease and Other Dementias	2007	86

a The French language guideline “Diagnostic et prise en charge de la maladie d'Alzheimer et des maladies apparentées” consists of 40 pages but contains the same ten chapters as the English language version.

Six CPGs are published or certificated by a central governmental institution (Australia, France, Malaysia, New Zealand, Singapore, United Kingdom), four by a medical association (Canada, Germany, Scotland, United States), one by a statutory health insurance body (Austria), and one by an expert panel (Switzerland). All guidelines explicitly acknowledged the involvement of experts from different specialties (most often from psychiatry, neurology, gerontology, and family medicine). Only one guideline (from New Zealand) did not describe explicitly which specialties were involved.

All assessed guidelines on dementia care already address clinical ethical issues to some extent. However, CPGs differed considerably as to the number of DSEIs addressed, implicitly or explicitly. The rate of DSEIs that were explicitly addressed by each CPG (calculated as the sum of E and R ratings for each guideline) ranged from 20% (Switzerland) to 77% (United States) with a median of 49.5% (Table 3).

**Table 3:** Quantitative representation of the 31 DSEIs.

Clinical Practice Guideline	Ratings for the Representation of DSEIs (*n* = 31)							
	N		I		E		R	
	*n* (%)		*n* (%)		*n* (%)		*n* (%)	
Australia (RACGP)	11	(35)	4	(13)	10	(32)	6	(19)
Austria (CCIV)	10	(32)	7	(23)	2	(6)	12	(39)
Canada (CMA)	13	(42)	3	(10)	5	(16)	10	(32)
United Kingdom (NICE)	3	(10)	8	(26)	4	(13)	16	(52)
France (HAS)	11	(35)	7	(23)	7	(23)	6	(19)
Germany (DGPPN/DGN)	8	(26)	6	(19)	6	(19)	11	(35)
Malaysia (MOH Malay.)	8	(26)	2	(6)	4	(13)	17	(55)
New Zealand (NZGG)	10	(32)	6	(19)	5	(16)	10	(32)
Scotland (SIGN)	20	(65)	4	(13)	2	(6)	5	(16)
Singapore (MOH Sing.)	8	(26)	2	(6)	4	(13)	17	(55)
Switzerland	15	(48)	10	(32)	3	(10)	3	(10)
United States (APA)	5	(16)	2	(6)	2	(6)	22	(71)

Rating codes: N, DSEI not addressed; I, DSEI implicitly addressed; E, DSEI explicitly addressed; R, DSEI explicitly addressed with recommendations.

When adding all implicitly addressed DSEIs (I, E or R ratings) the rate of DSEIs ranged from 35% (Scotland) to 91% (United Kingdom) with a median of 67.5%. However, the inclusion of recommendations (R rating) on how to deal with the 31 DSEIs (per CPG) ranged from 10% to 71% with a median of 32% (table 3).

Four DSEIs (13%) were neither implicitly nor explicitly addressed in at least 11 out of 12 CPGs: “Adequate consideration of existing advance directives in medical decision making,” “Usage of GPS and other monitoring techniques,” “Covert medication,” and “Dealing with suicidality” (Table 4).

**Table 4:** Representation of 31 DSEIs in 12 national clinical practice guidelines on dementia care.

Ethical Issues in Dementia Care	Country-Specific Ratings											
Diagnosis and medical indication	Australia	Austria	Canada	United Kingdom	France	Germany	Malaysia	New Zealand	Scotland	Singapore	Switzerland	United States
Adequate consideration of the complexity of diagnosing dementia (e.g., unclear cut off for MCI/mild cognitive impairment)	E	R +/+	R +/−	R +/+	I	R −/−	E	I	R +/−	I	I	R +/−
Adequate timing of diagnosis (e.g., guiding an appropriate “breaking bad news” process)	I	E	R −/−	R +/+	I	E	R +/+	I	R +/−	E	I	R +/−
Reasonableness of treatment indications (e.g., risk of overestimating benefits of pharmaceutical treatment)	E	I	R −/−	I	E	R +/−	R +/−	R +/+	I	R +/+	E	R +/−
Adequate appreciation of the patient (e.g., adequate consideration of the patient as a person; adequate consideration of existing preferences of the patient)	E	R +/+	E	R +/−	I	I	R +/+	R +/−	I	R +/+	I	R +/−

Assessing patient decision-making capacity	Australia	Austria	Canada	United Kingdom	France	Germany	Malaysia	New Zealand	Scotland	Singapore	Switzerland	United States
Dealing with varying concepts/understanding of “patient's capacity” among carers	E	N	E	I	I	I	E	E	N	E	N	E
Adequate consideration of setting or content in assessing decision-making capacity (e.g., guidance on how to structure the setting for the capacity assessment; involvement of relatives)	E	I	I	I	I	E	R +/+	N	E	R +/+	I	R −/−
Adequate assessment of patient's decision-making capacity (e.g., inadequate or biased assessment)	E	R +/+	N	E	N	E	R +/+	E	N	R +/+	I	R +/+

Information and disclosure	Australia	Austria	Canada	United Kingdom	France	Germany	Malaysia	New Zealand	Scotland	Singapore	Switzerland	United States
Adequate consideration of the complexity of informing patients with dementia (e.g., the amount and manner of provision of information)	R +/+	R +/+	E	R +/+	R −/+	R +/−	E	R +/−	R +/+	R +/+	N	R +/+
Adequate involvement of relatives in the care process	E	R −/+	R +/−	R +/+	R −/−	R −/−	R +/−	R +/−	R −/+	R +/−	I	R −/−

Decision making and consent	Australia	Austria	Canada	United Kingdom	France	Germany	Malaysia	New Zealand	Scotland	Singapore	Switzerland	United States
Support of patient's decision-making capacity	E	R +/+	I	E	I	R +/−	R −/−	I	I	R +/+	I	R +/+
Responsible surrogate decision making (e.g., interpretation of advance directives from the perspective of the patient; seeking consent among different surrogates prior to making decisions)	I	R +/−	R −/+	I	N	I	I	I	E	R −/−	N	R −/−
Dealing with the need for advance care planning (e.g., sensibly informing patients and relatives about the types of decision that might need to be made; informing them of tools such as advance directives)	R −/−	R +/−	R −/+	R −/+	E	I	R −/+	R +/−	N	N	R +/−	R +/+
Adequate consideration of existing advance directives in medical decision making (e.g., guidance on how to include advance directives in medical decision making; recommendations on how to deal with conflicts between content of advance directives and present behavior and expressions in patients with severe dementia).	N	N	N	N	N	N	N	N	N	N	N	N

Social and context-dependent aspects	Australia	Austria	Canada	United Kingdom	France	Germany	Malaysia	New Zealand	Scotland	Singapore	Switzerland	United States
Caring for relatives (e.g., recognizing needs of relatives; preventing distress and burnout)	R +/−	R +/−	R −/+	R +/−	R +/−	R +/+	R +/+	R +/+	N	R +/+	R +/−	R +/+
Caring for clinical personnel and professional carers (e.g., needs of professionals; preventing distress and burnout)	N	I	N	I	R +/−	I	R +/+	R +/+	N	I	R +/−	N
Adequate assessment of the patient's potential to harm a third person (e.g., in decisions on the ability to drive)	R +/+	E	E	R +/−	I	R +/−	N	R +/−	N	R +/+	E	R +/+
Responsible handling of costs and allocation of limited resources (e.g., avoiding futile diagnostic and treatment)	N	I	N	I	N	I	N	N	N	R +/+	I	I

Care process and process evaluation	Australia	Austria	Canada	United Kingdom	France	Germany	Malaysia	New Zealand	Scotland	Singapore	Switzerland	United States
Continuing assessment of potential benefits and harms in individual care strategies	I	I	R +/+	R +/−	E	N	N	N	N	R +/−	N	R+/−
Adequate patient empowerment (e.g., environmental modification adapted to the person and the degree of impairment)	R −/−	R −/+	R −/+	R +/−	R +/−	R −/+	R +/+	R −/−	R +/+	E	N	R +/−
Critical self-monitoring of carers (e.g., team supervision; strategies to rethink the care process)	R −/−	R −/+	E	R +/−	E	E	R +/+	R +/−	N	R +/+	E	R +/−
Evaluation of abuse and neglect of patients	E	N	N	R +/+	N	N	N	E	N	E	N	R +/−

Special situations for decision-making	Australia	Austria	Canada	United Kingdom	France	Germany	Malaysia	New Zealand	Scotland	Singapore	Switzerland	United States
Sexual relationships (e.g., prevention of abuse or exploitation)	I	N	N	E	N	R −/+	N	I	N	N	N	R +/+
Thorough consideration of the indication for genetic testing (e.g., prevention of stigmatization)	N	R +/+	R +/+	I	E	E	R +/+	I	N	R +/+	I	R +/+
Usage of GPS and other monitoring techniques (e.g., adequately balancing potential benefits, harms, and patient consent)	N	N	N	N	N	N	R +/+	N	N	N	N	N
Particularities in drug treatment (e.g., thorough consideration before prescribing antipsychotic drugs)	E	I	N	R +/−	R +/+	R +/+	R +/−	N	N	R +/+	N	R +/+
Covert medication (e.g., balancing short-term medical benefits versus long-term impact on patient–physician relationship)	N	N	N	E	N	N	N	N	N	N	N	N
Thorough consideration of the indication for brain imaging (e.g., dealing with current lack of evidence for clinical validity of brain imaging in diagnosing dementia)	N	I	I	I	N	E	I	N	I	R +/+	I	I
Usage of physical restraints (e.g., balancing patient's best interests with autonomy)	N	N	N	R +/−	E	N	E	E	N	R +/−	N	R+/+
Tube feeding (e.g., adequate decision making in the patient's best interests)	N	N	N	R +/+	N	R+/−	R +/+	N	N	N	N	N
Adequate end of life/palliative care (e.g., prevention of poor care or neglect)	N	N	N	R +/+	E	N	R +/+	E	N	N	N	E
Dealing with suicidality (e.g., appropriate estimation of quality of life in suicidal patients with dementia)	N	N	N	N	N	N	N	N	N	N	N	R +/−

Rating modalities: N, not addressed; I, implicitly addressed; E, explicitly addressed without recommendation; R, explicitly addressed with recommendation including: justification/citation, +/+; justification/no citation, +/−; no justification/citation, −/+; no justification/no citation, −/−.

Five DSEIs (16%) were addressed explicitly (E or R ratings) in at least 11 out of 12 CPGs, for example “Adequate consideration of complexity of informing patients with dementia” and “Caring for relatives.” When adding all implicit mentions of DSEIs (I, E, or R ratings) 11 DSEIs (35%) were addressed in at least 11 out of 12 CPGs (Table 4).

Though some DSEIs were explicitly addressed in several CPGs, the CPGs varied regarding which aspects of each DSEI were highlighted and in how much detail each was described. As an example, see the original wording examples for the DSEI “Adequate involvement of relatives in the care process” (Table S2). As the wording examples demonstrate, CPGs differed in the specification of (a) whether permission to disclose the diagnosis to carers should or must be sought, (b) which care decisions should be discussed with relatives, and (c) how specific the advice and support for relatives should be.

CPGs further differed in whether a justification and/or citations were given to support a recommendation made in the guideline (Table 5). While the CPGs from Malaysia and Singapore both provided justifications and citations for 71% and 76% of all DSEIs with ethical recommendations (*n* = 12 and *n* = 13), the CPGs from the United Kingdom and the United States provided justifications and citations for only 44% and 45% of all DSEIs with ethical recommendations (*n* = 7 and *n* = 10).

**Table 5:** Rating results for how often a CPG gave a recommendation (R-Rating) along with justification and/or citations with respect to the 31 DSEIs presented.

Clinical Practice Guideline	R-Ratings^a^ (Total)	No Justification, No Citation		Justification, No Citation		No Justification, Citation		Justification, Citation	
		*n*	Percent	*n*	Percent	*n*	Percent	*n*	Percent
Australia (RACGP)	6	3	50%	1	17%	0	0%	2	33%
Austria (CCIV)	12	0	0%	3	25%	3	25%	6	50%
Canada (CMA)	10	2	20%	2	20%	4	40%	2	20%
United Kingdom (NICE)	16	0	0%	8	50%	1	6%	7	44%
France (HAS)	6	1	17%	3	50%	1	17%	1	17%
Germany (DGPPN/DGN)	11	2	18%	5	45%	2	18%	2	18%
Malaysia (MOH Malay.)	17	1	6%	3	18%	1	6%	12	71%
New Zealand (NZGG)	10	1	10%	6	60%	0	0%	3	30%
Scotland (SIGN)	5	0	0%	2	40%	1	20%	2	40%
Singapore (MOH Sing.)	17	1	6%	3	18%	0	0%	13	76%
Switzerland	3	0	0%	3	100%	0	0%	0	0%
United States (APA)	22	3	14%	9	41%	0	0%	10	45%
Mean, %			12%		40%		11%		37%

a R-Rating:explicitly addressed with recommendation

In line with the varying wordings and specifications for the description of a DSEI (Table S2), the arguments chosen for justification of specific ethical recommendations also differed substantially between CPGs. A more in-depth analysis of justification and support patterns for different types of ethical recommendations in CPGs is beyond the scope of this paper. Here we present two rare examples where more or less “precise” justifications were given in a CPG.

For the DSEI “Reasonableness of treatment indications (e.g., risk of overestimating benefit of pharmaceutical treatment)”, the CPG from Singapore recommends:

“Non-pharmacological methods to manage behavioural and psychological symptoms of dementia should be instituted, prior to consideration of pharmacological measures. […] The reason for considering [non-pharmacological treatment] NPT first and as an enduring endeavour in addressing difficult behaviour is two-fold. First, NPTs […] address the underlying reasons for the behaviour. Second, medications carry adverse side-effects and often mask and suppress the behaviour that actually serves to communicate the need of the person with dementia.”

Another example is from the APA guideline regarding the DSEI “Dealing with the need for advanced care planning (e.g., sensibly informing patients and relatives about the types of decision that might need to be made; informing them of tools such as advance directives)”:

“Patients with dementia usually lose the ability to make medical, legal, and financial decisions as the disorder progresses, and consequently these functions must be taken over by others (ref. 97). Clinical evaluation, including cognitive testing when needed, can assist in determining whether a patient with Alzheimer's disease has the capacity to make medical decisions (ref. 98–100). If family members act while the patient is still able to participate, they can seek his or her guidance regarding long-term plans. This approach can help in incorporating the patient's own wishes and values into the decision-making process, as well as in avoiding future conflict.”

## Discussion

This study demonstrates that national CPGs on dementia care (in English and German) already address clinical ethical issues. However, the extent to which the full spectrum of ethical issues in dementia care is represented varies significantly within and among the 12 included CPGs. National guidelines vary in different ways, according to: (a) which ethical issues are represented and which not (for example, the DSEIs “Adequate consideration of existing advance directives in medical decision making,” “Usage of GPS and other monitoring techniques,” and “Dealing with suicidality” were only addressed in one out of 12 CPGs); (b) whether ethical issues are addressed implicitly or explicitly; (c) whether a recommendation on how to deal with a specific ethical issue is included or not; (d) whether a rationale and/or references are provided to explain and justify a specific recommendation; and (e) how thoroughly the ethical issue and/or the respective recommendation is explained.

Recommendations on how to deal with DSEIs often lack the sort of evidence health care professionals employ to justify clinical decisions, such as whether to recommend a specific diagnostic or therapeutic intervention. Nevertheless, ethical recommendations also need appropriate justifications (see the examples given in the Results section). Original literature on dementia ethics tends (with good reason) to employ more argumentation and explanation to justify a specific ethical recommendation [16]–[18]. While CPGs could quite easily be improved with respect to at least basic standards for justification of ethical recommendations, there is a need for further research into how reductive and simplistic the justification of ethical recommendations in future CPGs can and should be [21]. On the one hand, oversimplification can render the content meaningless or unhelpful. On the other hand, in-depth analyses of DSEIs might not fit into the format of CPGs. Many of these challenges are comparable with general challenges in the development of practice-oriented recommendations and guidance [15]. The integration of DSEIs into guidelines, therefore, could use quality assessment tools and standard procedures for guideline development such as the AGREE or GRADE instruments [3],[22].

The Nuffield Council of Bioethics report on ethical issues in dementia care demonstrates how some of the complex issues captured in our DSEI spectrum can be addressed by providing a set of criteria that guide the process of ethical decision-making in dementia care [16]. A good example is the DSEI “Adequate appreciation of the patient (e.g., problems concerning understanding and handling of patient autonomy)”. The Nuffield Council addresses this DSEI as follows: “Wellbeing factors, such as the person's general level of happiness are also important but again cannot automatically take precedence over the person's interests in having their autonomy respected” [16]. In the following, the Nuffield Council suggests factors which should be taken into account when weighing up the conflicting ethical principles in dementia care (wellbeing versus respect of autonomy): “(i) How important is the issue at stake?, (ii) How much distress or pleasure is it causing now?, (iii) Have the underlying values or beliefs on which the earlier preferences were based genuinely changed or can they be interpreted in a new light?, (iv) Do the apparent changes in preferences or values result from psychosocial factors (such as fear) or directly from the dementia (such as sexually disinhibited behaviour), or are they linked with a genuine pleasure in doing things differently?” [16].

The more CPGs are claimed to be key to fostering medical professionalism, and the more these CPGs are broadly accepted as a key resource (by physicians, patients, insurers, hospital managers, and health policy decision makers), the more they should address practice-oriented DSEIs and offer recommendations on how to deal with them. However, it is important to realize that the 31 DSEIs that comprise the matrix for this guideline assessment are only potentially relevant ethical issues for dementia guidelines. The prior systematic review that identified all DSEIs for dementia care was purely descriptive [19]. It cannot be inferred (without further normative justification) that all these DSEIs should be explicitly addressed in every dementia guideline, but the mere fact that the issues have been discussed controversially in analysis papers, editorials, and textbooks is evidence that decision makers need guidance concerning these DSEIs. Guideline development groups need to select the most relevant DSEIs, just as they need to select the most relevant clinical issues.

Guideline development manuals currently fail to address how DSEIs should be included in CPGs. As with any information gathered for inclusion, DSEIs should be incorporated in a systematic, transparent and comprehensible manner. All the usual steps of information retrieval and appraisal must be performed and documented: (a) identification of DSEIs; (b) assessment of relevance; (c) selection of key ethical issues that should be addressed in the CPG; (d) drafting, agreeing and grading text sections that address the DSEI and provide recommendations on how to deal with it.

Our study had the following limitations. The identification and rating of text passages in CPGs that deal with one of the 31 DSEIs unavoidably involves interpretative tasks, which could affect the validity and reliability of the results. We addressed this by having at least two researchers (with education in medicine/public health or bioethics or both) identify and rate text passages independently. In cases of different ratings a third researcher was involved. However, even when this problem is taken into account, we believe that the core findings of this study remain valid and reliable, namely, the low mean rates of explicit coverage of ethical issues in dementia guidelines and the variations in how these guidelines addressed ethical issues. Furthermore, we restricted our analysis to CPGs listed in databases of guidelines, which often employ quality criteria for inclusion. The addition of further guidelines that follow lower standards for evidence-based policy making and stakeholder involvement might result in even lower mean rates for the coverage of DSEIs.

In conclusion, ethical issues were inconsistently addressed in national dementia guidelines, with some guidelines including most and some including few ethical issues (which can be life-determining or important to quality of life). Ethical issues and how to deal with them are important for guidelines to address, for the medical profession to understand how to approach care of patients with dementia, and for patients, their relatives, and the general public, all of whom might seek information and advice in national guidelines.Finally, from a methodological point of view there is a need for further research to specify in how much detail CPGs should address DSEIs and justify recommendations for their handling.

## Supporting Information

Table S1 **Original citations for the ratings in 12 national clinical practice guidelines on dementia care.** Rating codes: N, not addressed; I, implicitly addressed; E, explicitly addressed without recommendation; R, explicitly addressed with recommendation including: justification/citation, +/+; justification/no citation, +/−; no justification/citation, −/+; no justification/no citation, −/−.
(DOC)

Table S2 **Text examples regarding the DSEI “Adequate involvement of relatives in the care process” for a selection of five CPGs.**
(DOC)

## Abbreviations

APA: American Psychiatric Association
CPG: clinical practice guideline
DSEI: disease-specific ethical issue
NICE: National Institute for Health and Clinical Excellence
