# Supplementary material for: Inclusion of Ethical Issues in Dementia Guidelines: A Thematic Text Analysis
Source: PLoS Med. 2013 Aug 13;10(8):e1001498. doi: 10.1371/journal.pmed.1001498 (PMC3742442; doi:10.1371/journal.pmed.1001498)
Supplement: Table S1 — Original citations for the ratings in 12 national clinical practice guidelines on dementia care. Rating codes: N, not addressed; I, implicitly addressed; E, explicitly addressed without recommendation; R, explicitly addressed with recommendation including: justification/citation, +/+; justification/no citation, +/−; no justification/citation, −/+; no justification/no citation, −/−. (DOC) [file pmed.1001498.s001.doc]

Table S1: Original citations for the ratings in 12 national clinical practice guidelines on dementia care.

Rating codes: N = Not addressed; I = Implicitly addressed; E = Explicitly addressed without recommendation; R = explicitly addressed with Recommendation including: justification/citation = +/+; justification/no citation = +/-; no justification/citation = -/+; no justification/no citation = -/-

| **GERMANY (DGPPN/DGN 2009)** | | |
| --- | --- | --- |
| DSEI | Rating | Citation example |
| **1. Diagnosis and medical indication** |  |  |
| Adequate consideration of the complexity of diagnosing dementia (e.g. unclear cut off for MCI (mild cognitive impairment)) | R -/- | “Gleichzeitig erfordert die Frühdiagnostik besondere Sorgfalt, um die Möglichkeit der Stellung einer falsch-positiven Diagnose, die insbesondere früh im Krankheitsverlauf besteht, zu minimieren“ (p. 23) |
| Adequate timing of diagnosis (e.g. guiding an appropriate “breaking bad news” process) | E | “Tätige im medizinischen, therapeutischen, beratenden oder pflegerischen Umfeld von Demenzkranken sind oft unsicher in Fragen zum Umgang mit Erkrankten und Angehörigen oder in Bezug auf den Einsatz sinnvoller Maßnahmen. Dies kann dazu führen, dass die Diagnose einer Demenz nicht oder falsch gestellt wird, und dass Therapie und Hilfe Erkrankten und Angehörigen nicht zukommen.” (p. 11) |
| Reasonableness of treatment indications (e.g. risk of overestimating benefit of pharmaceutical treatment) | R +/- | “Aufgrund der kognitiven Beeinträchtigung des Erkrankten ist neben der Eigenanamnese die Fremdanamnese von zentraler Bedeutung. Die Familien- und Sozialanamnese geben Hinweise auf Risikofaktoren sowie aktuelle Ressourcen und Problemkonstellationen für die Krankheitsbewältigung. Eine genaue Eigen-, Fremd-, Familien- und Sozialanamnese unter Einschluss der vegetativen und Medikamentenanamnese soll erhoben werden. Aus ihr sollen besondere Problembereiche, Alltagsbewältigung und bisheriger Verlauf abschätzbar sein.” (p. 26) |
| Adequate appreciation of the patient (e.g. adequate consideration of the patient as a person; adequate consideration of existing preferences of the patient) | I | „Psychische und Verhaltenssymptome entstehen ferner häufig im interaktionellen Kontext. Um die Verantwortungszuweisung an den Erkrankten zu verhindern, sind solche Symptome als herausforderndes Verhalten konzeptualisiert worden. Hiermit ist gemeint, dass die umgebenden Personen des Erkrankten gefordert sind, ihr Verhalten oder die Umgebung zu reflektieren und zu modifizieren, da das Verhalten des Erkrankten Ausdruck von einer für ihn unangenehmen, schwierigen oder ängstigenden Situation ist. vor diesem konzeptuellen Hintergrund und dem Umstand, dass pharmakologische Behandlung von psychischen und Verhaltenssymptomen begrenzte Wirkung zeigt und im Fall von Antipsychotika zusätzlich mit Risiken für Nebenwirkungen und erhöhter Mortalität assoziiert ist, kommt den psychosozialen Interventionen in diesem Bereich eine besondere Rolle zu.“ (p. 77) |
| **2. Assessing patient decision-making capacity** |  |  |
| Dealing with varying concepts/understanding of “patient’s capacity” among carers | I | „Die Stimulation visueller, auditiver und taktiler Wahrnehmung, Aufmerksamkeit, Konzentration und Orientierung soll über nonverbale und verbale Aktivität kommunikative und soziale Kompetenz fördern.“ (p. 74) |
| Adequate consideration of setting or content in assessing decision-making capacity (e.g. guidance on how to structure the setting for the capacity assessment; involvement of relatives) | E | „Die diagnostische Güte eines neuropsychologischen Kurztestes ist abhängig vom Untersuchungssetting (hohe vs. niedrige Prävalenz der Erkrankung).“ (p. 26) |
| Adequate assessment of patient’s decision-making capacity (e.g. inadequate or biased assessment) | E | „In der täglichen Praxis können kognitive Störungen übersehen werden, falls nicht gezielt nachgefragt wird und keine Angaben von Angehörigen erfolgen.“ (p. 28) |
| **3. Information and disclosure** |  |  |
| Adequate consideration of the complexity of informing patients with dementia (e.g. the amount and manner of provision of information) | R +/- | „Die Patienten und ggf. auch ihre Angehörigen werden über die erhobenen Befunde und ihre Bedeutung im ärztlichen Gespräch in einem der persönlichen Situation des Erkrankten und der Angehörigen angemessenen Rahmen aufgeklärt, wobei sich Art und Inhalt der Aufklärung am individuellen Informationsbedarf und -wunsch sowie am Zustandsbild des Betroffenen orientieren.“ (p. 24) |
| Adequate involvement of relatives in the care process | R -/- | „Entsprechend der Progredienz der Erkrankung ist auch im weiteren Verlauf der Aufklärungs- und Beratungsprozess kontinuierlich fortzusetzen und den wechselnden Bedürfnissen der Demenzkranken und pflegenden Angehörigen anzupassen.“ (p. 24) |
| **4. Decision-making & consent** |  |  |
| Support of patient’s decision-making capacity | R +/- | „Sollte von einem nicht einwilligungsfähigen Erkrankten eine angebotene wirksame, angemessene und verfügbare Therapie, die von der juristischen Vertretungsperson befürwortet wird, abgelehnt werden, soll nach den Ursachen für die Ablehnung (z.B. Missverständnisse, Ängste) gesucht werden. Identifizierten Ursachen sollte mit geeigneten Massnahmen begegnet werden.“ (p. 41) |
| Responsible surrogate decision-making (e.g. interpretation of advances directives from the perspective of the patient; seeking consent among different surrogates prior to making decisions) | I | „Eine Therapieentscheidung soll im Rahmen eines "informed  decision making"-Prozesses von der behandelnden Person und dem Erkrankten sowie ggf. der juristischen Vertretungsperson erzielt werden.“ (p. 41) |
| Dealing with the need for advanced care planning (e.g. sensibly informing patients and relatives about the types of decision that might need to be made; informing them of tools such as advance directives*)* | I | „Der Einsatz von diagnostischen Verfahren setzt aber die Einwilligungsfähigkeit des Betroffenen voraus. Es ist daher im Einzelfall zu prüfen, ob die Einwilligungsfähigkeit für die jeweilige diagnostische Maßnahme vorliegt. Liegt keine Einwilligungsfähigkeit vor, muss die gesetzliche Vertretungssituation geprüft werden (Vorliegen einer Vorsorgevollmacht oder Generalvollmacht, erstellt "in gesunden Tagen" oder einer gesetzlichen Betreuung für Gesundheitsfürsorge), ggf. müssen Maßnahmen ergriffen werden, um eine gesetzliche Vertretungssituation für Fragen der Gesundheitsfürsorge zu schaffen.“ (p. 24) |
| Adequate consideration of existing advance directives in medical decision making (e.g. guidance on how to include advance directives in medical decision making; recommendations on how to deal with conflicts between content of advance directives and present behavior and expressions in patients with severe dementia). | N | n/a |
| **5. Social and context-dependent aspects** |  |  |
| Caring for relatives (e.g. recognizing needs of relatives; preventing distress and burnout) | R +/+ | „Zur Prävention von Erkrankungen, die durch die Pflege und Betreuung hervorgerufen werden, und zur Reduktion von Belastung der pflegenden Angehörigen sollten strukturierte Angebote für Bezugspersonen von Demenzerkrankten vorgesehen werden […]“ (p. 81) |
| Caring for clinical personnel and professional carers (e.g. needs of professionals; preventing distress and burn out) | I | „Tätige im medizinischen, therapeutischen, beratenden oder pflegerischen Umfeld von Demenzkranken sind oft unsicher in Fragen zum Umgang mit Erkrankten und Angehörigen oder in Bezug auf den Einsatz  sinnvoller Maßnahmen. Dies kann dazu führen, dass die Diagnose einer Demenz nicht oder falsch gestellt wird, und dass Therapie und Hilfe Erkrankten und Angehörigen nicht zukommen.“ (p. 11) |
| Adequate assessment of the patient’s potential to harm a third person (e.g. in decisions on the ability to drive) | R +/- | „Eine spezielle Fragestellung, die häufig im diagnostischen Prozess auftritt, betrifft die Eignung des Erkrankten, ein Kraftfahrzeug zu führen. Die Problematik der Fahrtauglichkeit sollte, falls möglich, bereits in der frühen Erkrankungsphase angesprochen werden, um auf einen Verzicht des Fahrens hinzuwirken. [...] Sollte ein Erkrankter bei bestehender Fahruntauglichkeit trotz Aufklärung über die Gefährdung und trotz Aufforderung nicht zu fahren, weiter als Fahrer am Straßenverkehr teilnehmen, so kann ein Arzt trotz seiner grundsätzlichen Schweigepflicht aufgrund einer sorgfältigen Güterabwägung berechtigt sein, zum Schutze der potentiell betroffenen Verkehrsteilnehmer die zuständige Behörde zu benachrichtigen." (p. 24-25) |
| Responsible handling of costs and allocation of limited resources (e.g. avoiding futile diagnostic and treatment) | I | „Bei den Therapieentscheidungen sind Wirksamkeit, Nutzen-Risiko-Abwägungen, Kosten sowie Verfügbarkeit von Verfahren und Ressourcen relevant.“ (p. 41) |
| **6. Care process & process evaluation** |  |  |
| Continuing assessment of potential benefits and harms in individual care strategies | N | n.a. |
| Adequate patient empowerment (e.g. environmental modification adapted to the person and the degree of impairment) | R -/+ | „Sollte von einem nicht einwilligungsfähigen Erkrankten eine angebotene wirksame, angemessene und verfügbare Therapie, die von der juristischen Vertretungsperson befürwortet wird, abgelehnt werden, soll nach den Ursachen für die Ablehnung (z.B. Missverständnisse, Ängste) gesucht werden. Identifizierten Ursachen sollte mit geeigneten Massnahmen begegnet werden.“ (p. 41)  „Spezifische Behandlungsprogramme bewirken bei leicht- bis mittelgradig betroffenen Demenzkranken ähnliche, bis nur mäßig geringfügigere Therapieerfolge hinsichtlich Mobilität und Selbstversorgungsfähigkeit wie bei kognitiv Gesunden.“ (p. 84) |
| Critical self-monitoring of carers (e.g. team supervision; strategies to rethink the care process) | E | „In einer systematischen Übersichtsarbeit über 162 Studien zu psychosozialen Interventionen bei Demenzkranken und psychischen und Verhaltenssymptomen wird Evidenz für einen verbessernden Einfluss von patientenzentriertem Verhaltensmanagement, von Angehörigenedukation und kognitiver Stimulation beschrieben. Diese Verfahren zeigten überdauernde Effekte. Schulungsprogramme für Mitarbeiter in Pflegeeinrichtungen zeigten ebenfalls positive Effekte.“ (p. 78) |
| Evaluation of abuse and neglect of patients | N | n/a |
| **7. Special situations for decision-making** |  |  |
| Sexual relationships (e.g. prevention of abuse or exploitation) | R -/+ | „Phänomene der Disinhibition können bei Demenzkranken auftreten. Dies kann u.a. Sozialverhalten inklusive sexueller Disinhibition, wie auch andere Bereiche, z.B. die Nahrungsaufnahme, betreffen. Statement: Bei enthemmtem Verhalten im Rahmen einer Demenzerkrankung gibt es keine belastbare Evidenz für eine bestimmte Behandlung.“ (p. 72) |
| Thorough consideration of the indication for genetic testing (e.g. prevention of stigmatization) | E | „Bei Verdacht auf eine autosomal dominante Erkrankung sollen eine genetische Beratung und gegebenenfalls eine genetische Testung durchgeführt werden. Dies sollte durch eine humangenetische Beratungsstelle unter Einhaltung entsprechender Vorgaben erfolgen. Eine spezifische therapeutische Implikation für den Betroffenen leitet sich daraus nicht ab. Im Rahmen der Patientenaufklärung ist der Wunsch des Betroffenen bezüglich des möglichen Wissens um das Tragen eines Krankheitsgens zu ermitteln, da dies neben einer diagnostischen Zuordnung der Erkrankung auch Implikationen für die Verwandten des Betroffenen hat. Die Möglichkeit einer psychosozialen Beratung vor und nach der Ergebnismitteilung soll gegeben sein.“ (p. 39) |
| Usage of GPS and other monitoring techniques (e.g. adequately balancing potential benefits, harms, and patient consent) | N | n/a |
| Particularities in drug treatment (e.g. horough consideration before prescribing) | R  +/+ | „Die Gabe von Antipsychotika bei Patienten mit Demenz ist mit einem erhöhten Risiko für Mortalität und für zerebrovaskuläre Ereignisse assoziiert. Patienten und rechtliche Vertreter müssen über dieses Risiko aufgeklärt werden. Die Behandlung soll mit der geringst möglichen Dosis und über einen möglichst kurzen Zeitraum erfolgen. Der Behandlungsverlauf muss engmaschig kontrolliert werden.“ (p. 59) |
| Covert medication (e.g. balancing short-term medical benefits vs. long-term impact on patient–physician relationship) |  | n/a |
| Thorough consideration of the indication for brain imaging (e.g. dealing with current lack of evidence for clinical validity of brain imaging in diagnosing dementia) | E | „Für die Feststellung einer vaskulären Demenz sollten neben der Bildgebung (Ausmaß und Lokalisation von vaskulären Läsionen) Anamnese, klinischer Befund und neuropsychologisches Profil herangezogen werden. Der Beitrag der strukturellen MRT in der Differenzierung der Alzheimer-Demenz oder der frontotemporalen Demenz von anderen neurodegenerativen Demenzen ist bisher nicht ausreichend gesichert.“(p. 43) |
| Usage of physical restraints (e.g. balancing patient’s best interests with autonomy) | N | n/a |
| Tube feeding (e.g. adequate decision making in the patient’s best interests) | R  +/- | „Es liegen keine randomisierten, plazebo-kontrollierten Studien zur Verwendung von PEG-Sonden zur enteralen Ernährung im Stadium der schweren Demenz vor. Basierend auf der bisherigen Datenlage ist eine positive Beeinflussung der Überlebenszeit, der klinischen Symptomatik, des Auftretens von Infektionen oder Dekubitalulzera durch den Einsatz der PEG nicht gegeben.“ (p. 69-70) |
| Adequate end of life/palliative care (e.g. prevention of poor care or neglect) | N | n/a |
| Dealing with suicidality (e.g. appropriate estimation of quality of life in suicidal patients with dementia) | N | n/a |

| **UNITED STATES OF AMERICA (APA 2007)** | | |
| --- | --- | --- |
| DSEI | Rating | Citation example |
| **1. Diagnosis and medical indication** |  |  |
| Adequate consideration of the complexity of diagnosing dementia (e.g. unclear cut off for MCI (mild cognitive impairment)) | R  +/- | “Dementia must be distinguished from malingering and factitious disorder, which generally manifest patterns of cognitive deficits that are inconsistent over time and are uncharacteristic of those typically seen in dementia. Dementia must also be distinguished from milder symptoms. Subjective memory complaints are common as people get older. Many individuals with these complaints have subtle, nonprogressive declines in memory, but some have more significant impairment that is more likely to represent the prodromal phase of Alzheimer’s disease or another dementia. The category of mild cognitive impairment (390) was developed to describe individuals in this prodromal phase (see Section IV.F.2) (6, 391, 397). (p. 44)  “Mild cognitive impairment is a term used to represent a variety of mild cognitive syndromes manifested by a modest but detectable decline in cognitive function in the setting of largely intact functional status (391). Because it is expected that new treatments will be better at preserving than restoring neuronal function, early recognition of these mild syndromes, particularly those thought to represent the prodromal phase of Alzheimer’s disease and other neurodegenerative dementias, are a major focus of current research. Mild cognitive impairment is conceived  of as a transitional state between normal aging and dementia (particularly Alzheimer’s disease) in which cognitive deficits are present but function is preserved. As such, the population of patients meeting the criteria for mild cognitive impairment is inherently unstable, as many patients progress to meet the criteria for dementia. Moreover, because mild cognitive impairment lies along a continuum between normal aging and dementia, its precise upper and lower boundaries are difficult to determine. A variety of research definitions for mild cognitive impairment are in place, but there is no consensus on the optimal definition.” (p. 45) |
| Adequate timing of diagnosis (e.g. guiding an appropriate “breaking bad news” process) | R  +/- | “Family members and other caregivers are a critical source of information, as the patient is frequently unable to give a reliable history, particularly as the disease progresses. Because family members are often responsible for implementing and monitoring treatment plans, their own attitudes and behaviors can have a profound effect on the patient, and they often need the treating physician’s compassion and concern. For these reasons, treatment is directed to the patient-caregiver system. The needs of  caregivers will vary based on factors such as their relationship to the patient, their long-standing role in the family, and their current customs. Clinical judgment is needed to determine the circumstances in which it is appropriate or necessary to speak with caregivers without the patient present, as well as how to proceed with clinical care when here are disputes among family members. A clear process for medical decision making should be delineated for each patient, and a capacity assessment of the patient should be performed when necessary.” (p. 15) |
| Reasonableness of treatment indications (e.g. risk of overestimating benefit of pharmaceutical treatment) | R  +/- | “Family members and other caregivers are a critical source of information, as the patient is frequently unable to give a reliable history, particularly as the disease progresses. Because family members are often responsible for implementing and monitoring treatment plans, their own attitudes and behaviors can have a profound effect on the patient, and they often need the treating physician’s compassion and concern. For these reasons, treatment is directed to the patient-caregiver system. The needs of  caregivers will vary based on factors such as their relationship  to the patient, their long-standing role in the family, and their current customs. Clinical judgment is needed to determine the circumstances in which it is appropriate or necessary to speak with caregivers without the patient present, as well as how to proceed with clinical care when  there are disputes among family members. A clear process for medical decision making should be delineated for each patient, and a capacity assessment of the patient should be performed when necessary.” (p. 15) |
| Adequate appreciation of the patient (e.g. adequate consideration of the patient as a person; adequate consideration of existing preferences of the patient) | R  +/- | “It is also important to individualize the approach to the patient’s needs, and, in this regard, psychiatrists and other mental health care professionals can offer more specific behavioral interventions that caregivers can use to avoid or deal with difficult behaviors. For additional details on such interventions, see Sections II.B.4.b and II.C.4.” (p. 22)  “If family members act while the patient is still able to participate, they can seek his or her guidance regarding long-term plans. This approach can help in incorporating the patient’s own wishes and values into the decisionmaking process, as well as in avoiding future conflict.” (p. 24) “Issues that might be raised related to health care in the later stages of the illness include preferences about medical treatment, the use of feeding tubes, the care desired for infections and other potentially life-threatening medical conditions, and artificial life support. Medical decision making can be transferred in advance to a trusted family member (or friend) in the form of a durable power of attorney for health care or designation of a health care agent. For some patients, a living will or advance directive may also be appropriate, but which document is used and its specific features depend on the prevailing state law.” (p. 24) |
| **2. Assessing patient decision-making capacity** |  |  |
| Dealing with varying concepts/understanding of “patient’s capacity” among carers | E | Patients with dementia usually lose the ability to make medical, legal, and financial decisions as the disorder progresses, and consequently these functions must be taken over by others (97). Clinical evaluation, including  cognitive testing when needed, can assist in determining whether a patient with Alzheimer’s disease has the capacity to make medical decisions. (p. 24) |
| Adequate consideration of setting or content in assessing decision-making capacity (e.g. guidance on how to structure the setting for the capacity assessment; involvement of relatives) | R  -/- | “Often, it may be necessary to conduct a portion of the interview with the caregiver without the patient present, in order to allow for full disclosure of sensitive information.” (p. 16) |
| Adequate assessment of patient’s decision-making capacity (e.g. inadequate or biased assessment) | R  +/+ | “Whatever the intervention, it is critical to match the level of demand on the patient with his or her current capacity, avoiding both infantilization and frustration. Likewise, behavior may also improve by modifying the environment insofar as possible to compensate for the patient’s deficits and to capitalize on his or her strengths (41). In assessing the effectiveness of interventions for problematic behaviors, clinicians can recommend that caregivers maintain a log of specific behaviors as well as their intensity, frequency, precipitants, and consequences.” (p. 19)  “If the activity is a necessary one, for example, bathing, it may be helpful to decrease its frequency or to alter the environment so that the negative consequences are minimized (e.g., switch bath time to allow a home health aid to supervise, or change the location of baths to decrease the impact of aggressive outbursts on family members or other patients). When multistep activities such as dressing and eating precipitate problem behaviors such as aggression, it often helps to simplify the activities (e.g., using clothing with Velcro closures, serving several simple nutritious snacks instead of a large meal).” (p. 19) |
| **3. Information and disclosure** |  |  |
| Adequate consideration of the complexity of informing patients with dementia (e.g. the amount and manner of provision of information) | R  +/+ | “The issue of disclosure of the diagnosis to the patient is complex because  many patients cannot recognize their deficits. Decisions about how to disclose should take into account factors such as cultural issues that might modify the patient’s desire to receive such information (70).” (p. 22) “Cultural differences may affect the family’s perception of cognitive symptoms and therefore their report of them to the physician, as well as attitudes toward treatment (334).” (p. 38)  “Ethnicity, race, and culture may influence interpretation of symptoms as well as attitudes toward nursing home placement; the clinician should be sensitive to varying beliefs about the desirability of such a step (70, 335). Cultural background also has an impact on social networks, caregiving  style, presentation of disease symptoms such as depression, and acceptance of behavioral symptoms.” (p. 38) |
| Adequate involvement of relatives in the care process | R  -/- | “Important aspects of psychiatric management include educating patients and families about the illness, its treatment, and sources of additional care and support (e.g., support groups, respite care, nursing homes, and other long-term-care facilities).” (p. 11)  “Clinical judgment is needed to determine the circumstances in which it is appropriate or necessary to speak with caregivers without the patient present, as well as how to proceed with clinical care when there are disputes among family members. - An important task of the psychiatrist who cares for an individual with dementia is providing or coordinating the education of the patient and family regarding the illness and its natural history. Often the ﬁrst step is to communicate and explain the diagnosis of dementia, including the speciﬁc dementia etiology, if known. Terms should be clariﬁed at the outset to facilitate communication.” (p. 15) |
| **4. Decision-making & consent** |  |  |
| Support of patient’s decision-making capacity | R  +/+ | “Whatever the intervention, it is critical to match the level of demand on the patient with his or her current capacity, avoiding both infantilization and frustration. Likewise, behavior may also improve by modifying the environment insofar as possible to compensate for the patient’s deficits and to capitalize on his or her strengths (41). In assessing the effectiveness of interventions for problematic behaviors, clinicians can recommend that caregivers maintain a log of specific behaviors as well as their intensity, frequency, precipitants, and consequences.” (p. 19)  “If the activity is a necessary one, for example, bathing, it may be helpful to decrease its frequency or to alter the environment so that the negative consequences are minimized (e.g., switch bath time to allow a home health aid to supervise, or change the location of baths to decrease the impact of aggressive outbursts on family members or other patients). When multistep activities such as dressing and eating precipitate problem behaviors such as aggression, it often helps to simplify the activities (e.g., using clothing with Velcro closures, serving several simple nutritious snacks instead of a large meal).” (p. 19) |
| Responsible surrogate decision-making (e.g. interpretation of advances directives from the perspective of the patient; seeking consent among different surrogates prior to making decisions) | R  -/- | “Although the specific laws vary from state to state, advance planning regarding health care and finances can help families avoid the difficulty and expense of petitioning the courts for guardianship or conservatorship should such arrangements become necessary later in the illness.” (p. 24) “Nevertheless, the psychiatrist is encouraged to be familiar with local jurisdictional requirements, because procedures vary by state and some states require judicial review.” (p. 24) |
| Dealing with the need for advanced care planning (e.g. sensibly informing patients and relatives about the types of decision that might need to be made; informing them of tools such as advance directives*)* | R  +/+ | “Important aspects of psychiatric management include [...] advising patients and their families of the need for financial and legal planning due to the patient’s eventual incapacity (e.g., power of attorney for medical and financial decisions, an up-to-date will, and the cost of long-term care). (p. 11)  “The question of referral to a long-term-care facility should be raised well before it becomes an immediate necessity so that families who wish to pursue this option have time to select and apply for a suitable facility, plan for financing long-term care, and make needed emotional adjustments.” (p. 23-24) |
| Adequate consideration of existing advance directives in medical decision making (e.g. guidance on how to include advance directives in medical decision making; recommendations on how to deal with conflicts between content of advance directives and present behavior and expressions in patients with severe dementia). | N | n/a |
| **5. Social and context-dependent aspects** |  |  |
| Caring for relatives (e.g. recognizing needs of relatives; preventing distress and burnout) | R  +/+ | “The decision to remain at home should be reassessed regularly, with consideration of the patient’s clinical status and the continued ability of the patient’s caregivers to care for the patient, manage the burden of care, and utilize available support services.” (p. 15)  “Finally, other key tasks include providing critical support for family members and other caregivers and making referrals to social, legal, and other community resources.” (p. 9)  “Programs have been developed that reduce the burden and lessen the stress and depression associated with longer caregiving. These interventions include psychoeducational programs for coping with frustration or depression; psychotherapy focused on alleviating depression and anxiety, and improving coping; exercise interventions for caregivers; and workshops in stress management techniques.” (p. 22-23)  “End-of-life care for patients with dementia is extremely demanding of family caregivers, with many reporting high levels of depressive  symptoms while caring for their relatives with dementia. However, within 3 months of the death, caregivers experience significant declines in depressive symptoms (372).” (p. 40) |
| Caring for clinical personnel and professional carers (e.g. needs of professionals; preventing distress and burn out) | N | n/a |
| Adequate assessment of the patient’s potential to harm a third person (e.g. in decisions on the ability to drive) | R  +/+ | “Finally, other key tasks include providing critical support for family members and other caregivers and making referrals to social, legal, and other community resources. (p. 9)  “Programs have been developed that reduce the burden and lessen the stress and depression associated with longer caregiving. These interventions include psychoeducational programs for coping with frustration or depression; psychotherapy focused on alleviating depression and anxiety, and improving coping; exercise interventions for caregivers; and workshops in stress management techniques.” (p. 22-23)  “Recommended assessments include evaluation of suicidality, dangerousness to self and others, and the potential for aggression, as well as evaluation of living conditions, safety of the environment, adequacy of supervision, and evidence of neglect or abuse.” (p. 11) |
| Responsible handling of costs and allocation of limited resources (e.g. avoiding futile diagnostic and treatment) | I | “The choice of therapy is generally based on the patient’s characteristics and preference, availability of the therapy, and cost.” (p. 26) |
| **6. Care process & process evaluation** |  |  |
| Continuing assessment of potential benefits and harms in individual care strategies | R  +/- | “Before undertaking an intervention, the psychiatrist should enlist the help of caregivers in carefully characterizing the target symptoms. Their nature, intensity, frequency, precipitants, and consequences should be reviewed and documented. This process is critical to revealing the cause of the symptoms, as well as monitoring the impact of any intervention. This approach also assists caregivers in beginning to achieve some mastery over the problematic symptom. Before embarking on any intervention, it is also helpful if clinicians explicitly review their own, the patient’s, and the caregivers’ expectations.” (p. 18) |
| Adequate patient empowerment (e.g. environmental modification adapted to the person and the degree of impairment) | R  +/- | “Whatever the intervention, it is critical to match the level of demand on the patient with his or her current capacity, avoiding both infantilization and frustration. Likewise, behavior may also improve by modifying the environment insofar as possible to compensate for the patient’s deficits and to capitalize on his or her strengths (41).” (p. 19) |
| Critical self-monitoring of carers (e.g. team supervision; strategies to rethink the care process) | R  +/- | “Before undertaking an intervention, the psychiatrist should enlist the help of caregivers in carefully characterizing the target symptoms. Their nature, intensity, frequency, precipitants, and consequences should be reviewed and documented. This process is critical to revealing the cause of the symptoms, as well as monitoring the impact of any intervention. This approach also assists caregivers in beginning to achieve some mastery over the problematic symptom. Before embarking on any intervention, it is also helpful if clinicians explicitly review their own, the patient’s, and the caregivers’ expectations.” (p. 18) |
| Evaluation of abuse and neglect of patients | R  +/- | “The psychiatrist should be alert to the possibility of elder abuse, financial exploitation, and neglect. Individuals with dementia are at particular risk for abuse because of their limited ability to protest, their lack of comprehension, and the significant demands and emotional strain on caregivers.  Patients whose caregivers appear angry or frustrated may be at even higher risk. Any concern, especially one raised by the patient, must be thoroughly evaluated. Corroborating evidence (e.g., from physical examination) should be sought in order to distinguish delusions, hallucinations,  and misinterpretations from actual abuse. In many states, when neglect or abuse is suspected, the psychiatrist is required to make a report to the appropriate local or state agency responsible for investigating elder abuse.” (p. 20) |
| **7. Special situations for decision-making** |  |  |
| Sexual relationships (e.g. prevention of abuse or exploitation) | R  +/+ | “When male patients display inappropriate sexual behavior, a particular problem in patients with frontal lobe dementias, medroxyprogesterone and related hormonal agents are sometimes recommended (272–274), a recommendation supported only by case series at present. Because  SSRIs may reduce libido and are probably safer, they may be tried before hormonal agents (275).” (p.34) |
| Thorough consideration of the indication for genetic testing (e.g. prevention of stigmatization) | R  +/+ | “However, the role of such testing in clinical practice has not yet been established. Because no preventive treatments are currently available, testing should only be offered in the setting of thorough pre- and posttest counseling (26).” (p. 17)  “Thus, the presence of an APOE4 allele does not change the need for a thorough workup and does not add substantially to diagnostic confidence  (5, 20–22).” (p. 17) |
| Usage of GPS and other monitoring techniques (e.g. adequately balancing potential benefits, harms, and patient consent) | N | n/a |
| Particularities in drug treatment (e.g. horough consideration before prescribing) | R  +/+ | “Psychosis, aggression, and agitation are common in patients with dementia and may respond to similar therapies. When deciding if treatment is indicated, it is critical to consider the safety of the patient and those around him or her [I]. A careful evaluation for general medical, psychiatric,  environmental, or psychosocial problems that may underlie the disturbance should be undertaken [I]. If possible and safe, such underlying causes should be treated first [I]. If this does not resolve the symptoms, and  if they do not cause significant danger or distress to the patient or others, such symptoms are best treated with environmental measures, including reassurance and redirection [I]. For agitation, some of the behavioral measures discussed in Section I.B.2 may also be helpful [II]. If these measures are unsuccessful or the behaviors are particularly dangerous or distressing, then the symptoms may be treated judiciously with one of the agents discussed in the following paragraphs [II]. The use of such agents should be reevaluated and their benefit documented on an ongoing  basis [I]” (p. 13)  “Appropriate use of antipsychotic medications can relieve symptoms  and reduce distress and can increase safety for patients, other residents, and staff [I]. However, their use may be associated with worsening cognitive impairment, oversedation, falls, tardive dyskinesia, and neuroleptic malignant syndrome, as well as with hyperlipidemia, weight gain, diabetes mellitus, cerebrovascular accidents, and death [I]. Thus, good clinical practice requires careful consideration and documentation of the indications and available alternatives, both initially and on a regular ongoing  basis [I]. A dose decrease or discontinuation should be considered periodically for all patients who receive antipsychotic medications [I]. A structured education program for staff may help to both manage patients’ behavior and decrease the use of these medications in nursing  homes [II].“ (p. 14) |
| Covert medication (e.g. balancing short-term medical benefits vs. long-term impact on patient–physician relationship) | N | n/a |
| Thorough consideration of the indication for brain imaging (e.g. dealing with current lack of evidence for clinical validity of brain imaging in diagnosing dementia) | I | “The use of a structural neuroimaging study, such as computerized  tomography or magnetic resonance imaging (MRI) scan, is generally recommended as part of an initial evaluation, although clinical practice varies […]” (p. 16) |
| Usage of physical restraints (e.g. balancing patient’s best interests with autonomy) | R  +/+ | “Within hospital or nursing home settings, physical restraints (e.g., Posey restraints, geri-chairs) are sometimes used to treat agitation or combativeness that puts the patient or others at risk. Nonetheless, principles of humane care as well as federal regulations support minimizing restraint use as much as possible. In addition, some evidence suggests that restraints may increase the risk of falls and contribute to cognitive decline (42, 43) and that reducing restraint use can decrease the rate of serious injuries among nursing home residents (44).” (p. 19) |
| Tube feeding (e.g. adequate decision making in the patient’s best interests) | N | n/a |
| Adequate end of life/palliative care (e.g. prevention of poor care or neglect) | E | “End-of-life care for patients with dementia is extremely demanding of family caregivers, with many reporting high levels of depressive  symptoms while caring for their relatives with dementia. However, within 3 months of the death, caregivers experience significant declines in depressive symptoms (372).” (p. 40)  “Hospice care is an underused resource for patients with end-stage dementia (109, 110). Hospice provides physical support for the patient (with an emphasis on attentive nursing care and relief of discomfort rather than medical intervention) and emotional support for the family during the last months of life. A physician must certify that the patient meets hospice criteria for admission for hospice benefits to be available (111).” (p. 26) |
| Dealing with suicidality (e.g. appropriate estimation of quality of life in suicidal patients with dementia) | R  +/- | “Recommended assessments include evaluation of suicidality, dangerousness to self and others, and the potential for aggression, as well as evaluation of living conditions, safety of the environment, adequacy of supervision, and evidence of neglect or abuse.” (p. 11)  “All patients (and their caregivers) should be asked about the presence of wishes for death, suicidal ideation, suicide plans, as well as a history of previous self-injurious behavior. If suicidal ideation occurs in patients with dementia, it tends to be earlier in the disease, when insight is more likely to be preserved. Interventions to address suicidal ideation are similar to those for patients without dementia and include psychotherapy; pharmacotherapy; removal of potentially dangerous items such as medications, guns, or vehicles; increased supervision; and hospitalization.” (p. 18) |

| **MALAYSIA (MINISTRY OF HEALTH 2009)** | | |
| --- | --- | --- |
| DSEI | Rating | Citation example |
| **1. Diagnosis and medical indication** |  |  |
| Adequate consideration of the complexity of diagnosing dementia (e.g. unclear cut off for MCI (mild cognitive impairment)) | E | “Mental state examination would encompass assessment of cognitive function which can be made by using brief cognitive instruments, or a more detailed neuropsychological assessments administered by a clinician or neuropsychologist. There are many brief cognitive tests that can be used to obtain an objective measure of the cognitive impairment.” (p. 30) |
| Adequate timing of diagnosis (e.g. guiding an appropriate “breaking bad news” process) | R  +/+ | “Most physicians are uncomfortable about disclosure and often would use the patient’s relatives and caregivers as a proxy when considering discussion of the diagnosis, treatment and prognosis of the illness, without necessarily having the patient’s consent [….] Studies have shown that the vast majority of patients with mild dementia wished to be fully informed […] patients with dementia should be informed early because of the possibility to plan their lives.” (p. 98-99) |
| Reasonableness of treatment indications (e.g. risk of overestimating benefit of pharmaceutical treatment) | R  +/- | “Although AChEI is recommended only to people with AD of mild to moderate severity, healthcare professionals should not rely on MMSE scores in deciding treatment initiation.” (p. 53) |
| Adequate appreciation of the patient (e.g. adequate consideration of the patient as a person; adequate consideration of existing preferences of the patient) | R  +/+ | The aim of non-pharmacological treatment or psychosocial intervention is to enhance the person’s self and quality of life. There is no single optimal psychosocial approach available, and thus a multi-dimensional approach is essential to ensure effective interventions. This approach should be person-centred and has to be individualised and tailored to the person’s needs, personality, biography, goals, strengths and preferences. (p. 77)  “There is little research to draw clear conclusions on specific interventions for promoting independence. Despite this conclusion, several strategies are recommended to promote independence. The activities chosen must be individualised according to patient’s needs, strengths and limitations.” (p. 78) |
| **2. Assessing patient decision-making capacity** |  |  |
| Dealing with varying concepts/understanding of “patient’s capacity” among carers | E | “A diagnosis of dementia does not automatically imply a loss of decision making capacity, which is specific to each patient and to each medical decision.[45] Capacity can be assessed by the patient’s ability to:  v Understand the information relevant to the decision.  v To retain this information  v To use or weigh that information as part of the process of making the  decision or  v To communicate his decision (whether by talking, using sign language or  any other means)” (p. 100) |
| Adequate consideration of setting or content in assessing decision-making capacity (e.g. guidance on how to structure the setting for the capacity assessment; involvement of relatives) | R  +/+ | “A diagnosis of dementia does not automatically imply a loss of decision making capacity, which is specific to each patient and to each medical decision.[45] Capacity can be assessed by the patient’s ability to:  v Understand the information relevant to the decision.  v To retain this information  v To use or weigh that information as part of the process of making the  decision or  v To communicate his decision (whether by talking, using sign language or  any other means)” (p. 100)  “There is little research to draw clear conclusions on specific interventions for promoting independence. Despite this conclusion, several strategies are recommended to promote independence. The activities chosen must be individualised according to patient’s needs, strengths and limitations.” (p. 78) |
| Adequate assessment of patient’s decision-making capacity (e.g. inadequate or biased assessment) | R  +/+ | “A diagnosis of dementia does not automatically imply a loss of decision making capacity, which is specific to each patient and to each medical decision.[45] Capacity can be assessed by the patient’s ability to:  v Understand the information relevant to the decision.  v To retain this information  v To use or weigh that information as part of the process of making the  decision or  v To communicate his decision (whether by talking, using sign language or  any other means)” (p. 100)  “Environment plays an important role in the development of behaviour problem in dementia. Most people suffering from dementia in this country are still cared for at home by their family members, but institutional care may be inevitable as the disease progresses with changes in the social and family structure.” (p. 91) |
| **3. Information and disclosure** |  |  |
| Adequate consideration of the complexity of informing patients with dementia (e.g. the amount and manner of provision of information) | E | “The ECAQ is specifically designed to assess cognition among the elderly population in developing countries, taking into account the cultural difference and the relatively low literacy rate.” (p. 26)  “It is not uncommon in most Asian cultures that the elderly is refrained from more complex activities, and for others to take over the task for them.” (p. 78) |
| Adequate involvement of relatives in the care process | R +/- | “Following disclosure to patient, support should be provided to patients and caregivers.” (p. 99)  “Both PWD and their caregiver are entitled to receive relevant information regarding dementia, treatment, available support services, as well as legal, financial and benefits advice.” (p. 140) |
| **4. Decision-making & consent** |  |  |
| Support of patient’s decision-making capacity | R  -/- | “Environment plays an important role in the development of behaviour problem in dementia. Most people suffering from dementia in this country are still cared for at home by their family members, but institutional care may be inevitable as the disease progresses with changes in the social and family structure.” (p. 91) |
| Responsible surrogate decision-making (e.g. interpretation of advances directives from the perspective of the patient; seeking consent among different surrogates prior to making decisions) | I | “Individual care plans should be developed and recorded in patients’ notes. These should be reviewed regularly with caregivers and other staff.” (p. 77)  “The PWD and their caregivers should be educated on the use of advance statements (which allow people to state what is to be done if they should subsequently lose the capacity to decide or to communicate) and advance decisions to refuse treatment. When a people with dementia loses decision making capacity, it is important to protect the person from their own harmful decisions or actions. (p. 100) |
| Dealing with the need for advanced care planning (e.g. sensibly informing patients and relatives about the types of decision that might need to be made; informing them of tools such as advance directives*)* | R (-/+) | “The PWD and their caregivers should be educated on the use of advance statements (which allow people to state what is to be done if they should subsequently lose the capacity to decide or to communicate) and advance decisions to refuse treatment. When a people with dementia loses decision making capacity, it is important to protect the person from their own harmful decisions or actions. (p. 100) |
| Adequate consideration of existing advance directives in medical decision making (e.g. guidance on how to include advance directives in medical decision making; recommendations on how to deal with conflicts between content of advance directives and present behavior and expressions in patients with severe dementia). | N | n/a |
| **5. Social and context-dependent aspects** |  |  |
| Caring for relatives (e.g. recognizing needs of relatives; preventing distress and burnout) | R  +/+ | “Caring for a PWD is very challenging, but it can be equally rewarding. The  role of the caregiver has been increasingly recognised as the care of the PWD is dependent on them. There is evidence to show an increase in potential costs, both direct and indirect, as a result of psychological distress, physical and mental ill-health, as well as increased mortality among the caregivers. In addition, interventions of caregivers would also benefit the patient, in terms of reducing BPSD, and nursing home placement. Hence the management of dementia should also focus on helping the caregivers. A “one-size-fits-all” approach is deemed ineffective while specific interventions that are tailored towards the needs of the caregiver, implemented at the relevant trajectories of illness, would be most effective.” (p. 93) |
| Caring for clinical personnel and professional carers (e.g. needs of professionals; preventing distress and burn out) | R  +/+ | “It had been demonstrated that interventions which involve the PWD along side the caregiver appear more effective than interventions that involve the caregiver alone. Among the components of interventions, while psychological therapies had the best outcome on caregiver depression, multi-component interventions had more robust effect on caregiver burden and well-being. Evidence suggests that caregiver intervention can delay the need for nursing home placement.” (p. 95) |
| Adequate assessment of the patient’s potential to harm a third person (e.g. in decisions on the ability to drive) | N | n/a |
| Responsible handling of costs and allocation of limited resources (e.g. avoiding futile diagnostic and treatment) | N | n/a |
| **6. Care process & process evaluation** |  |  |
| Continuing assessment of potential benefits and harms in individual care strategies | N | n/a |
| Adequate patient empowerment (e.g. environmental modification adapted to the person and the degree of impairment) | R  +/+ | “ADL skill training involves assessing people’s abilities, their impairments and their task performance in order to understand the underlying physical, psychosocial and neurological issues.” (p. 79)  “Environmental modification is helpful but needs to be individualised to the person and the degree of impairment, preferably after occupational therapist assessment.” (p. 92, further p. 139) |
| Critical self-monitoring of carers (e.g. team supervision; strategies to rethink the care process) | R  +/+ | “Professionals trained in assessment and care planning can devise ADL  skill training programmes for use by caregivers and/or care staff. (p. 79)  “Pain is a common symptom and there is evidence that pain goes undetected amongst PWD. This reflects difficulties with communication and the recognition of pain by clinicians. There is evidence of under-treatment of pain.” (p. 102) |
| Evaluation of abuse and neglect of patients | N | n/a |
| **7. Special situations for decision-making** |  |  |
| Sexual relationships (e.g. prevention of abuse or exploitation) | N | n/a |
| Thorough consideration of the indication for genetic testing (e.g. prevention of stigmatization) | R  +/+ | “Given all these uncertainties, genetic screening for the APOE genotype  in asymptomatic individuals in the general population is not recommended due to low specificity and sensitivity. Level III, good Patients suspected of having familial Early onset AD should, however be referred to a memory or genetic clinic for further evaluation.” (p. 8) |
| Usage of GPS and other monitoring techniques (e.g. adequately balancing potential benefits, harms, and patient consent) | R  +/+ | “Electronic tagging and tracking devices such as mobile phone technology and Global Positioning System (GPS) may be viewed as a way of creating a more secure environment for vulnerable PWD who are at risk. However, to date no RCT have been carried out to look into the efficacy of electronic tagging. The use of telecommunication system and computer technology may also be used to support distance caregiving, although this has not been fully utilised. Similarly, low-level technology (for example lights attached to a movement sensor) is widely used in adaptive aids to minimise risk without the need for action by the user. (p. 80) |
| Particularities in drug treatment (e.g. horough consideration before prescribing) | R  +/- | “Antibiotics for treatment of infections and pneumonia in severe and late  dementia should be individualised taking into consideration of the severity of dementia, co-morbidity, nutritional status, mobility status and virility of the organism. (Grade C)” (p. 104) |
| Covert medication (e.g. balancing short-term medical benefits vs. long-term impact on patient–physician relationship) | N | n/a |
| Thorough consideration of the indication for brain imaging (e.g. dealing with current lack of evidence for clinical validity of brain imaging in diagnosing dementia) | I | “Structural neuroimaging such as CT scan of the brain and MRI should be done where available in the evaluation of dementia to rule out intracranial pathology. (Grade A) MRI is the modality of choice to assist with early diagnosis and to detect subcortical vascular changes. (Grade C)  Functional imaging and neuroimaging markers are not recommended routinely in the diagnosis of dementia. (Grade C)” (p. 45-46) |
| Usage of physical restraints (e.g. balancing patient’s best interests with autonomy) | E | “However the use of restraints can cause some potential problems including risk of harm and injury, further cognitive and functional decline, leading to ultimately loss of independence, as well as loss of confidence and self esteem.” (p. 104) |
| Tube feeding (e.g. adequate decision making in the patient’s best interests) | R  +/+ | “The challenge is whether or not artificial nutrition and hydration should be instituted. However artificial nutrition and hydration is not without problems as findings had shown that it could result burdensome complications such as aspiration pneumonia and pressure sores. Patients often have to be restrained during artificial nutrition and hydration, and this may also prolong survival. - A review of the evidence found no relevant RCTs comparing tube feeding and oral feeding. On the available data, it was concluded that the best evidence did not support the use of tube feeding in dementia. There is a need to weigh the risk and benefit of artificial nutrition and hydration, as it is seldom warranted for patients in the final stages of dementia. Food thickeners with appropriate posture and feeding techniques can be used in patients with dysphagia. - A survey of 376 severely demented patients (Global Deterioration Scale Level 7) showed that 24.5% (92) were fed by nasogastric or gastrostomy tube. Despite reservation concerning its utility, feeding tube is reasonably widespread in patients who have reached the stage of severe dementia. - In an observational study of severe dementia (n=178, six weeks duration), a high level of discomfort was found at the time of decision to forgo artificial nutrition and hydration, but the discomfort reduced days thereafter. More than half of the patients (59%) died within one week after the decision was made to forgo artificial nutrition and hydration.” (p. 101-102) |
| Adequate end of life/palliative care (e.g. prevention of poor care or neglect) | R  +/+ | “Palliative care in dementia should begin from the time of diagnosis, up to the time of death. Communication about these issues should start as soon as possible, ideally at time when the diagnosis of dementia was made because individuals with dementia eventually lose the ability to make independent decisions about their future care. However, doctors are reluctant to adopt adequate palliative care for people with dementia.  A case control study (n=303) comparing palliative care measures  (pain medications and oxygen) in PWD found that only 11% of were given palliative care measures compared to 38% of subjects who were not demented. This finding did not vary significantly with the severity of dementia [321] Level II-3,good” (p. 101) |
| Dealing with suicidality (e.g. appropriate estimation of quality of life in suicidal patients with dementia) | N | n/a |

| **UNITED KINGDOM (NICE 2011)** | | |
| --- | --- | --- |
| DSEI | Rating | Citation example |
| **1. Diagnosis and medical indication** |  |  |
| Adequate consideration of the complexity of diagnosing dementia (e.g. unclear cut off for MCI (mild cognitive impairment)) | R  +/+ | “However, the tools for identifying the early changes of AD and other dementias are outpacing the therapeutic options and so the usefulness of such very early ‘pre-clinical’ diagnosis currently remains uncertain (Chang & Silverman, 2004).” (p. 144)  “The means of identification of the early changes of dementia syndromes are developing more rapidly than the therapeutic options and so the usefulness of such very early pre-clinical diagnosis currently remains uncertain.” (p. 145) |
| Adequate timing of diagnosis (e.g. guiding an appropriate “breaking bad news” process) | R  +/+ | “The moment of sharing the diagnosis may not be comfortable for any of those concerned – neither the clinician, nor the person with dementia, nor his or her carer (Friel McGowan, 1993).” (p. 87)  “The majority of people with mild dementia wish to know of their diagnosis (Pinner & Bouman, 2003; De Lepeleire et al., 2004), and all practitioners should assume that the diagnosis will be discussed with the person with dementia, unless there are clear reasons not to do so. The benefits of sharing a diagnosis include ending uncertainty, confirming suspicions, increasing understanding of problems, giving access to support, promoting positive coping strategies, facilitating planning and fulfilment of short-term goals (Bamford et al., 2004; Pratt & Wilkinson, 2003; Husband, 1999, 2000; Smith & Beattie, 2001).” (p. 156)  “People with severe dementia are often not given their diagnosis, although family carers are likely to receive it (Fahy et al., 2003). There may be issues around lack of insight and difficulties in retaining information, which make conveying information about diagnosis difficult if not impossible in such situations. However, this should be assessed and, wherever possible, responded to on an individual basis. There should be no automatic assumption that diagnosis should not be conveyed to the individual  simply because of the perceived severity of dementia.” (p. 156) |
| Reasonableness of treatment indications (e.g. risk of overestimating benefit of pharmaceutical treatment) | I | “Diagnosis of dementia can only be made after a thorough assessment consisting of history, cognitive and mental state examination, physical examination and appropriate investigations. A detailed history from the person and, as the person may not be able to give a fully accurate history, a history from a relative or someone who knows the person well should be obtained, including the history of the presenting complaint, past medical and psychiatric history, medication use, drug or alcohol history, family  medical and psychiatric history, a history of changes in personality or behaviour and assessment of changes in abilities to undertake everyday tasks.” (p. 149) |
| Adequate appreciation of the patient (e.g. adequate consideration of the patient as a person; adequate consideration of existing preferences of the patient) | R  +/- | “Health and social care staff should identify the specific needs of people  with dementia and their carers arising from ill health, physical disability,  sensory impairment, communication difficulties, problems with nutrition,  poor oral health and learning disabilities. Care plans should record and  address these needs.” (p. 13)  “Health and social care staff, especially in residential settings, should identify and, wherever possible, accommodate the preferences of people with dementia and their carers, including diet, sexuality and religion. Care plans should record and address these preferences.” (p. 13)  “Care managers and care coordinators should ensure that care plans are  based on an assessment of the person with dementia’s life history, social  and family circumstance, and preferences, as well as their physical and  mental health needs and current level of functioning and abilities.” (p. 15)  “Health and social care staff should identify the specific needs of people  with dementia and their carers arising from diversity, including gender,  ethnicity, age (younger or older), religion and personal care. Care plans  should record and address these needs.” (p. 215) |
| **2. Assessing patient decision-making capacity** |  |  |
| Dealing with varying concepts/understanding of “patient’s capacity” among carers | I | “Individuals must be given all available support before it is concluded that they cannot make decisions for themselves.” (p. 7) |
| Adequate consideration of setting or content in assessing decision-making capacity (e.g. guidance on how to structure the setting for the capacity assessment; involvement of relatives) | I | “The complexity of activity and level of engagement will change; however, it should not be assumed that the person with dementia does not retain abilities to perform an activity. Individualised and creative ways need to be explored to maximise the use of an individual’s strengths well into the later stages of dementia.” (p. 165) |
| Adequate assessment of patient’s decision-making capacity (e.g. inadequate or biased assessment) | E | “Health and social care professionals should always seek valid consent from people with dementia. This should entail informing the person of options, and checking that he or she understands, that there is no coercion and that he or she continues to consent over time. If the person lacks the capacity to make a decision, the provisions of the Mental Capacity Act 2005 must be followed.” (p. 11) |
| **3. Information and disclosure** |  |  |
| Adequate consideration of the complexity of informing patients with dementia (e.g. the amount and manner of provision of information) | R  +/+ | “Following a diagnosis of dementia, health and social care professionals should, unless the person with dementia clearly indicates to the contrary, provide them and their family with written information about: ● the signs and symptoms of dementia ● the course and prognosis of the condition ● treatments ● local care and support services ● support groups ● sources of financial and legal advice, and advocacy ● medico-legal issues, including driving ● local information sources, including libraries and voluntary organisations.” (p. 24)  “Sensitivity is required in ensuring that information about the diagnosis is given in a way that is easily understood by the person concerned and acceptable to the family. Gentle questioning at an early stage will help to ascertain what people can, and want, to be told. There is much we can learn from earlier work on sharing the diagnosis with people with cancer (for example, Buckman, 1996).” (p. 88)  “In brief, for consent to be valid it must be: ● informed ● competent ● uncoerced ● continuing. Each of these concepts requires interpretation and judgement, as none of them is entirely unproblematic (Department of Health, 2001a). For instance, people can be more or less informed. [...] Department of Health guidelines (Department of Health, 2001c) have pointed out that, although informing patients about the nature and purpose of procedures may be enough to avoid a claim of battery, it may not be sufficient to fulfill the legal duty of care. There may be other pieces of information relevant to the individual patient that it would be negligent not to mention. Hence the General Medical Council (GMC)’s insistence that doctors should do their best ‘to find out about patients’ individual.” (p. 90) |
| Adequate involvement of relatives in the care process | R  +/+ | “Following the disclosure of the diagnosis, people with dementia and their families may want further support and opportunities for talking. Pre- and post-assessment counselling services should be part of the specialist memory assessment service. Recent work (Cheston et al., 2003a) has shown the value of psychotherapeutic support groups for people with dementia, allowing them space to share their feelings with others. Joint interventions with the person with dementia and family carers, such as family therapy, recognise the fact that the diagnosis does not impact on just oneperson but on a whole family system (Gilleard, 1996).” (p. 88)  “Health and social care professionals should inform people with dementia and their carers about advocacy services and voluntary support, and should encourage their use. If required, such services should be available for both people with dementia and their carers independently of each other.” (p. 109)  “The experience of the diagnosis of dementia is challenging both for people with dementia and family members and for healthcare professionals, so healthcare professionals should make time available to discuss the diagnosis and its implications with the person with dementia and also with family members (usually only with the consent of the person with dementia). Healthcare professionals should be aware that people with dementia and family members may need ongoing support to cope with the difficulties presented by the diagnosis.” (p. 163) |
| **4. Decision-making & consent** |  |  |
| Support of patient’s decision-making capacity | E | “Health and social care professionals should always seek valid consent from people with dementia. This should entail informing the person of options, and checking that he or she understands, that there is no coercion and that he or she continues to consent over time. If the person lacks the capacity to make a decision, the provisions of the Mental Capacity Act 2005 must be followed.” (p. 11)  “The complexity of activity and level of engagement will change; however, it should not be assumed that the person with dementia does not retain abilities to perform an activity. Individualised and creative ways need to be explored to maximise the use of an individual’s strengths well into the later stages of dementia.” (p. 165) |
| Responsible surrogate decision-making (e.g. interpretation of advances directives from the perspective of the patient; seeking consent among different surrogates prior to making decisions) | I | “The experience of the diagnosis of dementia is challenging both for people with dementia and family members and for healthcare professionals, so healthcare professionals should make time available to discuss the diagnosis and its implications with the person with dementia and also with family members (usually only with the consent of the person with dementia).” (p. 163) |
| Dealing with the need for advanced care planning (e.g. sensibly informing patients and relatives about the types of decision that might need to be made; informing them of tools such as advance directives*)* | R  -/+ | “They should be given a choice of treatments and need information about practical support and entitle ments, like Lasting Powers of Attorney and advance decisions to refuse treatment (more information can be found in Section 4.9.4 and in the Mental Capacity Act 2005 [The Stationery Office, 2005]).” (p. 87)  “Health and social care professionals should inform people with dementia and their carers about advocacy services and voluntary support, and should encourage their use. If required, such services should be available for both people with dementia and their carers independently of each other.” (p. 109)  “Health and social care professionals should discuss with the person with  dementia, while he or she still has capacity, and his or her carer the use of: ● advance statements (which allow people to state what is to be done if they should subsequently lose the capacity to decide or to communicate) ● advance decisions to refuse treatment ● Lasting Power of Attorney (a legal document that allows people to state in writing who they want to make certain decisions for them if they cannot make them for themselves, including decisions about personal health and welfare) ● a Preferred Place of Care Plan (which allows people to record decisions about future care choices and the place where the person would like to die).” (p. 110) |
| Adequate consideration of existing advance directives in medical decision making (e.g. guidance on how to include advance directives in medical decision making; recommendations on how to deal with conflicts between content of advance directives and present behavior and expressions in patients with severe dementia). | N | n/a |
| **5. Social and context-dependent aspects** |  |  |
| Caring for relatives (e.g. recognizing needs of relatives; preventing distress and burnout) | R  +/- | “Health and social care managers should ensure that the rights of carers to receive an assessment of needs, as set out in the Carers and Disabled Children Act 2000 and the Carers (Equal Opportunities) Act 2004, are upheld. Carers of people with dementia who experience psychological distress and negative psychological impact should be offered psychological therapy, including cognitive behavioural therapy, conducted by a specialist practitioner.” (p. 11)  “Health and social care staff should identify the specific needs of people with dementia and their carers arising from ill health, physical disability,sensory impairment, communication difficulties, problems with nutrition, poor oral health and learning disabilities.” (p. 13)  “Those carrying out carers’ assessment should seek to identify any psychological distress and the psychosocial impact on the carer. This should be an ongoing process and should include any period after the person with dementia has entered residential care.” (p. 35)  “Care plans for carers of people with dementia should involve a range of tailored interventions. These may consist of multiple components  including: ● individual or group psychoeducation ● peer-support groups with other carers, tailored to the needs of individuals depending on the stage of dementia of the person being cared for and other characteristics ● support and information by telephone and through the internet ● training courses about dementia, services and benefits, and communication and problem solving in the care of people with dementia ● involvement of other family members as well as the primary carer in family meetings.” (p. 35)  “Those providing care for people with dementia are one of the most vulnerable groups of carers and often have high levels of stress, feelings of guilt, depression and other psychological problems. They often ignore their own health needs in favour of those of the person for whom they care. They may become exhausted, have poor physical health and feel isolated.” (p. 37) |
| Caring for clinical personnel and professional carers (e.g. needs of professionals; preventing distress and burn out) | I | “The experience of the diagnosis of dementia is challenging both for people with dementia and family members and for healthcare professionals, so healthcare professionals should make time available to discuss the diagnosis and its implications with the person with dementia and also with family members (usually only with the consent of the person with dementia).” (p. 163) |
| Adequate assessment of the patient’s potential to harm a third person (e.g. in decisions on the ability to drive) | R  +/- | “People with dementia who develop non-cognitive symptoms or behaviour that challenges should be offered a pharmacological intervention in the first instance only if they are severely distressed or there is an immediate risk of harm to the person or others. The assessment and care-planning approach, which includes behavioural management, should be followed as soon as possible (see recommendation 1.7.1.1). If distress and/or agitation are less severe, the interventions described in recommendations 1.7.1.2, 1.8.1.2 and 1.8.1.3 should be followed before a pharmacological intervention is considered.” (p. 260)  “Health and social care staff who care for people with dementia should identify, monitor and address environmental, physical health and  psychosocial factors that may increase the likelihood of behaviour that challenges, especially violence and aggression, and the risk of harm to self or others. These factors include: ● overcrowding ● lack of privacy ● lack of activities ● inadequate staff attention ● poor communication between the person with dementia and staff ● conflicts between staff and carers ● weak clinical leadership.” (p. 262-263)  “Up to 75% of people with dementia may be affected by non-cognitive symptoms/behaviour that challenges. They are a leading cause of distress to carers and often lead to the institutionalisation of the person with dementia.” (p. 36) |
| Responsible handling of costs and allocation of limited resources (e.g. avoiding futile diagnostic and treatment) | I | “People with dementia should not be excluded from any services because of their diagnosis, age (whether designated too young or too old) or coexisting learning disabilities. (p. 11)  “Schneider and colleagues (2003) warn of the potential to significantly underestimate the total cost of care if informal care costs are not included, estimating that informal care accounts for up to 40% of the total cost of dementia. Caring for the person with dementia at home can mean that up to 75% of associated care costs are out-of-pocket expenses and informal care (Bosanquet et al., 1998).” (p. 107)  “Providing care for people with dementia requires a wide range of services from a wide range of sources. Although the exact economic cost of dementia is unknown because of the informal nature of a large amount of the care provided, it can be concluded that dementia has substantial economic implications.” (p. 108) |
| **6. Care process & process evaluation** |  |  |
| Continuing assessment of potential benefits and harms in individual care strategies | R  +/- | “Care managers and care coordinators should ensure the coordinated delivery of health and social care services for people with dementia. This should involve: ● a combined care plan agreed by health and social services that takes into account the changing needs of the person with dementia and his or her carers.” (p. 131)  “People with dementia who develop non-cognitive symptoms that cause them significant distress or who develop behaviour that challenges should be offered an assessment at an early opportunity to establish the likely factors that may generate, aggravate or improve such behaviour. The assessment should be comprehensive and include: ● the person’s physical health ● depression ● possible undetected pain or discomfort ● side effects of medication ● individual biography, including religious beliefs and spiritual and cultural identity ● psychosocial factors ● physical environmental factors ● behavioural and functional analysis conducted by professionals with specific skills, in conjunction with carers and care workers. Individually tailored care plans that help carers and staff address the behaviour that challenges should be developed, recorded in the notes and reviewed regularly. The frequency of the review should be agreed by the carers and staff involved and written in the notes.” (p. 260) |
| Adequate patient empowerment (e.g. environmental modification adapted to the person and the degree of impairment) | R  +/- | “As function deteriorates, it is not uncommon for people with dementia to withdraw from more complex activity and social environments and for others to want to perform tasks for them. However, the literature suggests that functioning in activities of daily living often deteriorates below what would be expected by the illness alone (Tappen, 1994; Beck et al., 1997). Therefore the person with dementia, care providers, family and friends should consider opportunities to maintain an active life and social roles and to promote independence beginning in the early stages of the condition. When exploring appropriate activities, it is also important to consider the right level of stimulation and challenge for the individual. The complexity of activity and level of engagement will change; however, it should not be assumed that the person with dementia does not retain abilities to perform an activity. Individualised and creative ways need to be explored to maximise the use of an individual’s strengths well into the later stages of dementia.” (p. 165)  “People with dementia who develop non-cognitive symptoms that cause them significant distress or who develop behaviour that challenges should be offered an assessment at an early opportunity to establish the likely factors that may generate, aggravate or improve such behaviour. The assessment should be comprehensive and include: ● the person’s physical health ● depression ● possible undetected pain or discomfort ● side effects of medication v individual biography, including religious beliefs and spiritual and cultural identity ● psychosocial factors ● physical environmental factors ● behavioural and functional analysis conducted by professionals with specific skills, in conjunction with carers and care workers. Individually tailored care plans that help carers and staff address the behaviour that challenges should be developed, recorded in the notes and reviewed regularly. The frequency of the review should be agreed by the carers and staff involved and written in the notes.” (p. 260)  “Health and social care staff should encourage people with dementia to eat and drink by mouth for as long as possible. Specialist assessment and advice concerning swallowing and feeding in dementia should be available. Dietary advice may also be beneficial.” (p. 262-263) |
| Critical self-monitoring of carers (e.g. team supervision; strategies to rethink the care process) | R  +/- | “Health and social care staff working in care environments where younger people are at risk of developing dementia, such as those catering for people with learning disabilities, should be trained in dementia awareness.” (p. 14)  “When developing educational programmes for different health and social  care staff, trainers should consider the following elements, combined according to the needs of the staff being trained (if staff care for people with learning disabilities, the training package should be adjusted accordingly).” (p. 16)  “Healthcare professionals who regularly diagnose dementia and discuss this with people with the condition and carers should consider mentoring or providing clinical supervision to less experienced colleagues.” (p. 24)  “Health and social care managers should ensure that all staff working with older people in the health, social care and voluntary sectors have access to dementia-care training (skill development) that is consistent with their roles and responsibilities.” (p. 292-293) |
| Evaluation of abuse and neglect of patients | R  +/+ | “Because people with dementia are vulnerable to abuse and neglect, all health and social care staff supporting them should receive information and training about, and abide by the local multi-agency policy on, adult protection.” (p. 111)  “The vulnerability of people with dementia to abuse and neglect is widely recognised (Compton et al., 1997; Bond et al., 1999; Bonnie & Wallace, 2003), although incidence, prevalence and precise risk factors are not well established (House of Commons Health Committee, 2004a). Practitioners working with people with dementia are required to follow policy and practice guidance (Department of Health/Home Office, 2000) that requires multi-agency responses for the protection of vulnerable adults from abuse. [...] Recommendations from a series of high-profile inquiries into care settings in hospitals (for example, Rowan Ward, Department of Health/Care Services Improvement Partnership, 2005) are relevant to commissioners, regulatory bodies and practitioners in seeking to lower the risk of abuse. The law in this area is developing and the Mental Capacity Act 2005 introduces a new criminal offence of ill treatment or neglect of a person who lacks capacity.” (p. 85) |
| **7. Special situations for decision-making** |  |  |
| Sexual relationships (e.g. prevention of abuse or exploitation) | E | “At the time of diagnosis and when indicated subsequently, the impact of  dementia on relationships, including sexual relationships, should be assessed in a sensitive manner. When indicated, people with dementia and/or their partner and/or carers should be given information about local support services.” (p. 16) |
| Thorough consideration of the indication for genetic testing (e.g. prevention of stigmatization) | I | “If a genetic cause for dementia is not suspected, including late-onset dementia, genotyping should not be undertaken for clinical purposes.” (p. 159) |
| Usage of GPS and other monitoring techniques (e.g. adequately balancing potential benefits, harms, and patient consent) | N | n/a |
| Particularities in drug treatment (e.g. horough consideration before prescribing) | R  +/- | “People with Alzheimer’s disease, vascular dementia or mixed dementias  with mild-to-moderate non-cognitive symptoms should not be prescribed  antipsychotic drugs because of the possible increased risk of cerebrovascular adverse events and death.” (p. 29)  “People with Alzheimer’s disease, vascular dementia, mixed dementias or  DLB with severe non-cognitive symptoms (psychosis and/or agitated  behaviour causing significant distress) may be offered treatment with an  antipsychotic drug after the following conditions have been met. ● There should be a full discussion with the person with dementia and/or carers about the possible benefits and risks of treatment. In particular, cerebrovascular risk factors should be assessed and the possible increased risk of stroke/transient ischaemic attack and possible adverse effects on cognition discussed. ● Changes in cognition should be assessed and recorded at regular intervals. Alternative medication should be considered if necessary. ● Target symptoms should be identified, quantified and documented. ● Changes in target symptoms should be assessed and recorded at regular intervals. ● The effect of comorbid conditions, such as depression, should be considered. ● The choice of antipsychotic should be made after an individual risk–benefit analysis. ● The dose should be low initially and then titrated upwards. ● Treatment should be time limited and regularly reviewed (every 3 months or according to clinical need). For people with DLB, healthcare professionals should monitor carefully for the emergence of severe untoward reactions, particularly neuroleptic sensitivity reactions (which manifest as the development or worsening of severe extrapyramidal features after treatment in the accepted dose range or acute and severe physical deterioration following prescription of antipsychotic  drugs for which there is no other apparent cause).” (p. 29) |
| Covert medication (e.g. balancing short-term medical benefits vs. long-term impact on patient–physician relationship) | E | Ethical issues that are specific to the care of people with dementia, for example, mental capacity, consent, covert medication, restraint and end-of-life issues. (Appendix, p301) |
| Thorough consideration of the indication for brain imaging (e.g. dealing with current lack of evidence for clinical validity of brain imaging in diagnosing dementia) | I | “Systematic reviews have suggested that between 2.2 and 5% of cases with suspected dementia had conditions that required structural neuroimaging to assist with diagnosis (Chui & Zhang, 1997; Clarfield, 2003).  Though such lesions can sometimes be suspected on clinical grounds by factors such as atypical history, early neurological signs, seizure, disturbance and short duration – factors that may prioritise those who undergo imaging if resources are limited (Royal College of Psychiatrists, 2005b) – a systematic review of six different clinical prediction rules for neuroimaging in dementia showed that most had poor sensitivity and all low specificity (Gifford et al., 2000).” (p. 153) |
| Usage of physical restraints (e.g. balancing patient’s best interests with autonomy) | R  +/- | “Health and social care staff who care for people with dementia should  identify, monitor and address environmental, physical health and  psychosocial factors that may increase the likelihood of behaviour that  challenges, especially violence and aggression, and the risk of harm to self  or others. These factors include: ● overcrowding ● lack of privacy  ● lack of activities ● inadequate staff attention ● poor communication between the person with dementia and staff ● conflicts between staff and carers ● weak clinical leadership.” (p. 262-263)  “Health and social care staff should be trained to anticipate behaviour that challenges and how to manage violence, aggression and extreme agitation, including de-escalation techniques and methods of physical restraint.” (p. 30)  “Healthcare professionals who use medication in the management of violence, aggression and extreme agitation in people with dementia should: ● be trained in the correct use of drugs for behavioural control, specifically benzodiazepines and antipsychotics ● be able to assess the risks associated with pharmacological control of violence, aggression and extreme agitation, particularly in people who may be dehydrated or physically ill ● understand the cardiorespiratory effects of the acute administration of benzodiazepines and antipsychotics and the need to titrate dosage to effect ● recognise the importance of nursing people who have received these drugs in the recovery position and of monitoring pulse, blood pressure and respiration.” (p. 30-31) |
| Tube feeding (e.g. adequate decision making in the patient’s best interests) | R  +/+ | “Nasogastric and percutaneous endoscopic gastrostomy tubes would seem to provide a safer way to feed people with severe dementia and dysphagia. However, a review of the evidence in 1999 found no relevant randomised clinical trials comparing tube feeding and oral feeding. On the basis of the available data, the reviewers concluded that the best evidence did not support the use of tube feeding in dementia (Finucane et al., 1999). Ethical commentary, making use of this review, concluded that, although there may be individual cases in which tube feeding is not futile, ‘balancing the risks and benefits leads to the conclusion that [feeding tubes] are seldom warranted for patients in the final stages of dementia’ (Gillick, 2000). More recent research continues to support such views (Sanders, 2004; Alvarez-Fernández et al., 2005). A palliative approach and the use of advance directives decrease reliance on tube feeding (Monteleoni & Clark, 2004). The alternative is to manage dysphagia conservatively, using food thickeners with appropriate posture and feeding techniques. Locally implemented protocols exist but require further evaluation (Summersall & Wight, 2005). More recently, NICE published a guideline on nutritional support that includes recommendations for artificial nutrition and hydration (see NICE guideline no. 32, Nutrition Support in Adults: Oral Nutrition Support, Enteral Tube Feeding and Parenteral Nutrition, www.nice.org.uk/cg032niceguideline).” (p. 102-103) |
| Adequate end of life/palliative care (e.g. prevention of poor care or neglect) | R  +/+ | “ Health and social care professionals working with people with dementia and their carers should adopt a palliative care approach. They should consider physical, psychological, social and spiritual needs to maximise the quality of life of the person with dementia and their family.” (p. 33)  “Palliative care professionals, other health and social care professionals, and commissioners should ensure that people with dementia who are dying have the same access to palliative care services as those without dementia.” (p. 33)  “Primary care teams should ensure that the palliative care needs of people with dementia who are close to death are assessed and that the resulting information is communicated within the team and with other health and social care staff.” (p. 33) |
| Dealing with suicidality (e.g. appropriate estimation of quality of life in suicidal patients with dementia) | N | n/a |

| **SCOTLAND (SIGN 2009)** | | |
| --- | --- | --- |
| DSEI | Rating | Citation example |
| **1. Diagnosis and medical indication** |  |  |
| Adequate consideration of the complexity of diagnosing dementia (e.g. unclear cut off for MCI (mild cognitive impairment)) | R  +/- | “Sufficient information should be gathered to apply the diagnostic criteria discussed in this section. As a person with dementia may not be able to give a fully accurate history a relative or carer should also be interviewed.” (p. 3) |
| Adequate timing of diagnosis (e.g. guiding an appropriate “breaking bad news” process) | R  +/- | “Sufficient information should be gathered to apply the diagnostic criteria discussed in this section. As a person with dementia may not be able to give a fully accurate history a relative or carer should also be interviewed.” (p. 3)  “As a person with dementia may not be able to give a fully accurate history a relative or carer should also be interviewed.” (p. 3)  “Healthcare professionals should be aware that some people with dementia may not wish to know their diagnosis.” (p. 21)  “The wishes of the person with dementia should be upheld at all times. […] Where diagnosis is not disclosed there should be a clear record of the reasons.” (p. 22) |
| Reasonableness of treatment indications (e.g. risk of overestimating benefit of pharmaceutical treatment) | I | “Caregivers should receive comprehensive training on interventions that are effective for people with dementia.” (p. 8) |
| Adequate appreciation of the patient (e.g. adequate consideration of the patient as a person; adequate consideration of existing preferences of the patient) | I | “Healthcare professionals should be aware that many people with dementia can understand their diagnosis, receive information and be involved in decision making.” (p.21)  “Information provision at other stages of the patient’s journey of care is generally more focused on carer needs than that of the patient.” (p. 22) |
| **2. Assessing patient decision-making capacity** |  |  |
| Dealing with varying concepts/understanding of “patient’s capacity” among carers | N | n/a |
| Adequate consideration of setting or content in assessing decision-making capacity (e.g. guidance on how to structure the setting for the capacity assessment; involvement of relatives) | E | “As a person with dementia may not be able to give a fully accurate history a relative or carer should also be interviewed.” (p. 3)  “There is a consensus that both people with dementia and their carers are entitled to receive relevant information. […] Patients and carers should be offered information tailored to the patient's perceived needs. […] Formal permission to disclose the diagnosis to carers should be sought.” (p. 21) |
| Adequate assessment of patient’s decision-making capacity (e.g. inadequate or biased assessment) | N | n/a |
| **3. Information and disclosure** |  |  |
| Adequate consideration of the complexity of informing patients with dementia (e.g. the amount and manner of provision of information) | R  +/+ | “Healthcare professionals should be aware that some people with dementia may not wish to know their diagnosis.” (p. 21)  “The wishes of the person with dementia should be upheld at all times. […] Where diagnosis is not disclosed there should be a clear record of the reasons.” (p. 22) |
| Adequate involvement of relatives in the care process | R  -/+ | “There is a consensus that both people with dementia and their carers are entitled to receive relevant information. […] Patients and carers should be offered information tailored to the patient's perceived needs. […] Formal permission to disclose the diagnosis to carers should be sought.” (p. 21) |
| **4. Decision-making & consent** |  |  |
| Support of patient’s decision-making capacity | I | “Evidence for diagnostic disclosure is inconsistent, limited and at variance with guidance regarding disclosure, with the perspective of people with dementia being largely neglected. It was found to be more common for carers to be routinely given information than patients, regardless of who delivered the information.” (p. 21)  “Healthcare professionals should be aware that many people with dementia can understand their diagnosis, receive information and be involved in decision making.” (p. 21) |
| Responsible surrogate decision-making (e.g. interpretation of advances directives from the perspective of the patient; seeking consent among different surrogates prior to making decisions) | E | “As a person with dementia may not be able to give a fully accurate history a relative or carer should also be interviewed.” (p. 3)  “There is a consensus that both people with dementia and their carers are entitled to receive relevant information. […] Patients and carers should be offered information tailored to the patient's perceived needs. […] Formal permission to disclose the diagnosis to carers should be sought.” (p. 21) |
| Dealing with the need for advanced care planning (e.g. sensibly informing patients and relatives about the types of decision that might need to be made; informing them of tools such as advance directives*)* | N | n/a |
| Adequate consideration of existing advance directives in medical decision making (e.g. guidance on how to include advance directives in medical decision making; recommendations on how to deal with conflicts between content of advance directives and present behavior and expressions in patients with severe dementia). | N | n/a |
| **5. Social and context-dependent aspects** |  |  |
| Caring for relatives (e.g. recognizing needs of relatives; preventing distress and burnout) | N | n/a |
| Caring for clinical personnel and professional carers (e.g. needs of professionals; preventing distress and burn out) | N | n/a |
| Adequate assessment of the patient’s potential to harm a third person (e.g. in decisions on the ability to drive) | N | n/a |
| Responsible handling of costs and allocation of limited resources (e.g. avoiding futile diagnostic and treatment) | N | n/a |
| **6. Care process & process evaluation** |  |  |
| Continuing assessment of potential benefits and harms in individual care strategies | N | n/a |
| Adequate patient empowerment (e.g. environmental modification adapted to the person and the degree of impairment) | R  +/+ | “Methods of disseminating information which may be appropriate for people with dementia and their carers include: ● written information  ● individual education programmes ● group education programmes  ● counseling ● telemedicine service ● communication workshops ● cognitive behaviour therapy (CBT) ● stress management ● combinations of the above. Not everyone benefits from such interventions. Further studies are required to identify confounding factors so that practitioners can provide relevant information tailored to the needs of the person and their carer, to improve understanding and enable more informed decision making at all stages.” (p. 22) |
| Critical self-monitoring of carers (e.g. team supervision; strategies to rethink the care process) | N | n/a |
| Evaluation of abuse and neglect of patients | N | n/a |
| **7. Special situations for decision-making** |  |  |
| Sexual relationships (e.g. prevention of abuse or exploitation) | N | n/a |
| Thorough consideration of the indication for genetic testing (e.g. prevention of stigmatization) | N | n/a |
| Usage of GPS and other monitoring techniques (e.g. adequately balancing potential benefits, harms, and patient consent) | N | n/a |
| Particularities in drug treatment (e.g. horough consideration before prescribing) | N | n/a |
| Covert medication (e.g. balancing short-term medical benefits vs. long-term impact on patient–physician relationship) |  | n/a |
| Thorough consideration of the indication for brain imaging (e.g. dealing with current lack of evidence for clinical validity of brain imaging in diagnosing dementia) | I | “Imaging can be used to detect reversible causes of dementia and to aid in the differential diagnosis of dementia. The choice of imaging technique varies widely, and includes computed tomography (CT), magnetic resonance imaging (MRI), single photon emission controlled tomography (SPECT) and positron emission tomography (PET).” (p. 7) |
| Usage of physical restraints (e.g. balancing patient’s best interests with autonomy) | N | n/a |
| Tube feeding (e.g. adequate decision making in the patient’s best interests) | N | n/a |
| Adequate end of life/palliative care (e.g. prevention of poor care or neglect) | N | n/a |
| Dealing with suicidality (e.g. appropriate estimation of quality of life in suicidal patients with dementia) | N | n/a |

| **SINGAPORE (MINISTRY OF HEALTH 2007)** | | |
| --- | --- | --- |
| DSEI | Rating | Citation example |
| **1. Diagnosis and medical indication** |  |  |
| Adequate consideration of the complexity of diagnosing dementia (e.g. unclear cut off for MCI (mild cognitive impairment)) | I | “In individuals with suspected cognitive impairment, diagnosis can be made using the DSM-IV criteria for dementia with history from a reliable informant.” (p. 12) |
| Adequate timing of diagnosis (e.g. guiding an appropriate “breaking bad news” process) | E | “Although patients generally would like to know the truth about their own medical condition, the rights of those who do not want to know should also be respected. Health care professionals should therefore seek to understand their patients' preferences with respect to the diagnosis of dementia and act appropriately according to their choice.” (p. 51) |
| Reasonableness of treatment indications (e.g. risk of overestimating benefit of pharmaceutical treatment) | R  +/+ | “The decision to initiate costly symptomatic dementia treatment, such as acetylcholinesterase inhibitors or N-methyl Daspartate antagonists, should always be made in consultation with the patient and family after careful consideration of the expected magnitude of benefit, side effects, co-morbidities and costs of treatment.” (p. 33)  “A trial of withdrawal of symptomatic treatment should be considered  when the harm outweighs the benefit, and should be undertaken only  after careful discussion with the patient and caregiver. Examples  include intolerable or serious side effects, and progression of disease  to the severe stages despite optimising treatment. When attempting  withdrawal, it is important to monitor closely for any deterioration so  that therapy can be quickly reinstated to regain the same level of  symptomatic effect.” (p. 35)  “Clinical trials […] consistently demonstrate modest improvement in (1) cognition and global functioning […] (2) activities of daily living and (3) neuropsychiatric symptoms […] It is unclear whether AchEI therapy confers benefit in terms of reducing time to institutionalisation. […] Patients in whom the start of AchEI treatment is delayed may demonstrate slightly reduced benefits […]” (p. 28)  “The decision to initiate costly symptomatic dementia treatment […] should always be made in consultation with the patient and family after careful consideration of the expected magnitude of benefit, side effects, co-morbidities and costs of treatment. (p. 33)  “[…] the reason for considering NPT first and as an enduring endeavour in addressing difficult behaviour is two-fold. First, NPTs […] address the underlying reasons for the behaviour. Second, medications carry adverse side-effects and often mask and suppress the behaviour that actually serves to communicate the need of the person with dementia. (p. 38)  “The general ethical imperative must be to delay, prevent, stabilise dementia, but not to protract life and morbidity artificially in the severe stages of the disease.” (p. 47) |
| Adequate appreciation of the patient (e.g. adequate consideration of the patient as a person; adequate consideration of existing preferences of the patient) | R  +/+ | “Nevertheless, the dignity of the patient should be respected at all stages irrespective of the patient’s functional status. Therapeutic goals and treatment options that are coherent and appropriate to patient’s functional status should be individually tailored and adjusted at different stages of dementia, with an emphasis on quality for life of the patient, as well as the caregiver. The important question to ask for each individual patient is therefore what value of life holds for the patient, and directed at how life is experienced by the patient under the contextual circumstances specific to the patient. It is important to distinguish such evaluations from morally inappropriate social-worth evaluations, which base treatment decisions on an individual’s ability to contribute to society. The  general ethical imperative must be to delay, prevent, stabilize dementia, but not to protract life and morbidity artificially in the severe stages of the disease.” (p. 47) |
| **2. Assessing patient decision-making capacity** |  |  |
| Dealing with varying concepts/understanding of “patient’s capacity” among carers | E | “A diagnosis of dementia per se, however, does not automatically  imply a loss of decision making capacity, which is specific to each patient and to each medical decision. Therefore, those who cannot comprehend complex situations may still possess the capacity to make simple decisions, or to convey their opinions regarding the burdens and benefits of ongoing treatments.” (p. 48) |
| Adequate consideration of setting or content in assessing decision-making capacity (e.g. guidance on how to structure the setting for the capacity assessment; involvement of relatives) | R  +/+ | “In deciding if a patient with dementia possesses adequate capacity with respect to making a particular decision, a clinical evaluation of the following functional abilities should be made: a. Ability to express a choice  b. Ability to understand information provided c. Ability to appreciate significance of information and relevance to self d. Ability to manipulate information rationally before arriving at a decision.” (p. 48) |
| Adequate assessment of patient’s decision-making capacity (e.g. inadequate or biased assessment) | R  +/+ | “Respect for autonomous decision making is a fundamental ethical and legal right of a mentally competent individual. This right of self-determination should be respected to the fullest possible extent, even in dementia or conditions associated with cognitive impairment.” (p. 47)  “When affected by dementia, the key to a patient’s right of autonomy is the presence of adequate decision-making capacity. As a patient’s cognition and hence functional abilities for decision making is impaired in dementia, a patient may or may not possess adequate decision making capacity to make an informed choice.” (p. 47-48)  “If the patient lacks adequate decision making capacity, the ethical imperative switches to one that aims to protect the patient from his or her own harmful decisions or actions. Patients must not be under-treated nor forced to receive inappropriate treatment just because they lack decision making capacity or legally appointed guardian(s). Consideration of the patient’s functional status and quality of life is vital in making treatment  decisions for the patient.” (p. 48) |
| **3. Information and disclosure** |  |  |
| Adequate consideration of the complexity of informing patients with dementia (e.g. the amount and manner of provision of information) | R  +/+ | “Although patients generally would like to know the truth about their own medical condition, the rights of those who do not want to know should also be respected. Health care professionals should therefore seek to understand their patients' preferences with respect to the diagnosis of dementia and act appropriately according to their choice.” (p. 51)  “Studies have shown that the vast majority of patients with mild  dementia wish to be fully informed. Therefore, unless a patient suffering from dementia explicitly declines to be informed of the diagnosis, the default mode should be to inform truthfully as it will enable the patient to: a. plan for optimal life experiences in remaining years of intact  capacities b. designate and appoint a surrogate decision maker to take  over the making of treatment decision upon eventual incompetence  c. settle personal financial and legal matters d. participate in treatment decisions e. consider possible enrolment in research programmes and  participate in informed consent process.” (p. 51) |
| Adequate involvement of relatives in the care process | R  +/- | “When informing the diagnosis, the doctor needs to take into account the patient and family’s prior knowledge and their perception of the problems. The doctor should also be mindful of the impact the diagnosis can have on the patient’s life and family relationships. The communication should therefore be conducted sensitively and empathically, and should include a discussion on treatment options and available support services  both in the hospital and the community.” (p. 51-52)  “After the diagnosis has been communicated, the patient and family should be given time to process the information and to come to terms with it. They should be given ample opportunity to ask questions and seek clarification from the doctor.” (p. 52) |
| **4. Decision-making & consent** |  |  |
| Support of patient’s decision-making capacity | R  +/+ | “Respect for autonomous decision making is a fundamental ethical and legal right of a mentally competent individual. This right of self-determination should be respected to the fullest possible extent, even in dementia or conditions associated with cognitive impairment.” (p. 47)  “In deciding if a patient with dementia possesses adequate capacity with respect to making a particular decision, a clinical evaluation of the following functional abilities should be made: a. Ability to express a choice  b. Ability to understand information provided c. Ability to appreciate significance of information and relevance to self d. Ability to manipulate information rationally before arriving at a decision.” (p. 48)  “When affected by dementia, the key to a patient’s right of autonomy is the presence of adequate decision-making capacity. As a patient’s cognition and hence functional abilities for decision making is impaired in dementia, a patient may or may not possess adequate decision making capacity to make an informed choice.” (p. 47-48)  “If the patient lacks adequate decision making capacity, the ethical imperative switches to one that aims to protect the patient from his or her own harmful decisions or actions. Patients must not be under-treated nor forced to receive inappropriate treatment just because they lack decision making capacity or legally appointed guardian(s). Consideration of the patient’s functional status and quality of life is vital in making treatment  decisions for the patient.” (p. 48) |
| Responsible surrogate decision-making (e.g. interpretation of advances directives from the perspective of the patient; seeking consent among different surrogates prior to making decisions) | R  -/- | “The decision to initiate costly symptomatic dementia treatment, such as acetylcholinesterase inhibitors or N-methyl Daspartate antagonists, should always be made in consultation with the patient and family after careful consideration of the expected magnitude of benefit, side effects, co-morbidities and costs of treatment.” (p. 33) |
| Dealing with the need for advanced care planning (e.g. sensibly informing patients and relatives about the types of decision that might need to be made; informing them of tools such as advance directives*)* | N | n/a |
| Adequate consideration of existing advance directives in medical decision making (e.g. guidance on how to include advance directives in medical decision making; recommendations on how to deal with conflicts between content of advance directives and present behavior and expressions in patients with severe dementia). | N | n/a |
| **5. Social and context-dependent aspects** |  |  |
| Caring for relatives (e.g. recognizing needs of relatives; preventing distress and burnout) | R  +/+ | “Identification of caregiver’s stress can allow targeted family education and counselling at tertiary dementia care centres, or even dementia daycare centres, and this has been shown to reduce institutionalisation.” (p. 16)  “Most patients with dementia are cared for in their own homes by family members. Caregiver management is important for the following reasons:  1) Family caregivers need to be empowered with the necessary knowledge and skills, and psychosocial support to facilitate them  in their task. 2) Family caregivers who face much negative consequences as a result of long-term caregiving need to be helped and supported. Caregiver intervention can take several forms and they include: 1) Education sessions to impart knowledge on dementia and caregiving skills such as communication and behavioural techniques 2) Individual and family counseling 3) Regular caregiver support group meetings 4) Continuous availability of health care professionals and counsellors to provide support and help with crises and the changing nature of the patient’s symptoms  5) Respite care 6) Technology-based interventions” (p. 44) |
| Caring for clinical personnel and professional carers (e.g. needs of professionals; preventing distress and burn out) | I | “If […] the behavioural problems are assessed to be significant causing difficulty in caring process […] with significant amount of caregiver distress, there has to be a discussion with the family members with regards to antipsychotic therapy, with the attendant risks of adverse effects […]” (p. 42) |
| Adequate assessment of the patient’s potential to harm a third person (e.g. in decisions on the ability to drive) | R  +/+ | “Driving also represents independence, freedom and mobility.  The issue of driving in dementia requires therefore the balancing  of individual freedom and patient confidentiality on one side  versus public and patient safety on the other” (p.50) |
| Responsible handling of costs and allocation of limited resources (e.g. avoiding futile diagnostic and treatment) | R  +/+ | “Until definitive findings are available, practitioners should continue to individualise treatment decisions for each patient. For instance, when financial resources are limited, the opportunity cost of employing a maid to look after a patient requiring help with activities of daily living may override the modest benefits of symptomatic therapy.” (p. 33)  “The decision to initiate costly symptomatic dementia treatment […] should always be made in consultation with the patient and family after careful consideration of the expected magnitude of benefit, side effects, co-morbidities and costs of treatment. […] Despite the evidence of efficacy […] there is a debate over whether […] therapy are cost effective, because the treatment effects are small and not always aparent in practice.” (p. 33) |
| **6. Care process & process evaluation** |  |  |
| Continuing assessment of potential benefits and harms in individual care strategies | R  +/- | “A trial of withdrawal of symptomatic treatment should be considered when the harm outweighs the benefit, and should be undertaken only after careful discussion with the patient and caregiver. “ (p. 35)  “In all patients started on antipsychotic medication, they should be monitored carefully for side effects and response to treatment. In patients who are stable, antipsychotic withdrawal should be considered.” (p. 42) |
| Adequate patient empowerment (e.g. environmental modification adapted to the person and the degree of impairment) | E | “As the person with dementia is, by nature of the disease, largely unable to help himself, most interventions are directed at the family caregiver to indirectly help the person with dementia.” (p. 44) |
| Critical self-monitoring of carers (e.g. team supervision; strategies to rethink the care process) | R  +/+ | “Identification of caregiver’s stress can allow targeted family education and counselling at tertiary dementia care centres, or even dementia daycare centres, and this has been shown to reduce institutionalisation.” (p. 16)  “Most patients with dementia are cared for in their own homes by family members. Caregiver management is important for the following reasons:  1) Family caregivers need to be empowered with the necessary knowledge and skills, and psychosocial support to facilitate them  in their task. 2) Family caregivers who face much negative consequences as a result of long-term caregiving need to be helped and supported. Caregiver intervention can take several forms and they include: 1) Education sessions to impart knowledge on dementia and caregiving skills such as communication and behavioural techniques 2) Individual and family counseling 3) Regular caregiver support group meetings 4) Continuous availability of health care professionals and counsellors to provide support and help with crises and the changing nature of the patient’s symptoms  5) Respite care 6) Technology-based interventions” (p. 44) |
| Evaluation of abuse and neglect of patients | E | “Elder abuse may also occur when the frustrated caregiver directs his  or her stress at the vulnerable patient whose care is proving to be difficult. Likewise, financial difficulties should be asked for and if problems are identified, families can be referred to a social worker.” (p. 16) |
| **7. Special situations for decision-making** |  |  |
| Sexual relationships (e.g. prevention of abuse or exploitation) | N | n/a |
| Thorough consideration of the indication for genetic testing (e.g. prevention of stigmatization) | R  +/+ | “As with any medical test, the decision regarding the utility of a genetic test should take into consideration the benefits to the individual patient and the ways that a test result would modify the care that the patient would receive. As in any genetic testing, especially in pre-symptomatic  susceptibility testing, individuals must be clearly informed  regarding: a. potential for severe psychological complications of testing  positive for an incurable, devastating illness b. potential ramifications in the area of employment and medical insurance c. probabilistic implications of a positive test on genetically related family members, who may not have participated in any counselling or consented to testing.” (p. 49, and following) |
| Usage of GPS and other monitoring techniques (e.g. adequately balancing potential benefits, harms, and patient consent) | N | n/a |
| Particularities in drug treatment (e.g. horough consideration before prescribing) | R  +/+ | Antidepressants may be used for the treatment of comorbid  depression in dementia provided their use has been evaluated carefully for each patient […] Conventional and atypical antipsychotics may be used with caution, given their side effect profile, to treat neuropsychiatric symptoms of dementia” (p.4f)  “Conventional antipsychotics have been used to treat behavioural problems associated with dementia. There is evidence for slight benefit of haloperidol over placebo for treatment of aggression. However, adverse events of extrapyramidal side effects and somnolence may limit its routine use.113 Low-potency antipsychotics like chlorpromazine should be used with caution in view of postural hypotension and anticholinergic side-effects.[…]” (p.39) |
| Covert medication (e.g. balancing short-term medical benefits vs. long-term impact on patient–physician relationship) |  | n/a |
| Thorough consideration of the indication for brain imaging (e.g. dealing with current lack of evidence for clinical validity of brain imaging in diagnosing dementia) | R  +/+ | “Whether all patients with dementia require a structural imaging is an  important clinical question, for which there is no consensus. The  value of neuroimaging is in the identification of cerebral infarcts and  clinically important surgical brain lesions (SBLs) such as subdural  haematomas, cerebral tumors and normal pressure hydrocephalus.  The Canadian Consensus Conference on the Assessment of Dementia  (CCCAD)45 (Appendix 6) has outlined the criteria for undertaking a  CT scan of the head, only if certain clinical conditions are met. In a  patient with advanced dementia of a long duration (>2 years based on  CCCAD’s recommendations), we believe a brain scan is not  warranted to detect potentially reversible SBLs. Conversely, if the  patient’s dementia is only mild to moderate (even after 2 years), it is  still advisable to request for an initial CT scan of the brain. If the  clinician is not inclined to perform a brain scan, there is immense  value in discussing the matter with the caregivers and in securing  their agreement not to order a neuroimaging procedure.” (p. 25) |
| Usage of physical restraints (e.g. balancing patient’s best interests with autonomy) | R  +/- | “Restraints, whether environmental, physical or pharmacological, are used in the management of patients with dementia to restrict or control the patient’s movement or behaviour that may compromise the safety of the patient and/or others.” (p. 52)  “However, the use of restraints is not without potential problems:  a. Risk of harm and injury b. Decrease in ability to perform cognitive and physical activities, thereby resulting in cognitive and functional  decline, and ultimately loss of independence c. Loss of freedom, leading to loss of confidence and self-esteem.” (p. 52)  “The preferred choice should therefore be to avoid the use of restraints. Restraints should not be a substitute for a proactive search for reversible precipitating factors for patient’s behavioural problems, or for good communication with the patient.” (p. 52)  “If restraints have to be used on a patient, it should be seen as a temporary means and should be stopped as soon as the indication is no longer present. Excessive use of restraints should be avoided - any restraint should therefore be instituted for the minimal period of time and at the minimal strength or degree needed to achieve the intended outcome for the patient. The patient should also be carefully monitored while on  restraints.” (p. 52) |
| Tube feeding (e.g. adequate decision making in the patient’s best interests) | N | n/a |
| Adequate end of life/palliative care (e.g. prevention of poor care or neglect) | N | n/a |
| Dealing with suicidality (e.g. appropriate estimation of quality of life in suicidal patients with dementia) | N | n/a |

| **CANADA (CMA 2007)** | | |
| --- | --- | --- |
| DSEI | Rating | Citation example |
| **1. Diagnosis and medical indication** |  |  |
| Adequate consideration of the complexity of diagnosing dementia (e.g. unclear cut off for MCI (mild cognitive impairment)) | R  +/- | “Physicians should be aware that most dementias may be proceeded by a  recognizable phase of mild cognitive decline. Physicians should be familiar  with the concept of mild cognitive impairment (MCI) [or cognitive impairment not dementia (CIND)] as a high risk state for decline and dementia.” (p. 3)  “There is fair evidence that physicians should closely monitor individuals who have MCI or CIND, because of the known increased risk of both dementia and death that has been documented.” (p. 3)  “The distinction between MCI and AD is important and is currently made on the basis of clinical assessment of cognition and function.” (p. 6)  “The diagnosis and differential diagnosis of dementia is currently a clinically integrative one. Neuropsychological assessment alone cannot be used for this purpose and should be used selectively in clinical settings. Neuropsychological assessment may aid in: a) Addressing the distinction between normal aging, MCI-CIND and early dementia.” (p. 6) |
| Adequate timing of diagnosis (e.g. guiding an appropriate “breaking bad news” process) | R  -/- | “The process of diagnostic disclosure for persons with cognitive impairment or dementia must begin as soon as the possibility of cognitive impairment is suspected. (Grade A, Level 3) 2. Both the diagnosis of dementia and the disclosure of the diagnosis must be considered processes that provide opportunities for education and discussion.” (p. 23) |
| Reasonableness of treatment indications (e.g. risk of overestimating benefit of pharmaceutical treatment) | R  -/- | “Some persons with dementia may benefit from the following: music; Snoezelen (multi-sensory stimulation); bright light therapy; reminiscence therapy; validation therapy; aroma therapy; and, massage and touch therapy.” (p. 17)  “Non-pharmacological treatment of BPSD should be considered first. Non-pharmacological interventions are often used in combination with  pharmacotherapy.” (p. 17)  “Medications for BPSD should normally be initiated at a low starting dose and then subsequently titrated carefully based on the patient’s response and the presence of adverse effects.” (p. 17) |
| Adequate appreciation of the patient (e.g. adequate consideration of the patient as a person; adequate consideration of existing preferences of the patient) | E | “Once a diagnosis is established, this must be disclosed to the patient and  their family/caregivers in a manner that is consistent with the expressed  wishes of the patient.” (p. 23) |
| **2. Assessing patient decision-making capacity** |  |  |
| Dealing with varying concepts/understanding of “patient’s capacity” among carers | E | “A diagnosis of dementia, or other forms of cognitive impairment, does not preclude competence to provide informed consent, whether it be for treatment decisions, for participation in clinical trials or for participation in non-therapeutic research. Competency must be considered as the ability to make an informed decision about participation in the particular context of the specific treatment or research study.” (p. 23)  “The potential that competency for treatment and research decision-making will change over time must be recognized. This may lead to a change from one of obtaining the patient's ongoing consent to one of obtaining ongoing assent. Assent is almost invariably required, and the decision to discontinue treatment, whether it be therapy or research, must always be an option.” (p. 24) |
| Adequate consideration of setting or content in assessing decision-making capacity (e.g. guidance on how to structure the setting for the capacity assessment; involvement of relatives) | I | “After treatment has been started, patients should be reassessed regularly by the appropriate health care professional involved in their care.” (p. 13) |
| Adequate assessment of patient’s decision-making capacity (e.g. inadequate or biased assessment) | N |  |
| **3. Information and disclosure** |  |  |
| Adequate consideration of the complexity of informing patients with dementia (e.g. the amount and manner of provision of information) | E | “Primary care physicians should be able to communicate appropriate information concerning dementia, including realistic treatment expectations to their patients and their families.” (p. 13) |
| Adequate involvement of relatives in the care process | R  +/- | “The referral/consultation process is essential to the delivery of high quality health care. In the care of a patient with mild to moderate dementia, reasons to consider referral to a geriatrician, geriatric psychiatrist, neurologist, or other health care professional (e.g., neuropsychologist, nurse, nurse practitioner, occupational therapist, physical therapist, psychologist, social worker, other) with the appropriate knowledge and expertise in dementia care would include: [...] Request by the patient or the family for another opinion.” (p. 11)  “Although each case should be considered individually, in general the  diagnosis of dementia should be disclosed to the patient and family.  This process should include a discussion of prognosis, diagnostic  uncertainty, advance planning, driving issues, treatment options,  support groups, and future plans. (p. 12)  “Primary care physicians should be able to communicate appropriate  information concerning dementia, including realistic treatment expectations to their patients and their families. (p. 13)  “The clinician should acknowledge the important role played by the  caregiver in dementia care. The clinician should work with caregivers  and families on an ongoing basis and schedule regular appointments  for patients and caregivers together and alone.” (p. 18) |
| **4. Decision-making & consent** |  |  |
| Support of patient’s decision-making capacity | I | “After treatment has been started, patients should be reassessed regularly by the appropriate health care professional involved in their care.” (p. 13) |
| Responsible surrogate decision-making (e.g. interpretation of advances directives from the perspective of the patient; seeking consent among different surrogates prior to making decisions) | R  -/+ | “To the best of their ability, clinicians and researchers must ensure that the decisions made by proxies regarding treatment and research are based on the prior attitudes and values of the patient. Proxies have a responsibility to represent the patient and all parties must recognize the challenges of doing so.” (p. 24)  “Even in the absence of a legal determination of the competency of the patient with cognitive impairment or dementia, it is important that the clinician and researcher consider the consent process as one that should involve both the patient and their family/caregiver for treatment and research decision-making.” (p. 24) |
| Dealing with the need for advanced care planning (e.g. sensibly informing patients and relatives about the types of decision that might need to be made; informing them of tools such as advance directives*)* | R  -/+ | “Although each case should be considered individually, in general the  diagnosis of dementia should be disclosed to the patient and family.  This process should include a discussion of prognosis, diagnostic uncertainty, advance planning, driving issues, treatment options, support groups, and future plans.” (p. 12)  “While patients with AD retain capacity, they should be encouraged to  update their will and to enact both an advance directive and an enduring power of attorney.” (p. 12) |
| Adequate consideration of existing advance directives in medical decision making (e.g. guidance on how to include advance directives in medical decision making; recommendations on how to deal with conflicts between content of advance directives and present behavior and expressions in patients with severe dementia). | N | n/a |
| **5. Social and context-dependent aspects** |  |  |
| Caring for relatives (e.g. recognizing needs of relatives; preventing distress and burnout) | R  -/+ | “In-home systematic, comprehensive support by a health care provider  with advanced training in dementia care over an extended period (i.e.,  couple of years); In-home psychoeducational intervention that teaches caregivers how to manage behavioral problems.” (p. 17)  “The clinician should: enquire about caregiver information and support  needs; provide education to patients and families about dementia; and,  assist in recruiting other family members and formal community  services to share the caregiving role. If available refer patients to  specialized dementia services (e.g., Alzheimer Society, community based  dementia programs, memory clinics) that offer comprehensive  treatment programs including caregiver support, education, and  training. The clinician should: enquire about caregiver health (both physical and psychiatric); offer treatment for these problems (including individual psychotherapy or medications as indicated); and, refer to appropriate  specialists.” (p. 18) |
| Caring for clinical personnel and professional carers (e.g. needs of professionals; preventing distress and burn out) | N |  |
| Adequate assessment of the patient’s potential to harm a third person (e.g. in decisions on the ability to drive) | E | “In places where comprehensive off and on-road driving evaluations are  not available, clinicians must rely on their own judgment.” (p. 18)  “No single brief cognitive test (e.g., MMSE) or combination of brief  cognitive tests has sufficient sensitivity or specificity to be used as a  sole determinant of driving ability. Abnormalities on cognitive tests  such as the MMSE, clock drawing, and Trails B should result in further  in-depth testing of driving ability.” (p. 18)  “Clinicians should counsel persons with a progressive dementia (and  their families) that giving up driving will be an inevitable consequence  of their disease. Strategies to ease this transition should occur early in  the clinical course of the disease.” (p. 18) |
| Responsible handling of costs and allocation of limited resources (e.g. avoiding futile diagnostic and treatment) | N | n/a |
| **6. Care process & process evaluation** |  |  |
| Continuing assessment of potential benefits and harms in individual care strategies | R  +/+ | “Due to their relative invasiveness and availability of other fairly accurate diagnostic modalities (clinical, neuropsychological, and neuroimaging), CSF biomarkers should not be routinely performed in all subjects undergoing evaluation for memory loss.” (p. 7)  “Careful review of all available documentation (examination, clinical records, autopsy reports, etc.) on reportedly affected relatives is essential to rule out the heterogeneous causes of dementia in the elderly, including depression, alcoholism, vascular dementia, etc. If appropriate after review, genetic counselling should include the risks associated with an autosomal dominant mode of inheritance as the uppermost risk. It is advisable, for the benefit of future generations, to bank DNA and/or autopsy material from affected individuals in such families in case novel gene mutations may be discovered in the future.” (p. 9)  “Medications for the treatment of cognitive and functional manifestations of AD should be discontinued when: a) The patient and/or their proxy decision maker decides to stop; b) The patient refuses to take the medication; c) The patient is sufficiently non-adherent with the medication that continued prescription of it would be useless, and it is not possible to establish a system for the administration of the medication to rectify the problem; d) There is no response to therapy after a reasonable trial; e) The patient experiences intolerable side effects; f) The comorbidities of the patient make continued use of the agent either unacceptably risky or futile (e.g., terminally ill); or, g) The patient’s dementia progresses to a stage where there is no significant benefit from continued therapy.” (p. 14-15) |
| Adequate patient empowerment (e.g. environmental modification adapted to the person and the degree of impairment) | R  -/+ | “There is fair evidence that physicians and therapists should promote  engagement in cognitive activity as part of an overall "healthy lifestyle"  formulation for elderly individuals with and without memory loss.” (p. 3) “There is fair evidence that physicians and therapists should promote physical activity at an intensity level that is adapted to the persons' overall  physical capacities, as part of a ‘healthy lifestyle’ for older individuals with and without memory loss.” (p. 3) |
| Critical self-monitoring of carers (e.g. team supervision; strategies to rethink the care process) | E | “While there is insufficient evidence to make a firm recommendation for the primary prevention of dementia, physicians may advocate for appropriate levels of education and strategies to retain students in appropriate learning environments.” (p. 1)  “After stopping therapy for AD, patients should be carefully monitored and if there is evidence of a significant decline in their cognitive status, functional abilities, or the development/worsening of behavioural challenges, consideration should be given to re-instating the therapy.” (p. 15)  “All clinicians caring for patients with mild to moderate AD have to acquire the core knowledge and skills required to manage this condition.” (p. 19) |
| Evaluation of abuse and neglect of patients | N | n/a |
| **7. Special situations for decision-making** |  |  |
| Sexual relationships (e.g. prevention of abuse or exploitation) | N | n/a |
| Sexual relationships (e.g. prevention of abuse or exploitation) | R  +/+ | “With appropriate pre-and post-testing counselling, predictive genetic testing (PGT) can be offered to “at risk” individuals. […] In young persons (60 years or younger) presenting with an early onset dementia, it is sometimes worthwhile to test for the most common mutations based on the ‘best estimate’ diagnosis. If a mutation is identified, it would have direct implications for offspring of the individual (if a de novo mutation is assumed). Conversely, it would also be important to test other family members such as parents and siblings for possible non-penetrance of a mutation. Careful review of all available documentation (examination, clinical records, autopsy reports, etc.) on reportedly affected relatives is essential to rule out the heterogeneous causes of dementia in the elderly, including depression, alcoholism, vascular dementia, etc. If appropriate after review, genetic counselling should include the risks associated with an autosomal dominant mode of inheritance as the uppermost risk. It is advisable, for the benefit of future generations, to bank DNA and/or autopsy material from affected individuals in such families in case novel gene mutations may be discovered in the future.” (p. 9)  “After careful genetic counselling with family members, if it is decided that genetic testing and/or banking of DNA (or autopsy material) for future studies is in the best interest of family members, this may be done even without the consent or assent of the affected individual who is not  cognitively competent, if consent from the family is given. (Grade B, Level 2) However, extreme care must be taken to minimize any distress the patient may experience while the sample is being obtained. In cases where family members, after extensive counselling, cannot agree on a plan, the case may have to go before a medical ethics committee. Concerned family members should have appropriate pre- and post-test  counselling available to be able to make informed decisions. (Grade B,  Level 2) In cases of conflict among family members, medical ethics  committees may become involved.” (p. 10) |
| Thorough consideration of the indication for genetic testing (e.g. prevention of stigmatization) | N | n/a |
| Usage of GPS and other monitoring techniques (e.g. adequately balancing potential benefits, harms, and patient consent) | N | n/a |
| Particularities in drug treatment (e.g. horough consideration before prescribing) |  |  |
| Covert medication (e.g. balancing short-term medical benefits vs. long-term impact on patient–physician relationship) | I | “1. There is fair evidence that functional imaging with PET or SPECT scanning might assist specialists in the differential diagnosis of dementia, particularly those with questionable early stage dementia or those with frontotemporal dementia. There is variability across centers, with requisite expertise in these modalities that needs to be taken into account in determining utility. (Grade B, Level 2) 2. fMRI and MRS scanning are not recommended for use by family physicians or specialists to make or differentiate a diagnosis of dementia in people presenting with cognitive impairment. They remain very promising research tools. (Grade D, Level 3)” (p. 9) |
| Thorough consideration of the indication for brain imaging (e.g. dealing with current lack of evidence for clinical validity of brain imaging in diagnosing dementia) | N | n/a |
| Usage of physical restraints (e.g. balancing patient’s best interests with autonomy) | N | n/a |
| Tube feeding (e.g. adequate decision making in the patient’s best interests) | N | n/a |
| Adequate end of life/palliative care (e.g. prevention of poor care or neglect) | N | n/a |

| **NEW ZEALAND (NZGG 1998)** | | |
| --- | --- | --- |
| DSEI | Rating | Citation example |
| **1. Diagnosis and medical indication** |  |  |
| Adequate consideration of the complexity of diagnosing dementia (e.g. unclear cut off for MCI (mild cognitive impairment)) | I | “The presence of cognitive loss or deterioration in functioning level, often reported by others. Because patients with dementia are often unaware of their problems, a history from family, carer or neighbour is essential. Reports of change in cognitive function or behaviour by those who know a person well should always be taken seriously. [...] Patients, carers and families should be encouraged to keep regular contact with the general practitioner, particularly if changes occur.” (p. 6) |
| Adequate timing of diagnosis (e.g. guiding an appropriate “breaking bad news” process) | I | “The presence of cognitive loss or deterioration in functioning level, often reported by others. Because patients with dementia are often unaware of their problems, a history from family, carer or neighbour is essential. Reports of change in cognitive function or behaviour by those who know a person well should always be taken seriously. [...] Patients, carers and families should be encouraged to keep regular contact with the general practitioner, particularly if changes occur.” (p. 6) |
| Reasonableness of treatment indications (e.g. risk of overestimating benefit of pharmaceutical treatment) | R  +/+ | “Currently there are no known treatments which delay progression or reverse deterioration for people with the primary dementias. Dementias which are possibly reversible (eg, hypothyroidism, vitamin B12 deficiency, space occupying lesion and depression) have been mentioned elsewhere. A large number of compounds have been examined for possible cognition enhancing effects with mostly disappointing results. “ (p. 6)  “The activity programme appeared to reduce the presence of behaviour disorders in the treatment group as well as reducing the  requirements for psychotropic drug use or restraints.” (p. 12)  “Medication can be very helpful in treating some behavioural problems, but should not be regarded as first-line treatment (except in emergencies). Other strategies should be tried first and continue in parallel with drug treatment. Psychotropic medication will not solve disinhibition or wandering without producing over-sedation; nor will it help negative symptoms or incontinence. The specific goals of treatment should be clear at the outset. The golden rule is to start with low doses and increase slowly, whilst carefully monitoring both beneficial and adverse effects. Dosage times should be tailored to the target problem – behaviour is often most difficult in the latter part of the day. The benefits and risks of treatment should be openly discussed with the patient (if possible)  and carers. In this way unrealistic expectations and fears can be dispelled. Adverse effects are unfortunately very common. These include: sedation, confusion, decreased mobility, low blood pressure and Parkinsonism. It is also important to be alert to the possibility of paradoxical worsening of behaviour. Once instituted, drug treatment should be reviewed on an ongoing basis and attempts made to reduce or withdraw it. Many behavioural problems are relatively short-lived, so psychotropic drugs should not be prescribed indefinitely.” (p. 13) |
| Adequate appreciation of the patient (e.g. adequate consideration of the patient as a person; adequate consideration of existing preferences of the patient) | R  +/- | “Careworkers in rest homes and hospitals should make an effort to familiarise themselves with the person with dementia’s background, habits and  personality. Without this knowledge they are unlikely to be able to understand or deal effectively with challenging behaviours.” (p. 12)  “The issues of care should be sensitively discussed, and health professionals should ensure that their own values do not intrude.” (p. 22) |
| **2. Assessing patient decision-making capacity** |  |  |
| Dealing with varying concepts/understanding of “patient’s capacity” among carers | E | “Dementia does not always affect cognition globally. A person can retain insight in decision making ability in some aspects of their life while otherwise being quite disabled.” (p. 18)  “The issues of care should be sensitively discussed, and health professionals should ensure that their own values do not intrude.” (p. 22) |
| Adequate consideration of setting or content in assessing decision-making capacity (e.g. guidance on how to structure the setting for the capacity assessment; involvement of relatives) | N |  |
| Adequate assessment of patient’s decision-making capacity (e.g. inadequate or biased assessment) | E | “The question of whether a person’s mental state is deteriorating must be judged in context. The primary care physician who has known a person for years and is familiar with his/her background is often in an ideal position to identify dementia.” (p. 5) |
| **3. Information and disclosure** |  |  |
| Adequate consideration of the complexity of informing patients with dementia (e.g. the amount and manner of provision of information) | R  +/- | “Dementia services should be provided in a way that respects cultural values and beliefs. Many disability services, however, are based on essentially monocultural models of habilitation.” (p. 25) |
| Adequate involvement of relatives in the care process | R  +/- | “Once the presence of dementia is established, information and support become crucial to the management of the condition for the medical practitioner, the person with dementia, and the family. Access to support and counseling often minimises carer stress and burden, and assists in the maintenance of social and functional skills of the person with dementia.  Carers need to be able to access information in small, manageable ‘bites’. They need information on: · what the diagnosis is, and its prognosis  · how dementia affects the brain · how this may affect the person’s personality, behaviour and functioning · when and how to ask for help  · what services are available and how to access them · legal and financial matters (eg, enduring power of attorney, operation of bank accounts)  · emotional support systems available including: – support groups  – counseling – social work services · respite care available: – day care – short-term respite – in-home respite (eg, sitter service) – intermittent/rotational care – night care · financial assistance available · how to deal with challenging behaviours and difficult issues such as giving up driving · residential care options and how to access and evaluate these.” (p. 19-20) |
| **4. Decision-making & consent** |  |  |
| Support of patient’s decision-making capacity | I | “The question of whether a person’s mental state is deteriorating must be judged in context. The primary care physician who has known a person for years and is familiar with his/her background is often in an ideal position to identify dementia.” (p. 5) |
| Responsible surrogate decision-making (e.g. interpretation of advances directives from the perspective of the patient; seeking consent among different surrogates prior to making decisions) | I | “Particular areas of ethical difficulty include implications for the family, in particular at what stage the person’s autonomy is impaired by loss of memory and judgement to a point where others have to act as proxy.” (p. 18) |
| Dealing with the need for advanced care planning (e.g. sensibly informing patients and relatives about the types of decision that might need to be made; informing them of tools such as advance directives*)* | R  +/- | “Early diagnosis helps family and carers to make contact with support agencies that will help develop the support strategies and services that will be vital as the condition progresses. Forward planning is aided by  access to accurate information and education.” (p. 5)  “People with dementia often become unable to manage their business, financial or personal affairs. They may be unaware that a problem exists. This makes them at risk from the unscrupulous. Forward planning of legal and business administration together with discussion of treatment decisions are best addressed as soon as diagnosis is confirmed when the person with dementia may still be able to express their views. This may be difficult, however, if the person has impaired insight. Testamentary capacity and enduring Power of Attorney should be considered. A person with early dementia who has retained insight may wish to record their options for management of intercurrent illnesses in the form of an ‘Advanced Directive’.” (p. 18) |
| Adequate consideration of existing advance directives in medical decision making (e.g. guidance on how to include advance directives in medical decision making; recommendations on how to deal with conflicts between content of advance directives and present behavior and expressions in patients with severe dementia). | N | n/a |
| **5. Social and context-dependent aspects** |  |  |
| Caring for relatives (e.g. recognizing needs of relatives; preventing distress and burnout) | R  +/+ | “People with Alzheimer’s Disease and other dementias and their families require ongoing counselling from their primary care doctor or appropriate professional when the diagnosis is first suspected and continuing as the reality of progressive dementia and increased dependence touches the family. Families who choose to provide care for patients throughout the course of the illness require access to support groups, good clinical information about the probable course of the disease, effective management strategies, and non-judgmental recommendations as to when to seek outside help and a move to continuing care.” (p. 5)  “Dementia carers have been shown to have poorer physical health status and impaired immune function, as well as higher levels of emotional distress, compared to equivalent samples of carers for other disabled groups. A substantial minority suffer from psychiatric illness, especially depression. There is minimal data on strain among professional carers. It should be noted that caring for a person with dementia, whilst often stressful, may also be a positive, life-enhancing experience.” (p. 13)  “The relationship between support levels and strain is highly complex and variable. Informal (unpaid) support appears to be protective in some circumstances but scientifically sound evidence for any protective benefit of formal (paid) support services is lacking. There is, however, a great deal of descriptive and anecdotal data indicating support services are helpful in many ways to carers and people with dementia. [...] Training programmes for carers have been shown both to relieve strain and to delay institutional placement, and are therefore cost-effective. Key elements in such programmes are: education about the dementing illness, together with its prognosis and possible complications; encouraging the carer to come to terms with losses involved, through expression of feelings; encouraging a more objective caregiving style; and coaching specific  practical strategies.” (p. 14)  “Several factors should be considered when evaluating the strengths of a caregiving relationship and the degree of burden likely to be experienced.” (p. 20; see also Diagram 1, p. 25)  “The stress associated with caring for a person with dementia should never be underestimated. It places an extraordinary burden on those who undertake the caring role. Some people find themselves unwittingly and unwillingly in the role of carer. Other family members may look to one member of the family to take on this role without considering whether this person has the desire, ability or emotional capacity and physical health to cope. For some families, geographical location may place responsibility for care on one member only.” (p. 20) |
| Caring for clinical personnel and professional carers (e.g. needs of professionals; preventing distress and burn out) | R +/+ | “Dementia carers have been shown to have poorer physical health status and impaired immune function, as well as higher levels of emotional distress, compared to equivalent samples of carers for other disabled groups. A substantial minority suffer from psychiatric illness, especially depression. There is minimal data on strain among professional carers. It should be noted that caring for a person with dementia, whilst often stressful, may also be a positive, life-enhancing experience.” (p. 13)  “The relationship between support levels and strain is highly complex and variable. Informal (unpaid) support appears to be protective in some circumstances but scientifically sound evidence for any protective benefit of formal (paid) support services is lacking. There is, however, a great deal of descriptive and anecdotal data indicating support services are helpful in many ways to carers and people with dementia. [...] Training programmes for carers have been shown both to relieve strain and to delay institutional placement, and are therefore cost-effective. Key elements in such programmes are: education about the dementing illness, together with its prognosis and possible complications; encouraging the carer to come to terms with losses involved, through expression of feelings; encouraging a more objective caregiving style; and coaching specific  practical strategies.” (p. 14) |
| Adequate assessment of the patient’s potential to harm a third person (e.g. in decisions on the ability to drive) | R  +/- | „The first priority is to assess whether there is any evidence of danger to the person with dementia or to others. Accidents in the home (e.g., stove, appliances, open fires), assaultive behaviour, impaired driving, malnutrition, suicide threats or apparent abuse or neglect may require urgent action.“ (p. 9)  “Under Section 45A of the Vehicle and Driver Registration and Licensing Act 1976, doctors and optometrists have a legal obligation to notify the Secretary of Transport if a person is not physically or mentally fit to drive. The practitioner, in making the notification, must be satisfied not only about the person’s medical unfitness to drive, but also that the licence holder will probably continue to drive despite medical advice. Information should also be sought from the caregiver about the person’s continuing ability to drive safely. Many small incidents can illustrate deteriorating ability long before a serious or life endangering accident occurs. The role of the medical practitioner in encouraging the person to give up driving cannot be understated. This task must not be left to caregivers or families. Medical practitioners may find this difficult involving as it does a loss of independence for someone who may have been a patient for many years. The discussion may provoke anger on the part of the patient. Some may feel that there is an ethical dilemma at stake. This should be seen, however, as a situation where the safety of others outweighs the rights of the individual.” (p. 17) |
| Responsible handling of costs and allocation of limited resources (e.g. avoiding futile diagnostic and treatment) | N | n/a |
| **6. Care process & process evaluation** |  |  |
| Continuing assessment of potential benefits and harms in individual care strategies | N | n/a |
| Adequate patient empowerment (e.g. environmental modification adapted to the person and the degree of impairment) | R  -/- | “It is important to focus on the remaining strengths, skills and resources of people with dementia, and work toward the maintenance of these. Regular review and care planning, with referral on to counselling support groups or other support agencies, is vital. There is evidence that cognitive decline in dementia may be delayed by participation in stimulating intellectual activities (the ‘use it or lose it’ theory). People with dementia should be encouraged, as far as is possible, to maintain their customary hobbies and activities.” (p. 9) |
| Critical self-monitoring of carers (e.g. team supervision; strategies to rethink the care process) | R  +/- | “Special considerations pertain (in addition to the above) to managing dementia in a secondary care setting. Dementia patients are commonly found in medical and surgical wards but are often poorly cared for. Problems may include: inadequate initial assessment (failure to seek ancillary history); acute confusion mistaken for  dementia; failure to recognise the disorienting effect of hospital environment (undue haste, noise, bed shifting etc); failure to appreciate non-specific presentation of illness or the complexities of multiple interacting medical and psychiatric problems; resentment of failure to cope as a reason for admission; ward culture which fails to tolerate muddled/disturbed behaviour (especially at night). The outcomes of such poor management may include: acute confusion and/or behavioural problems precipitated or exacerbated; iatrogenic problems from inappropriate use of sedatives or restraints; failure to provide adequate social assessment/intervention; admission unnecessarily prolonged (or premature discharge). These can often be prevented by thorough multi-disciplinary assessment/reviews and close liaison with geriatric medicine/psychiatry.  As far as possible staff should strive to improve ward environments and routines to suit dementia patients and to promote ready access to family and friends.” (p. 10)  “Careworkers in rest homes and hospitals should make an effort to familiarise themselves with the person with dementia’s background, habits and  personality. Without this knowledge they are unlikely to be able to understand or deal effectively with challenging behaviours.” (p. 12)  “The issues of care should be sensitively discussed, and health professionals should ensure that their own values do not intrude.” (S. 22)  “Awareness is the key to early diagnosis, support and better management of dementia. As drug treatments become more readily available early detection of dementia will be vital for these to have maximum benefit. [...] Implementation of standardised, mandatory training in dementia for all nursing staff and allied health professionals at undergraduate level. Training of ancillary staff should also reflect this standard.” (p. 24) |
| Evaluation of abuse and neglect of patients | E | “The first priority is to assess whether there is any evidence of danger to the person with dementia or to others. Accidents in the home (e.g., stove, appliances, open fires), assaultive behaviour, impaired driving, malnutrition, suicide threats or apparent abuse or neglect may require urgent action.” (p. 9) |
| **7. Special situations for decision-making** |  |  |
| Sexual relationships (e.g. prevention of abuse or exploitation) | I | “People with dementia are at increased risk of abuse. This can be physical, psychological, financial or sexual. The stresses associated with caring for someone with dementia can tax the resources of even the most patient and this can lead to physical or psychological abuse. This can occur more readily if the carer is unaware of supports.” (p. 19) |
| Thorough consideration of the indication for genetic testing (e.g. prevention of stigmatization) | I | “Over the past five years it has become increasingly apparent that Alzheimer’s Disease is, in great part, a genetic condition. As research progresses there will be a growing need to offer appropriate support to worried relatives, particularly family members of early-onset and/or familial cases.” (p. 10) |
| Usage of GPS and other monitoring techniques (e.g. adequately balancing potential benefits, harms, and patient consent) | N | n/a |
| Particularities in drug treatment (e.g. horough consideration before prescribing) | N | n/a |
| Covert medication (e.g. balancing short-term medical benefits vs. long-term impact on patient–physician relationship) |  |  |
| Thorough consideration of the indication for brain imaging (e.g. dealing with current lack of evidence for clinical validity of brain imaging in diagnosing dementia) | N | n/a |
| Usage of physical restraints (e.g. balancing patient’s best interests with autonomy) | E | “The role of physical restraints (bedrails, chair-belts etc) for managing severe agitation or aggression is controversial. These should only be used as a last resort, in the interests of safety; the indications for, and amount of use should be accurately documented and regularly audited.” (p. 12) |
| Tube feeding (e.g. adequate decision making in the patient’s best interests) | N | n/a |
| Adequate end of life/palliative care (e.g. prevention of poor care or neglect) | E | “Educational interventions must emphasise the need to plan ahead and to address ‘end of life’ decisions.” (p. 18) |
| Dealing with suicidality (e.g. appropriate estimation of quality of life in suicidal patients with dementia) | N | n/a |

| **AUSTRALIA (RACGP 2003)** | | |
| --- | --- | --- |
| DSEI | Rating | Citation example |
| **1. Diagnosis and medical indication** |  |  |
| Adequate consideration of the complexity of diagnosing dementia (e.g. unclear cut off for MCI (mild cognitive impairment)) | E | “The differential diagnosis should include the four ‘D’s of geriatric practice – dementia, delirium, depression and drugs. Remember that the patient’s age, level of education, cultural background and co-morbid illnesses may affect their assessment.” (p. 14) |
| Adequate timing of diagnosis (e.g. guiding an appropriate “breaking bad news” process) | I | “History and functional assessment. This should include: ● full clinical history ● interviews with patient and family, conducted together and separately ● ability to undertake daily activities (dressing,  washing, managing finances, telephone).” (p. 3)  “A full clinical history should be taken. This should include interviews with the patient and their family or carer conducted together and separately. (p. 11) |
| Reasonableness of treatment indications (e.g. risk of overestimating benefit of pharmaceutical treatment) | E | “Medication can be very helpful in treating some behavioural problems, but should not be regarded as first-line treatment (except in emergencies). Other strategies should be tried first and continue  in parallel with drug treatment.” (p. 42)  “There are currently no drugs proven to prevent dementia or modify the neuropathology of the disease once established. However clinical studies have shown that acetycholinesterase inhibitors can improve cognitive function and/or delay or lessen the rate of cognitive and functional decline in patients with mild to moderately severe Alzheimer’s disease. A number of etycholinesterase inhibitors are currently available under the Pharmaceutical Benefits Scheme, provided the patient meets the guidelines (see the PBS Handbook for current guidelines and arrangements). Evidence of benefit is now accumulating for Lewy-body dementia, but not for other types of dementia, including vascular dementia.” (p. 21) |
| Adequate appreciation of the patient (e.g. adequate consideration of the patient as a person; adequate consideration of existing preferences of the patient) | E | “Institutionalisation offers the best duration of survival for people with dementia, survival in this context meaning time until death rather than quality of life. However most patients would prefer to remain living in the community, and usually their carer agrees. A patient should not be assessed for optimal home care independently of the carer, and often both patient and carer prefer a formal care package while remaining at home.” (p. 25)  “If the patient does not have the capacity to consent, then the decision MUST be made by someone other than the treating team members.” (p. 45) |
| **2. Assessing patient decision-making capacity** |  |  |
| Dealing with varying concepts/understanding of “patient’s capacity” among carers | E | “Capacity is task-specific, and must be assessed separately for each decision.” (p. 44) |
| Adequate consideration of setting or content in assessing decision-making capacity (e.g. guidance on how to structure the setting for the capacity assessment; involvement of relatives) | E | “Such a situation raises many issues and emphasises the importance of a home visit. One or more home visits by a general practitioner and/or other members of the team will be needed before assessment is complete. This will usually result in additional history prompted by the situation, better assessment of functioning, sometimes a better environment for cognitive testing, and appreciation of the safety and quality of the environment.” (p. 12) |
| Adequate assessment of patient’s decision-making capacity (e.g. inadequate or biased assessment) | E | “Limitations in the interpretation of these tests include:● other issues that may impair performance such as the presence of dysphasia, sight impairment, deafness, poor educational level, cultural factors, an awareness of the fact that the patient is being tested and fear of testing ● factors that may overcome creased cognition such as better intellect and education.” (p. 12)  “The patient’s capacity to make decisions about matters such as consent to treatment, living circumstances and financial arrangements needs to be determined.” (p. 24) |
| **3. Information and disclosure** |  |  |
| Adequate consideration of the complexity of informing patients with dementia (e.g. the amount and manner of provision of information) | R  +/+ | “Before imparting information, it is important to find out what the patient and family already know about dementia, to reinforce what is correct,  and to correct what is not.” (p. 19)  “Be careful not to overload people with too much information at one time.” (p. 20)  “Listed below are ways to help minimise the distress that breaking the news of dementia may cause: ● Allow adequate time and ensure privacy.  ● Let the patient decide how much they want to know. ● Tell the patient and carer separately. ● Be empathetic and encourage expressions  of feelings. ● Break the news in stages over several consultations.  ● Assess patient’s understanding frequently. ● Be aware that both patients and carers may suffer reactive depression or anxiety after  hearing the diagnosis. ● It is perfectly acceptable to refer the patient  to a specialist to hear the diagnosis if you feel that passing on the diagnosis will damage your relationship with the patient and/or family.” (p. 20)  “Early diagnosis is important because much can be done for the patient at this stage to improve lifestyle and reduce risks, and for carer and family by providing information and support.” (p. 31) |
| Adequate involvement of relatives in the care process | E | “Patient, carer and family need to know what to expect, and the distress of the diagnosis needs to be handled sensitively.” (p. 4)  „What to tell. This depends on what the patient and family need and want to know, but consider: ● what the diagnosis is, and its prognosis ● how this may affect the person’s personality, behaviour and functioning  ● when and how to ask for help ● what services are available and how to access them ● legal and financial matters, eg enduring power of attorney, operation of bank accounts ● emotional support systems available ● support and respite care available ● financial assistance available ● how to deal with challenging behaviours and difficult issues such as giving up driving ● residential care options and how to access and evaluate these ● Enduring Power of Attorney or Guardianship ● making of will. Be careful not to overload people with too much information at one time. Encourage all involved to read the excellent resources available from Alzheimer’s Australia.” (p. 19-20) |
| **4. Decision-making & consent** |  |  |
| Support of patient’s decision-making capacity | E | “Limitations in the interpretation of these tests include: ● other issues that may impair performance such as the presence of dysphasia, sight impairment, deafness, poor educational level, cultural factors, an awareness of the fact that the patient is being tested and fear of testing ● factors that may overcome creased cognition such as better intellect and education.” (p. 12)  “Such a situation raises many issues and emphasises the importance of a home visit. One or more home visits by a general practitioner and/or other members of the team will be needed before assessment is complete. This will usually result in additional history prompted by the situation, better assessment of functioning, sometimes a better environment for cognitive testing, and appreciation of the safety and quality of the environment.” (p. 12)  “The patient’s capacity to make decisions about matters such as consent to treatment, living circumstances and financial arrangements needs to be determined.” (p. 24) |
| Responsible surrogate decision-making (e.g. interpretation of advances directives from the perspective of the patient; seeking consent among different surrogates prior to making decisions) | I | “Carers’ stress can be worsened if other family members or close friends have differing views about management. Such difficulties should be  sought out and dealt with tactfully.” (p. 25)  “Once the question of dementia arises assessment cannot be achieved in one consultation and a plan needs to be agreed with patient and family or carers, together and separately. The Enhanced Primary Care health assessment or care planning items in the Medical Benefits Schedule can be used. At the initial consultation it is necessary to: ● determine the problems ● sort out priorities with patient and family ● manage urgent problems ● deal with the priority problems ● arrange a plan for further assessment and management. This will be encouraged by a focus on dealing with the patient and family’s perceived problems, with follow-up to see that the desired goals are achieved.” (p. 19) |
| Dealing with the need for advanced care planning (e.g. sensibly informing patients and relatives about the types of decision that might need to be made; informing them of tools such as advance directives*)* | R  -/- | “Assessment and management need at least several consultations over weeks or months, and probably a plan for some years, arranged with patient and family. The Enhanced Primary Care health assessment or care planning items in the Medical Benefits Schedule can be used.” (p. 4)  “A management plan should be drawn up with the patient and family, taking into consideration the following issues: ● Initial stage ● Long-term plan ● Follow-up. This should include regular consultations as well  as allowing for extra consultations when necessary.” (p. 5)  “While the patient, carer and family have a right not to be informed of the diagnosis, where possible it is best to inform them so that they  will know what to expect and can begin making any necessary arrangements, such as altering the home environment, changing wills and contacting Alzheimer’s Australia. (p. 19)  “Determination of a patient’s capacity to make decisions may be an important role of the doctor. This may apply in one of three situations: ● consent for medical treatment ● giving an advance care directive ● making a will. It may also apply to other tasks such as managing financial affairs or arranging living circumstances.” (p. 44)  “Forward planning of legal and business administration together with discussion of treatment decisions are best addressed as soon as diagnosis is confirmed when the person with dementia may still be able to express their views. Testamentary capacity, Enduring Power of Attorney or Enduring Guardianship, and advanced care directives should be considered.” (p. 44)  “People with dementia often become unable to manage their business, financial or personal affairs. They may be unaware that a problem exists.  This makes them at risk from the unscrupulous. Forward planning of legal and business administration together with discussion of treatment decisions are best addressed as soon as diagnosis is confirmed when the person with dementia may still be able to express their views.” (p. 44) |
| Adequate consideration of existing advance directives in medical decision making (e.g. guidance on how to include advance directives in medical decision making; recommendations on how to deal with conflicts between content of advance directives and present behavior and expressions in patients with severe dementia). | N | n/a |
| **5. Social and context-dependent aspects** |  |  |
| Caring for relatives (e.g. recognizing needs of relatives; preventing distress and burnout) | R  +/- | “The stress associated with caring for a person with dementia should never be underestimated. It places an extraordinary burden on those who  undertake the caring role. Carers are often elderly, or stressed by other family responsibilities. Higher levels of depression, psychological  morbidity and use of psychotropic medications are seen in carers of those with dementia.” (p. 18)  “Ask the carer ‘How is this affecting you? What has changed for you?’ Ask about the carer’s mood level. Note any changes in the carer’s health  which could be stress related. The Caregiver Burden Scale may be useful.” (p. 19)  “Patients sometimes make life very difficult for their carers. [...] GPs need to be vigilant about the health of the carer as well as the patient with Alzheimer’s disease even if the carer is not their patient.” (p. 25)  “Difficulties experienced with caring can be enough to produce sufficient stress to place either the person with dementia or the carer at risk, or jeopardise the success of community care.” (p. 18)  “Further support for carers can be obtained via the Commonwealth Carer Resource Centre, which has a Carer Information Pack Support Kit that provides information about the support and services available to carers, and offers practical assistance.” (p. 24)  “Several factors should be considered when evaluating the strengths of a caregiving relationship and the degree of burden likely to be experienced. The ability to cope with caring depends on the: ● symptoms exhibited by the person with dementia ● type, frequency and disruptive effects of aberrant behaviour ● duration and severity of the dementia symptoms ● carer’s response to these symptoms and tolerance of aberrant behaviour ● formal and informal support services available to assist ● carer’s emotional and physical health ● carer’s perception of whether they have sufficient emotional support ● quality of the carer’s relationship with the person with dementia prior to the onset of dementia ● carer’s ability to make lifestyle adjustments ● carer’s ability to take over responsibilities and decision-making within the home ● carer’s other commitments.” (p. 39) |
| Caring for clinical personnel and professional carers (e.g. needs of professionals; preventing distress and burn out) | N |  |
| Adequate assessment of the patient’s potential to harm a third person (e.g. in decisions on the ability to drive) | R +/+ | „An early priority is to assess whether there is any evidence of danger to the person with dementia or to others. Falls, accident risks in the home (eg stove, appliances, open fires), impaired driving, malnutrition, suicide threats or apparent abuse or neglect may require urgent action.“ (p. 24)  “Advice from medical practitioners is often heeded by older patients in relation to their ability to drive. Using resources such as the Austroads publication Assessing fitness to drive will aid the general practitioner in making an informed decision in relation to this (Appendix F2).“ (p. 39) |
| Responsible handling of costs and allocation of limited resources (e.g. avoiding futile diagnostic and treatment) | N | n/a |
| **6. Care process & process evaluation** |  |  |
| Continuing assessment of potential benefits and harms in individual care strategies | I | “People with dementia living alone will usually need support. There are complex ethical issues involved in ensuring that a person’s wish to continue living alone is balanced with their safety and that of others.” (p. 24) |
| Continuing assessment of potential benefits and harms in individual care strategies | R  -/- | “Some general practical strategies which carers can adopt: ● Establish a simple, regular routine that suits the person with dementia. ● Establish a physical environment that suits the person with dementia (safe, comfortable, familiar, interesting).” (p. 22)  “An important principle in minimising the difficulties that dementia will cause is to change the environment, not the person: ● Install a whiteboard near the telephone to write messages on. ● Display clocks prominently. ● Use calenders where the current date is obvious. ● Remove loose rugs and low furniture which may cause falls. ● Provide the patient with frequent reminders, explanations and orientation cues.” (p. 22)  “Prevention of falls requires recognition and alteration of environmental risks, modification of risk behaviours, and appropriate physical assistance.” (p. 23)  “‘Use it or lose it’ applies to physical as well as mental activity. Patients need to be encouraged to maintain physical activity appropriate to their  interests and physical state, and this needs to be built into their routine.” (p. 23) |
| Adequate patient empowerment (e.g. environmental modification adapted to the person and the degree of impairment) | R  -/- | Some general practical strategies which carers can adopt: ● Establish a simple, regular routine that suits the person with dementia. ● Establish a physical environment that suits the person with dementia (safe, comfortable, familiar, interesting). ● Be prepared for change, understand that dementia is due to a disorder/disease of the brain and that the affected person has reduced ability to control/think/act. ● Ignore unwanted behaviour or walk away; positive reinforcement of adaptive behaviour.● Expect inconsistencies – patient can sometimes do things, sometimes not (like faulty wiring). ● Distract – try to focus attention away from what is upsetting the person with dementia. ● Use empathy and humour to defuse tension. ● Maintain respect, avoid infantilisation, don’t say to the person ‘I just told you that’. ● Slow pace, avoid rush. ● Give repeated explanation and reassurance. ● Use clear, direct, short and simple communication; importance of eye contact, gestures and appropriate touch. ● Break tasks down into small steps. ● Look at activities in terms of the steps required to perform them.The person may be able to  do some but not all of these eg get dressed, if clothes are selected and put out by someone else. ● If resistance encountered with task, try again later. ● Tolerate the behaviour (avoid arguing or scolding). ● Ensure consistency and avoid change wherever possible.” (p. 22)  “Education and Support of Carers – a good understanding of dementia by carers will assist in better management and lessen breakdown in relationships.” (p. 29) |
| Critical self-monitoring of carers (e.g. team supervision; strategies to rethink the care process) | E | “An early priority is to assess whether there is any evidence of danger to the person with dementia or to others. Falls, accident risks in the home (eg stove, appliances, open fires), impaired driving, malnutrition, suicide threats or apparent abuse or neglect may require urgent action.” (p. 24)  “Recognition of abuse may be difficult and requires awareness of the possibility and tactful inquiring about the stresses of caring. Abuse can be physical, psychological, financial or sexual. The person with dementia can sometimes be the abuser.” (p. 25)  “People with dementia are at increased risk of abuse. This can be physical, psychological, financial or sexual. The stresses associated with caring for someone with dementia can tax the resources of even the most patient person and this can lead to physical or psychological abuse. This can occur more readily if the carer is unaware of supports. It should also be recognised that the person with dementia can sometimes be the abuser.  Recognition of situations where abuse is occurring may be difficult and requires awareness of the possibility by health professionals and tactful  inquiring about the stresses of caring. The prospect of financial gain may sometimes be a source of abuse. As one GP said ‘One of the saddest things is watching the family come in like a pack of sharks for the will’.” (p. 48) |
| **7. Special situations for decision-making** |  |  |
| Sexual relationships (e.g. prevention of abuse or exploitation) | I | “Recognition of abuse may be difficult and requires awareness of the possibility and tactful inquiring about the stresses of caring. Abuse can be physical, psychological, financial or sexual. The person with  dementia can sometimes be the abuser (see p48).” (p. 25) |
| Thorough consideration of the indication for genetic testing (e.g. prevention of stigmatization) | N | n/a |
| Usage of GPS and other monitoring techniques (e.g. adequately balancing potential benefits, harms, and patient consent) | N | n/a |
| Particularities in drug treatment (e.g. horough consideration before prescribing) |  |  |
| Covert medication (e.g. balancing short-term medical benefits vs. long-term impact on patient–physician relationship) | E | “A high proportion of people with dementia of the Lewy-body type are sensitive to neuroleptic agents, and appreciable number of these experience a severe reaction. Delusions or misidentifications are associated with a high number of aggressive episodes. [...] Patients with dementia of the Lewy-body type should not be treated with neuroleptics. // Other neuroleptics include pericyazine, loxapine, thiothizene and pimozide. However these drugs are not recommended for treatment of Lewy-body dementia. In addition, a prospective study has suggested that neuroleptic drugs may hasten cognitive decline in dementia. Whilst further studies are needed to confirm this finding, it emphasises the need for caution and judgment in the use of these drugs.” (p. 30) |
| Thorough consideration of the indication for brain imaging (e.g. dealing with current lack of evidence for clinical validity of brain imaging in diagnosing dementia) | N | n/a |
| Usage of physical restraints (e.g. balancing patient’s best interests with autonomy) | N | n/a |
| Tube feeding (e.g. adequate decision making in the patient’s best interests) | N | n/a |
| Adequate end of life/palliative care (e.g. prevention of poor care or neglect) | N | n/a |
| Dealing with suicidality (e.g. appropriate estimation of quality of life in suicidal patients with dementia) | N |  |

| **FRANCE (HAS 2008)** | | |
| --- | --- | --- |
| DSEI | Rating | Citation example |
| **1. Diagnosis and medical indication** |  |  |
| Adequate consideration of the complexity of diagnosing dementia (e.g. unclear cut off for MCI (mild cognitive impairment)) | I | “Patients who have not lost their autonomy are not described as having dementia but are described as suffering from mild cognitive disorders.” (p. 5)  “Practitioners are advised to conduct an interview with the patient and, with his/her consent if possible, with a named carer who can give reliable information. The interview should cover individual and family medical history, prior and current treatments, level of education, career, the  history of the condition, changes in behaviour, and the impact of the disorders on daily activities.” (p. 5)  “Patients with mild cognitive disorders form part of an at-risk population and should be regularly monitored after having been informed that this will be done.” (p. 11) |
| Adequate timing of diagnosis (e.g. guiding an appropriate “breaking bad news” process) | I | “Early diagnosis of Alzheimer's disease is only advisable if it is certain that care will be provided. Early introduction of treatment, of medical and social care, and of support improves the quality of life of patients and carers for a longer period and could enable patients to live independently for longer. Early diagnosis allows patients and their families to learn more about the condition at a time when patients have not yet developed many symptoms, are still able to communicate with their friends and relatives, any may be able to take decisions as to their subsequent care. It also helps prevent relatives becoming overburdened as the necessary help and support can be introduced at an early stage and increased as required.” (p. 10) |
| Reasonableness of treatment indications (e.g. risk of overestimating benefit of pharmaceutical treatment) | E | “Provided that an investigation into a possible intercurrent cause has taken place and treatment has been given, appropriate non-drug treatment must always be offered as a first step to patients suffering from agitation or shouting, in order to avoid the use of antipsychotics or other sedatives. Such treatment can replace drug therapy.” (p. 14)  “Drugs must be prescribed for a short period and at the lowest effective dose. Treatment should be reassessed at very regular intervals in the light of the patient's clinical condition (at least once every two weeks).” (p. 15) |
| Adequate appreciation of the patient (e.g. adequate consideration of the patient as a person; adequate consideration of existing preferences of the patient) | I | “Practitioners are advised to conduct an interview with the patient and, with his/her consent if possible, with a named carer who can give reliable information. The interview should cover individual and family medical history, prior and current treatments, level of education, career, the history of the condition, changes in behaviour, and the impact of the disorders on daily activities.” (p. 5)  “The interview should investigate: • the type of disorder and its origin; • any use of medicines, alcohol, or poisons that might induce or aggravate cognitive disorders; • previous confusion-related syndromes. It should explore the patient's living conditions (marital status, social and family environment, type of housing, domestic help, etc.) as these will affect his/her care.” (p. 6) |
| **2. Assessing patient decision-making capacity** |  |  |
| Dealing with varying concepts/understanding of “patient’s capacity” among carers | I | “Patients with dementia do not necessarily suffer severe loss of autonomy at the onset of their disease or during the first few years.” (p. 5) |
| Adequate consideration of setting or content in assessing decision-making capacity (e.g. guidance on how to structure the setting for the capacity assessment; involvement of relatives) | I | “Alzheimer's disease should ideally be diagnosed as soon as the first symptoms appear. The diagnosis must involve a detailed cognitive assessment, preferably carried out during a specialised memory consultation.” (p. 5) |
| Adequate assessment of patient’s decision-making capacity (e.g. inadequate or biased assessment) | N | n/a |
| **3. Information and disclosure** |  |  |
| Adequate consideration of the complexity of informing patients with dementia (e.g. the amount and manner of provision of information) | R  -/+ | “Practitioners are advised to disclose the diagnosis of dementia to the patient, in several steps and several times, if necessary. The patient's family or anyone the patient chooses may be informed of the diagnosis with the patient’s consent. During the early stages of the disease, the individual's cognitive abilities are adequate to allow understanding. Disclosure should take account of the patient's background, perception of the condition, and fears, and should be made by the doctor who diagnosed the disease. The patient’s regular doctor and relatives may have to be consulted to prepare the ground. Coordination with the patient's regular doctor is needed; he or she will be responsible for drawing up a care protocol within the French chronic conditions scheme. Disclosure of the diagnosis, even late, may be worthwhile in order to adjust the patient's treatment. Relatives may find such disclosure beneficial as they can then put their past and present experience into words. On late disclosure, care must be taken to listen carefully to the patient, to choose appropriate language, and to take account of the patient’s ability to communicate.” (p. 11) |
| Adequate involvement of relatives in the care process | R  -/- | “According to French law, ‘all individuals are entitled to information about their state of health’. Practitioners are advised to disclose the diagnosis of dementia to the patient, in several steps and several times, if necessary. The patient's family or anyone the patient chooses may be informed of the diagnosis with the patient’s consent. During the early stages of the disease, the individual's cognitive abilities are adequate to allow understanding.” (p. 11)  “Disclosure of the diagnosis, even late, may be worthwhile in order to adjust the patient's treatment. Relatives may find such disclosure beneficial as they can then put their past and present experience into words. On late disclosure, care must be taken to listen carefully to the patient, to  choose appropriate language, and to take account of the patient’s ability to communicate.” (p. 11) |
| **4. Decision-making & consent** |  |  |
| Support of patient’s decision-making capacity | I | “Alzheimer's disease should ideally be diagnosed as soon as the first symptoms appear. The diagnosis must involve a detailed cognitive assessment, preferably carried out during a specialised memory consultation.” (p. 5)  “In the case of patients with behavioural disorders, their environment must always be assessed and altered where necessary.” (p. 14) |
| Responsible surrogate decision-making (e.g. interpretation of advances directives from the perspective of the patient; seeking consent among different surrogates prior to making decisions) | N | n/a |
| Dealing with the need for advanced care planning (e.g. sensibly informing patients and relatives about the types of decision that might need to be made; informing them of tools such as advance directives*)* | E | „Assessment of the social and legal situation. […] enquiry into whether the patient has made decisions as to his/her future care and appointed someone to guard his/her interests.” (p. 19)  “The decision to move to an institution should be the outcome of a planning process that has been ongoing throughout disease progression and should be part of the care programme. Practitioners should raise this issue with patients at an early stage, when they are still sufficiently lucid to make choices.” (p. 21) |
| Adequate consideration of existing advance directives in medical decision making (e.g. guidance on how to include advance directives in medical decision making; recommendations on how to deal with conflicts between content of advance directives and present behavior and expressions in patients with severe dementia). | N | n/a |
| **5. Social and context-dependent aspects** |  |  |
| Caring for relatives (e.g. recognizing needs of relatives; preventing distress and burnout) | R  +/- | “Both family and professional carers should be given information about the disease, its treatment, and the existence of families' associations. Carers must be offered a choice of interventions: • individual or group psycho-education; • support group with other carers, tailored to meet their needs, which depend on various factors including the severity of the dementia; • telephone or Internet support; • training on dementia, services, communication, and problem-solving; • family therapy. These interventions can be offered by families' associations, local information and coordination centres, day centres, networks, etc. Specific help must be offered to carers who are experiencing psychological distress. Psychological distress and isolation affecting carers can increase the likelihood of ill treatment of patients. Day centres or respite care can give some relief to carers.” (p. 17) |
| Caring for clinical personnel and professional carers (e.g. needs of professionals; preventing distress and burn out) | R  +/- | Interventions relating to practical difficulties and a programme of education and support for family and professional carers: Both family and professional carers should be given information about the disease, its treatment, and the existence of families' associations. Carers must be offered a choice of interventions: individual or group psycho-education; support group with other carers, tailored to meet their needs, which depend on various factors including the severity of the dementia; telephone or Internet support; training on dementia, services, communication, and problem-solving; family therapy. These interventions can be offered by families' associations, local information and coordination centres, day centres, networks, etc. Specific help must be offered to carers who are experiencing psychological distress. Psychological distress and isolation affecting carers can increase the likelihood of ill treatment of patients. Day centres or respite care can give some relief to carers. |
| Adequate assessment of the patient’s potential to harm a third person (e.g. in decisions on the ability to drive) | I | “Assessment of risk situations such as the risks related to driving (In France, only the driving licence committee can decide whether an individual may continue driving and can impose restrictions), to the use of firearms, and to inappropriate money management. The doctor makes a written note of this information.” (p. 19) |
| Responsible handling of costs and allocation of limited resources (e.g. avoiding futile diagnostic and treatment) | N | n/a |
| **6. Care process & process evaluation** |  |  |
| Continuing assessment of potential benefits and harms in individual care strategies | E | “Once a patient has been told he/she has Alzheimer's disease, appropriate treatment must be arranged. Treatment should take account of the risk/benefit ratio and should be given irrespective of the patient's age and disease stage at diagnosis (except when the disease is very severe: < 2 on the MMSE scale).” (p. 11)  “Drug treatment may be considered if well-conducted non-drug measures prove inadequate. A risk/benefit analysis must always be conducted before introducing treatment. Effective analgesic treatment is a vital first step before considering administration of sedatives to patients experiencing pain. Any sedatives prescribed must be given for a short period.” (p. 14) |
| Adequate patient empowerment (e.g. environmental modification adapted to the person and the degree of impairment) | R  +/- | “In the case of patients with behavioural disorders, their environment must always be assessed and altered where necessary.” (p. 14)  “Quality of life depends on physical and mental comfort and on a suitable environment. This requires home help and an adequate staffing level in care facilities. Staff working with these patients must be trained.” (p. 16) |
| Critical self-monitoring of carers (e.g. team supervision; strategies to rethink the care process) | E | “Quality of life depends on physical and mental comfort and on a suitable environment. This requires home help and an adequate staffing level in care facilities. Staff working with these patients must be trained.”(p. 16) |
| Evaluation of abuse and neglect of patients | N | n/a |
| **7. Special situations for decision-making** |  |  |
| Sexual relationships (e.g. prevention of abuse or exploitation) | N | n/a |
| Thorough consideration of the indication for genetic testing (e.g. prevention of stigmatization) | E | “Patients with a family history of dementia suggesting autosomal dominant transmission may undergo tests for a mutation of one of the three genes currently associated with Alzheimer's disease (APP, PSEN1, PSEN2) after giving their written consent. If a mutation is present, the patient’s relatives may wish to undergo a pre-symptomatic diagnostic examination. However, they must give their written consent and must receive information and support during a multidisciplinary genetic consultation.” (p. 8) |
| Usage of GPS and other monitoring techniques (e.g. adequately balancing potential benefits, harms, and patient consent) | N | n/a |
| Particularities in drug treatment (e.g. horough consideration before prescribing) | R  +/+ | “A distinction should be made between behaviour that challenges or disturbing behavioural disorders (shouting, agitation, aggression, wandering, irritability) in patients suffering from Alzheimer's disease and related conditions, and behavioural disorders related to confusion or a  sudden event. A somatic examination is made in each case but tends to lead to over-prescription of harmful drugs, especially neuroleptics and benzodiazepines. In cases of confusion: · patients may need to be sedated so that the somatic examination can take place. Sedation must be administered at a carefully selected dose and for a short period; · an aetiological investigation must be conducted to look for: neurological comorbidity (cerebrovascular accident, subdural haematoma, non-convulsive epileptic fit, etc.) which may require a new brain scan or other additional tests, other comorbidities, especially because of their frequency and the atypical symptomatology with which they are associated: fecalith, infection (especially urinary or dental), urine retention, metabolic disorder, mycosis (especially oral) or decompensation of a chronic pathology, pain, depressive syndrome, an iatrogenic cause (for example, inappropriate psychotropic treatment, treatment that is appropriate but not well tolerated, anticholinergic treatment), a change in the patient's surroundings, or inappropriate surroundings, in particular carer exhaustion. Withdrawal, shouting and agitation may be a manifestation of pain, fecalith, an iatrogenic cause, etc. An aetiological investigation must also be performed in the event of unusual apathy or somnolence.” (p. 13-14) |
| Covert medication (e.g. balancing short-term medical benefits vs. long-term impact on patient–physician relationship) |  |  |
| Thorough consideration of the indication for brain imaging (e.g. dealing with current lack of evidence for clinical validity of brain imaging in diagnosing dementia) | N | n/a |
| Usage of physical restraints (e.g. balancing patient’s best interests with autonomy) | E | “PHYSICAL RESTRAINT CAN HASTEN THE ONSET OF AGITATION AND TRIGGER A VICIOUS CIRCLE The clinical examination to identify a trigger factor and a cause should be followed up by a work up looking out for pain or discomfort, a change in treatment, what was the onset of the behavioural disorder, and the patient's temperature. A paraclinical work up will then be carried out in the light of the clinical examination.” (p. 23) |
| Tube feeding (e.g. adequate decision making in the patient’s best interests) | N | n/a |
| Adequate end of life/palliative care (e.g. prevention of poor care or neglect) | N | n/a |
| Dealing with suicidality (e.g. appropriate estimation of quality of life in suicidal patients with dementia) | N | n/a |

| **AUSTRIA (COMPETENCE CENTER INTEGRATED CARE 2008)** | | |
| --- | --- | --- |
| DSEI | Rating | Citation example |
| **1. Diagnosis and medical indication** |  |  |
| Adequate consideration of the complexity of diagnosing dementia (e.g. unclear cut off for MCI (mild cognitive impairment)) | R +/+ | “Aufgrund der kognitiven Beeinträchtigung der Erkrankten ist neben der Eigenanamnese die Fremdanamnese von zentraler Bedeutung. Fremdanamnestische Angaben einer Persönlichkeitsveränderung können so auch z.B. Hinweis auf eine frontotemporale Demenz liefern [CMAJ 2008, S 828]. Ebenso sollten Angaben zu funktionellen Einbußen, wie das Zurechtkommen mit den Aktivitäten des täglichen Lebens, erhoben werden [CMAJ 2008, S 828]. Familien- und Sozialanamnese geben Hinweise auf (genetische) Risikofaktoren sowie aktuelle Ressourcen und Problemkonstellationen für die Krankheitsbewältigung  [DGPPN/DGN 2009].” (p. 87)  “Da es sich bei der Symptomatik von Demenzerkrankungen um einen dynamischen, progredienten Prozess handelt und viele therapeutische und präventive Ansätze gerade im Frühstadium der Erkrankung Belastung und Pflegebedürftigkeit verzögern können, ist eine frühzeitige Diagnostik von Demenzerkrankungen zu fordern. Gleichzeitig erfordert die Frühdiagnostik besondere Sorgfalt, um die existierende Möglichkeit der Stellung einer falsch-positiven Diagnose, die insbesondere früh im Krankheitsverlauf besteht, zu minimieren [DGPPN/DGN  2009].” (p. 81)  “Wenn die Patientengeschichte mit dem Ergebnis eines kognitiven Tests übereinstimmt, ist die Wahrscheinlichkeit einer Demenz sehr hoch und weitere Untersuchungen zur Abklärung sollten eingeleitet werden. Lässt zwar die Geschichte ein kognitives Impairment vermuten, das Testergebnis ist jedoch negativ, so kann das unter anderem an einer sehr leichten Form der Demenz, sehr hoher Bildung oder Ausbildung, einer vorliegenden Depression oder einer falschen Darstellung des Gesundheitszustandes durch die berichtende Person (Betroffener oder Familienmitglied) sein. Ist das Testergebnis positiv, Patientin bzw. Patient oder Familienangehörige bestreiten aber ein Nachlassen der kognitiven  Fähigkeiten, so kann das an einem akuten Verwirrtheitszustand, sehr niedriger Intelligenz und/oder Bildung oder einem Verkennen der Tatsachen durch die Familie liegen. [Knopman 1998] In schwierigen Situationen kann ein neuropsychologisches Assessment und/oder eine nochmalige Testung zu einem späteren Zeitpunkt Abhilfe schaffen [Shadlen und Larson 2009b].” (p. 90) |
| Adequate timing of diagnosis (e.g. guiding an appropriate “breaking bad news” process) | E | “Es bestehen Ängste und Vorurteile gegenüber einer Diagnose ‘Demenz’ seitens der Erkrankten und deren Angehörigen, wie auch von Seiten der Behandelnden. Gründe dafür sind die spezielle Symptomatik von Demenzerkrankungen mit dem Verlust von persönlichkeitsdefinierenden Eigenschaften, von Selbstständigkeit und Autonomie sowie die Annahme von fehlenden therapeutischen Möglichkeiten und die noch begrenzte  Wirksamkeit verfügbarer Interventionen.” (p. 81)  “Aufgrund der kognitiven Beeinträchtigung der Erkrankten ist neben der Eigenanamnese die Fremdanamnese von zentraler Bedeutung. Fremdanamnestische Angaben einer Persönlichkeitsveränderung können so auch z.B. Hinweis auf eine frontotemporale Demenz liefern [CMAJ 2008, S 828]. Ebenso sollten Angaben zu funktionellen Einbußen, wie das Zurechtkommen mit den Aktivitäten des täglichen Lebens, erhoben werden [CMAJ 2008, S 828]. Familien- und Sozialanamnese geben Hinweise auf (genetische) Risikofaktoren sowie aktuelle Ressourcen und Problemkonstellationen für die Krankheitsbewältigung [DGPPN/DGN 2009].” (p. 87) |
| Reasonableness of treatment indications (e.g. risk of overestimating benefit of pharmaceutical treatment) | I | “Einige Studien belegen den Effekt einer nicht-pharmakologischen Intervention bei Patientinnen und Patienten mit Demenz [Evidenzlevel 1a, Empfehlungsgrad B nach ÖAG 2010]. Multisensorische Stimulation erscheint effektiver als spezifisch kognitives Training. Die Kombination verschiedener Behandlungsansätze (pharmakologisch, nichtpharmakologisch) erscheint zielführend, obwohl erst wenige kontrollierte Studien verfügbar sind. Psychosoziale Aktivierung in verschiedener Form (z.B. Musik- und Kunsttherapie) oder sozialer Kontakt kann in Einzelfällen zur  Verbesserung von Verhaltensauffälligkeiten und Befinden führen [Evidenzlevel 1a, Empfehlungsgrad C nach ÖAG 2010]. Wesentlich ist die psychosoziale Unterstützung für Patientinnen und Patienten und Angehörige.” (p. 48)  “Es gibt gute Evidenz dafür, dass ein individualisiertes körperliches Trainingsprogramm einen positiven Einfluss auf die funktionelle Leistungsfähigkeit bei Personen mit leichter bis mittelschwerer Demenz hat [CMAJ 2008]. Es gibt Hinweise, dass körperliche Aktivierung zum Erhalt der Alltagsfunktionen, Beweglichkeit und Balance beiträgt. Der Einsatz kann empfohlen werden. Es existiert jedoch keine ausreichende Evidenz für die systematische Anwendung bestimmter körperlicher Aktivierungsverfahren. [Empfehlungsgrad C, Evidenzebene IIb, DGPPN/DGN  2009].” (p. 134) |
| Adequate appreciation of the patient (e.g. adequate consideration of the patient as a person; adequate consideration of existing preferences of the patient) | R +/+ | “Drei Fallstudien [Stokes 2004, Sperlinger und McAuslane 1994, Mozley et al. 1999] bieten Evidenz, dass die Einbeziehung von Betroffenen in die Evaluierung und Ausrichtung von Therapie und Pflege auch bei fortgeschrittener kognitiver Einschränkung möglich und wünschenswert ist. Alle Studien konnten nachweisen, dass Demenzerkrankte sehr wohl  noch ihre eigene Meinung und Sichtweise haben und diese auch artikulieren können. Voraussetzung dafür sind von Seiten der Betroffenen noch ein Mindestmaß an Orientierung und Sprachlichkeit, eine ruhige Umgebung und Klarheit und Aufmerksamkeit von Seiten der betreuenden oder befragenden Person. Es wird empfohlen, sich nicht auf die Beurteilung von Dritten zu verlassen, sondern die Demenzpatientinnen und -patientinnen in den Therapie- und Pflegeprozess aktiv mit einzubeziehen und ihre Meinung einzuholen. [NICE 2007] Aggressives Verhalten kann durch die Anpassung der professionellen Betreuung ebenfalls verringert werden [Stokes 2004]. Diese Maßnahmen können auch zur weiteren  Planung der Therapie und Pflege herangezogen werden. [NICE 2007].” (p. 143) |
| **2. Assessing patient decision-making capacity** |  |  |
| Dealing with varying concepts/understanding of “patient’s capacity” among carers | N | n/a |
| Adequate consideration of setting or content in assessing decision-making capacity (e.g. guidance on how to structure the setting for the capacity assessment; involvement of relatives) | I | “Hier ist auch die Einwilligungsfähigkeit der Betroffenen von Ärztin oder Arzt fest zu stellen und über die Möglichkeit einer eventuellen „Angehörigenvertretung“ aufzuklären. Eine Einschreibung kann nur bei bestehender Einwilligungsfähigkeit oder rechtskräftiger Vertretung durch Dritte durchgeführt werden. Für die Feststellung der Einwilligungsfähigkeit genügt die Einschätzung der Ärztin oder des Arztes.” (p. 53) |
| Adequate assessment of patient’s decision-making capacity (e.g. inadequate or biased assessment) | R  +/+ | “Drei Fallstudien [Stokes 2004, Sperlinger und McAuslane 1994, Mozley et al. 1999] bieten Evidenz, dass die Einbeziehung von Betroffenen in die Evaluierung und Ausrichtung von Therapie und Pflege auch bei fortgeschrittener kognitiver Einschränkung möglich und wünschenswert ist. Alle Studien konnten nachweisen, dass Demenzerkrankte sehr wohl  noch ihre eigene Meinung und Sichtweise haben und diese auch artikulieren können. Voraussetzung dafür sind von Seiten der Betroffenen noch ein Mindestmaß an Orientierung und Sprachlichkeit, eine ruhige Umgebung und Klarheit und Aufmerksamkeit von Seiten der betreuenden oder befragenden Person. Es wird empfohlen, sich nicht auf die Beurteilung von Dritten zu verlassen, sondern die Demenzpatientinnen und -patientinnen in den Therapie- und Pflegeprozess aktiv mit einzubeziehen und ihre Meinung einzuholen. [NICE 2007] Aggressives Verhalten kann durch die Anpassung der professionellen Betreuung ebenfalls verringert werden [Stokes 2004]. Diese Maßnahmen können auch zur weiteren  Planung der Therapie und Pflege herangezogen werden. [NICE 2007].” (p. 143) |
| **3. Information and disclosure** |  |  |
| Adequate consideration of the complexity of informing patients with dementia (e.g. the amount and manner of provision of information) | R  +/+ | “Drei Fallstudien [Stokes 2004, Sperlinger und McAuslane 1994, Mozley et al. 1999] bieten Evidenz, dass die Einbeziehung von Betroffenen in die Evaluierung und Ausrichtung von Therapie und Pflege auch bei fortgeschrittener kognitiver Einschränkung möglich und wünschenswert ist. Alle Studien konnten nachweisen, dass Demenzerkrankte sehr wohl  noch ihre eigene Meinung und Sichtweise haben und diese auch artikulieren können. Voraussetzung dafür sind von Seiten der Betroffenen noch ein Mindestmaß an Orientierung und Sprachlichkeit, eine ruhige Umgebung und Klarheit und Aufmerksamkeit von Seiten der betreuenden oder befragenden Person. Es wird empfohlen, sich nicht auf die Beurteilung von Dritten zu verlassen, sondern die Demenzpatientinnen und -patientinnen in den Therapie- und Pflegeprozess aktiv mit einzubeziehen und ihre Meinung einzuholen. [NICE 2007] Aggressives Verhalten kann durch die Anpassung der professionellen Betreuung ebenfalls verringert werden [Stokes 2004]. Diese Maßnahmen können auch zur weiteren  Planung der Therapie und Pflege herangezogen werden. [NICE 2007].” (p. 143) |
| Adequate involvement of relatives in the care process | R  -/+ | “Die Patientinnen und Patienten und ggf. auch ihre Angehörigen werden über die erhobenen Befunde und ihre Bedeutung im ärztlichen Gespräch in einem der persönlichen Situation des Erkrankten und der Angehörigen angemessenen Rahmen aufgeklärt, wobei sich Art und Inhalt der Aufklärung am individuellen Informationsbedarf und –wunsch sowie am Zustandsbild des Betroffenen orientieren. Die Aufklärung soll neben der Benennung der Diagnose auch Informationen zu Therapiemöglichkeiten, Verhaltensweisen im Umgang mit der Erkrankung, Hilfe- und Unterstützungsangeboten, über die Leistung der Kranken und Pflegeversicherung (in Österreich wäre dies das Pflegegeld), Betroffenen- und Angehörigenverbände, (z.B. Österreichische Alzheimerliga), und Prognose  enthalten. Dem Informationsbedürfnis der Erkrankten und der Angehörigen ist umfassend Rechnung zu tragen [Good clinical practice, Expertenkonsensus, DGPPN/DGN 2009].” (p. 37) |
| **4. Decision-making & consent** |  |  |
| Support of patient’s decision-making capacity | R  +/+ | “Drei Fallstudien [Stokes 2004, Sperlinger und McAuslane 1994, Mozley et al. 1999] bieten Evidenz, dass die Einbeziehung von Betroffenen in die Evaluierung und Ausrichtung von Therapie und Pflege auch bei fortgeschrittener kognitiver Einschränkung möglich und wünschenswert ist. Alle Studien konnten nachweisen, dass Demenzerkrankte sehr wohl  noch ihre eigene Meinung und Sichtweise haben und diese auch artikulieren können. Voraussetzung dafür sind von Seiten der Betroffenen noch ein Mindestmaß an Orientierung und Sprachlichkeit, eine ruhige Umgebung und Klarheit und Aufmerksamkeit von Seiten der betreuenden oder befragenden Person. Es wird empfohlen, sich nicht auf die Beurteilung von Dritten zu verlassen, sondern die Demenzpatientinnen und -patientinnen in den Therapie- und Pflegeprozess aktiv mit einzubeziehen und ihre Meinung einzuholen. [NICE 2007] Aggressives Verhalten kann durch die Anpassung der professionellen Betreuung ebenfalls verringert werden [Stokes 2004]. Diese Maßnahmen können auch zur weiteren  Planung der Therapie und Pflege herangezogen werden. [NICE 2007].” (p. 143) |
| Responsible surrogate decision-making (e.g. interpretation of advances directives from the perspective of the patient; seeking consent among different surrogates prior to making decisions) | R  +/- | Es wird empfohlen, sich nicht auf die Beurteilung von Dritten zu verlassen, sondern die Demenzpatientinnen und -patientinnen in den Therapie- und Pflegeprozess aktiv mit einzubeziehen und ihre Meinung einzuholen. [NICE 2007] Aggressives Verhalten kann durch die Anpassung der professionellen Betreuung ebenfalls verringert werden [Stokes 2004]. Diese Maßnahmen können auch zur weiteren Planung der Therapie und Pflege herangezogen werden. [NICE 2007].” (p. 143) |
| Dealing with the need for advanced care planning (e.g. sensibly informing patients and relatives about the types of decision that might need to be made; informing them of tools such as advance directives*)* | R  +/- | “In der primären Prävention soll vor allem das Bewusstsein für Risikofaktoren und erste Anzeichen einer Demenz bei allen Ärztinnen und Ärzten vorhanden sein, um dementsprechend frühzeitig gegensteuern zu können. Ärztinnen und Ärzte sollten sich an der öffentlichen Aufklärung über die Krankheit beteiligen und auch in Gesprächen mit ihren Patientinnen und Patienten frühzeitig darüber aufklären, wenn Risikofaktoren  bestehen. Es gibt in dieser Phase keine patientenführenden Ärztinnen und Ärzte im Sinne des Netzwerkes, vielmehr richten sich diese Aufgaben an die gesamte Ärzteschaft.”(p. 52)  “Die Netzwerkärztin oder der Netzwerkarzt ist für die Sammlung aller nötigen Informationen zur Abklärung verantwortlich und für die Koordination der einzelnen Ansprechpartner, sowie zur Informationsweitergabe und Abstimmung mit den Betroffenen und der Familie. In dieser Phase ist noch keine Case Managerin bzw. kein Case Manager vorhanden, jedoch kann die Netzwerkärztin oder der Netzwerkarzt die Netzwerkressourcen  zur Abklärung und Unterstützung nutzen. Wenn möglich und vorhanden sollten eventuelle Überweisungen und Einbindung von anderen Professionen zuerst aus den Netzwerkangeboten gedeckt werden. Während des gesamten Prozesses des Screenings und Abklärung ist oft auch eine  psychologische Begleitung der Betroffenen und der Familie notwendig. Eine wichtige Aufgabe der Netzwerkärztin oder des Netzwerkarztes ist neben der Aufklärung der Betroffenen und ihrer Familien, diese Begleitung entweder selbst durchzuführen oder sie zu organisieren.” (p. 52) |
| Adequate consideration of existing advance directives in medical decision making (e.g. guidance on how to include advance directives in medical decision making; recommendations on how to deal with conflicts between content of advance directives and present behavior and expressions in patients with severe dementia). | N | n/a |
| **5. Social and context-dependent aspects** |  |  |
| Caring for relatives (e.g. recognizing needs of relatives; preventing distress and burnout) | R  +/- | “In Österreich werden nach wie vor 80% der Demenzkranken zu Hause gepflegt, 80% der Pflege übernehmen weibliche Angehörige bzw. Ehepartner und immerhin noch 25% der Schwerstdementen befinden sich in häuslicher Pflege. Dies bedeutet auch eine enorme soziale und psychische Belastung der pflegenden Angehörigen, die zu 50% dadurch selbst  erkranken [Psota 2007]. Auch für sie bedeutet die Demenzerkrankung eine Einschränkung der Lebensqualität, verminderte Arbeitsfähigkeit und soziale Isolierung. Im Durchschnitt wenden sie 69-100 Stunden pro Woche für die Pflege ihrer erkrankten Angehörigen auf.” (p. 32)  “Wie in Punkt 3.3.1 bereits erwähnt, findet der Hauptaufwand der Versorgung in der häuslichen Umgebung statt und wird von zumeist weiblichen Angehörigen durchgeführt. Zur Unterstützung dieser pflegenden Angehörigen gibt es verschiedene Formen der ambulanten und Kurzzeitpflege, wie zum Beispiel Tageszentren, Hauskrankenpflege und  ehrenamtliche Betreuungsdienste. Die flächendeckende Verfügbarkeit ist in Österreich jedoch nicht gegeben und oft besteht für die Angehörigen auch eine Hemmschwelle, diese Angebote in Anspruch zu nehmen. Wie bei allen pflegenden Angehörigen besteht auch hier die Gefahr der Vereinsamung und einer eigenen Erkrankung aufgrund der Belastung durch  die Pflege. Entlastung können hier Selbsthilfegruppen für Angehörige schaffen, die in allen neun Bundesländern aktiv sind und zum Beispiel ‘Demenzcafés’ anbieten; der Fonds Gesundes Österreich bietet solche unter dem Namen ‚Selbsthilfe Stammtisch’ an. Diese dienen in erster Linie dazu, soziale Kontakte zu knüpfen, sich auszutauschen und sich gegenseitig zu unterstützen. Zur medizinischen und therapeutischen Versorgung der Patientinnen und Patienten gibt es zum Beispiel Gerontopsychiatrische Zentren.” (p. 33)  “Während des gesamten Prozesses des Screenings und Abklärung ist oft auch eine psychologische Begleitung der Betroffenen und der Familie notwendig. Eine wichtige Aufgabe der Netzwerkärztin oder des Netzwerkarztes ist neben der Aufklärung der Betroffenen und ihrer Familien, diese Begleitung entweder selbst durchzuführen oder sie  zu organisieren.” (p. 52)  “Während des gesamten Prozesses der Früherkennung und der Abklärung ist oft auch eine psychologische Begleitung der Betroffenen und der Familie notwendig.” (p. 105) |
| Caring for clinical personnel and professional carers (e.g. needs of professionals; preventing distress and burn out) | I | “Alle Gesundheits- und Sozialberufe, die mit der Betreuung von älteren Menschen zu tun haben, sollten über Frühwarnzeichen, Symptome und Krankheitsverläufe von Demenzerkrankungen im Rahmen ihrer spezifischen Tätigkeiten entsprechende Schulungen besuchen. [basierend auf NICE 2007, recommendation] Eine Fortbildung zu Management und Umgang mit „problematischem“ Verhalten und in der Kommunikation mit Demenzerkrankten wird empfohlen. [NICE 2007 recommendation] Evidenz-basierte Aus- und Weiterbildung mithilfe von Softwareprogrammen und Praxis-Workshops zur Unterstützung der niedergelassenen Ärztinnen und Ärzte in der Diagnose und im Management von Demenzerkrankungen sollte nach lokalen Gegebenheiten implementiert werden. Besteht die Möglichkeit eines berufsübergreifenden Angebotes, sollte dieses bevorzugt werden. [basierend auf NICE 2007, recommendation] Es wird empfohlen, dass Betreuende in Pflegeeinrichtungen in der Versorgung von Demenzkranken regelmäßig weitergebildet werden, zum Beispiel im Umgang mit Psychopharmaka oder mit aggressivem Verhalten. [basierend auf AAN 2008, guideline]” (p. 141) |
| Adequate assessment of the patient’s potential to harm a third person (e.g. in decisions on the ability to drive) | E | “Es wird empfohlen, dass Betreuende in Pflegeeinrichtungen in der Versorgung von Demenzkranken regelmäßig weitergebildet werden, zum Beispiel im Umgang mit Psychopharmaka oder mit aggressivem Verhalten. [basierend auf AAN 2008, guideline]” (p. 141) |
| Responsible handling of costs and allocation of limited resources (e.g. avoiding futile diagnostic and treatment) | I | “Daten zu Kosten von Demenz sind nur wenige vorhanden und diese können oft nur indirect deduziert werden. [Jönsson 2004] Boustani et al. (2003a) schätzen die Kosten für Demenz in den USA auf U$100 Milliarden pro Jahr. Für Europa hat Jönsson (2004) einige nationale Studien verglichen und unter anderem errechnet, dass 25% des schwedischen  Gesundheitsbudgets von der Versorgung Demenzkranker beansprucht wird und die Pflegekosten in Schweden immerhin 6% des Bruttoinlandsproduktes ausmachen. Im Ländervergleich zeigt sich deutlich der überproportionale Anstieg an Kosten für die Versorgung Schwerdemenzkranker (MMSE <15). Durch die unterschiedlichen Erhebungsverfahren, -kriterien, und –zeitpunkte sowie die Verwendung verschiedener  Referenzwährungen ist eine direkte Gegenüberstellung jedoch nicht möglich. Berechnungen aufgrund einer europäischen Metaanalyse von Jönsson und Berr (2005) ergaben, dass in Österreich jährlich etwa €1,1 Milliarden für die Versorgung Demenzkranker anfallen. Etwa drei Viertel davon machen nicht-medizinische Kosten aus. Die Kosten, die durch die medikamentöse Behandlung entstehen, betragen nur 6% der  Gesamtkosten für die Versorgung Demenzkranker. Die Patientenkosten pro Jahr würden sich demnach auf ca. €11.000 belaufen [WGKK 2009].  Den Berechnungen in der für Österreich veröffentlichten Studie von Wancata et al. (2003) wurden zwei unterschiedliche Hochrechnungen (aus Großbritannien bzw. Schweden) zugrunde gelegt. Je nachdem ergaben sich daraus Kosten für das Jahr 2000 von €0,57 Milliarden (nach englischen Zahlen) beziehungsweise €1,24 Milliarden (nach schwedischen  Zahlen). Legt man diese Zahlen auf die demographische Entwicklung um, so würden in Österreich im Jahr 2050 €1,47 Milliarden bzw. €3,2 Milliarden an Kosten für die Versorgung von Demenzpatientinnen und –patienten entstehen. Eine genaue Aufschlüsselung der Kosten nach Kostenarten für das österreichische Gesundheitssystem findet sich in Kapitel 4 des ‘Ersten Österreichischen Demenzberichtes’. [WGKK 2009]” (p. 33) |
| **6. Care process & process evaluation** |  |  |
| Continuing assessment of potential benefits and harms in individual care strategies | I | “Evidenz-basierte Aus- und Weiterbildung mithilfe von Softwareprogrammen und Praxis-Workshops zur Unterstützung der niedergelassenen Ärztinnen und Ärzte in der Diagnose und im Management von Demenzerkrankungen sollte nach lokalen Gegebenheiten implementiert werden. Besteht die Möglichkeit eines berufsübergreifenden Angebotes, sollte dieses bevorzugt werden. [basierend auf NICE 2007 recommendation]  Es wird empfohlen, dass Betreuende in Pflegeeinrichtungen in der Versorgung von Demenzkranken regelmäßig weitergebildet werden, zum Beispiel im Umgang mit Psychopharmaka oder mit aggressivem Verhalten. [basierend auf AAN 2008 guideline]” (p. 51) |
| Adequate patient empowerment (e.g. environmental modification adapted to the person and the degree of impairment) | R  -/+ | “Nach dem ‘Ersten Österreichischen Demenzbericht’ (WGKK 2009) ist die Lebensqualität der Betroffenen mit dem Fortschreiten der Demenz erheblich eingeschränkt und geht im Endstadium gänzlich verloren. Deswegen ist es umso wichtiger, durch geeignete aktivierende Therapieformen, die die Patientinnen und Patienten als auch die pflegenden  Angehörigen einbinden, den Funktionsradius so lange wie möglich zu erhalten und ein Fortschreiten der Krankheit hinaus zu zögern.” (p. 32)  “Familienähnliche Esssituationen, verbale Unterstützung und positive Verstärkung können das Essverhalten von Menschen mit Demenz verbessern und können empfohlen werden. [Empfehlungsgrad B, Evidenzebene IIb, DGPPN/DGN 2009]. Angemessene strukturierte soziale Aktivierung während des Tages kann zu einer Besserung des Tag-Nacht-Schlafverhältnisses führen und sollte eingesetzt werden. [Empfehlungsgrad B, Evidenzebene IIb, DGPPN/DGN 2009].” (p. 50) |
| Critical self-monitoring of carers (e.g. team supervision; strategies to rethink the care process) | R  -/+ | “Eine Fortbildung zu Management und Umgang mit ‘problematischem’ Verhalten und in der Kommunikation mit Demenzerkrankten wird empfohlen. [NICE 2007 recommendation]” (p. 51)  “Evidenz-basierte Aus- und Weiterbildung mithilfe von Softwareprogrammen und Praxis-Workshops zur Unterstützung der niedergelassenen Ärztinnen und Ärzte in der Diagnose und im Management von Demenzerkrankungen sollte nach lokalen Gegebenheiten implementiert werden. Besteht die Möglichkeit eines berufsübergreifenden Angebotes, sollte dieses bevorzugt werden. [basierend auf NICE 2007 recommendation]  Es wird empfohlen, dass Betreuende in Pflegeeinrichtungen in der Versorgung von Demenzkranken regelmäßig weitergebildet werden, zum Beispiel im Umgang mit Psychopharmaka oder mit aggressivem Verhalten. [basierend auf AAN 2008 guideline]” (p. 51) |
| Evaluation of abuse and neglect of patients | N | n/a |
| **7. Special situations for decision-making** |  |  |
| Sexual relationships (e.g. prevention of abuse or exploitation) | N | n/a |
| Thorough consideration of the indication for genetic testing (e.g. prevention of stigmatization) | R  +/+ | “Ein eindeutiger familiärer Hintergrund für Demenzerkrankungen sollte eine weitere Abklärung und Überweisung zu einem Spezialisten zur Beratung veranlassen. Der Verdacht auf das Vorliegen einer ‘Early-onset’-Form der Alzheimer Krankheit sollte genetisch weiter abgeklärt werden. Vor der Durchführung eines genetischen Tests ist ein genetisches Beratungsgespräch essentiell. Die Entdeckung eines genetischen Risikos für die Entwicklung einer Alzheimer Krankheit ist für die Betroffenen meist enorm belastend. Deshalb sollte ein genetischer Test niemals durchgeführt werden, bevor alle möglichen Risiken und Benefits als Konsequenz durch den Test klar überlegt und mit den Betroffenen besprochen sind [CMAJ 2008].” (p. 35) |
| Usage of GPS and other monitoring techniques (e.g. adequately balancing potential benefits, harms, and patient consent) | N | n/a |
| Particularities in drug treatment (e.g. horough consideration before prescribing) | I | “Psychotische Symptome (Halluzinationen, Wahn) bessern sich durch die Gabe von Antipsychotika bei jeder Demenzart. Antipsychotika sind daher eine unter strengen Kautelen anwendbare therapeutische Option [Evidenzlevel 1a, Empfehlungsgrad A nach ÖAG 2010]. Jede Antipsychotikatherapie bei dementen Patientinnen und Patienten muss niederdosiert starten [Empfehlungsgrad A nach ÖAG 2010], darf nur langsam erhöht  werden [Evidenzlevel 3, Empfehlungsgrad A nach ÖAG 2010] und muss initial wöchentlich, später monatlich überprüft werden [Evidenzlevel 3, Empfehlungsgrad A nach ÖAG 2010].” (p. 47) |
| Covert medication (e.g. balancing short-term medical benefits vs. long-term impact on patient–physician relationship) |  |  |
| Thorough consideration of the indication for brain imaging (e.g. dealing with current lack of evidence for clinical validity of brain imaging in diagnosing dementia) | I | “Bei vorliegendem Demenzsyndrom soll eine konventionelle cCT oder cMRT zur Differentialdiagnostik durchgeführt werden. [Empfehlungsgrad A, Leitlinienadaptation NICE 2007; DGPPN/DGN 2009]. cCT oder cMRT sollen durchgeführt werden, wenn zumindest eines der folgenden  Kriterien vorhanden ist [CMAJ 2008: Grad B Empfehlung, Level 3 Evidenz]  Alter <60 Jahren; Unerklärbare rapide (z.B. während 1-2 Monaten) Verschlechterung der kognitiven Funktion; Kurze Dauer der Demenzerkrankung (< 2 Jahren); Unerklärbare neurologische Symptome (z.B. Beginn von schweren Kopfschmerzen oder Anfällen („Seizures“); Vorliegen eines Karzinoms (besonders bei Karzinomtypen, die häufig ins  Gehirn metastasieren); Einnahme von Antikoagulantien oder bei Gerinnungsstörungen Vorkommen einer Harninkontinenz und Gangstörung im frühen Verlauf einer Demenzerkrankung (wie es häufig bei Normaldruckhydrozephalus vorkommt); Ein neu auftretendes Lokalzeichen (z.B. einer Hemiparese oder Babinsky Reflex); Unübliche oder atypische kognitive Symptome (z.B. progressive Aphasie); Gangstörungen. Es gibt ausreichend Evidenz für den Einsatz von cCT oder cMRT zum Nachweis einer zerebrovaskulären Erkrankung, die das weitere Patientinnen- und Patientenmanagement beeinflussen könnte. [CMAJ 2008: Grad B Empfehlung, Level  2 Evidenz].“ (p. 40) |
| Usage of physical restraints (e.g. balancing patient’s best interests with autonomy) | N | n/a |
| Tube feeding (e.g. adequate decision making in the patient’s best interests) | N | n/a |
| Adequate end of life/palliative care (e.g. prevention of poor care or neglect) | N | n/a |
| Dealing with suicidality (e.g. appropriate estimation of quality of life in suicidal patients with dementia) | N | n/a |

| **SWITZERLAND (EXPERT PANEL 2008)** | | |
| --- | --- | --- |
| DSEI | Rating | Citation example |
| **1. Diagnosis and medical indication** |  |  |
| Adequate consideration of the complexity of diagnosing dementia (e.g. unclear cut off for MCI (mild cognitive impairment)) | I | “Der Fremdanamnese kommt in der Demenzdiagnostik  eine besondere Bedeutung zu. Sie sollte im Einverständnis mit dem Patienten erfolgen.” (p. 145-146) |
| Adequate timing of diagnosis (e.g. guiding an appropriate “breaking bad news” process) | I | “Die erste psychosoziale Massnahme ist die frühzeitige Diagnosestellung. Sie ist eine Chance für alle Betroffenen, weil sie die Planung weiterer medizinischer Massnahmen und den rechtzeitigen Einsatz der weiteren psychosozialen Massnahmen ermöglicht und erlaubt, die Würde des an Demenz Erkrankten zu bewahren.” (p. 16)  “Der Patient ist über die Diagnose einer Demenz und die sich daraus ergebenden Konsequenzen zu orientieren. Das Einverständnis des urteils -  fähigen Patienten vorausgesetzt, ist der Einbezug einer privaten Drittperson in das Diagnosegespräch einzuplanen.” (p. 146) |
| Reasonableness of treatment indications (e.g. risk of overestimating benefit of pharmaceutical treatment) | E | “Empathie, Strukturierung des psychosozialen Umfeldes sowie adäquate Ernährung und verhaltenstherapeutische Interventionen sind wichtig.  Wird damit der gewünschte Erfolg nicht erzielt, ist zusätzlich eine Behandlung mit Psychopharmaka (ohne anticholinerge Wirkung) indiziert. Die Notwendigkeit der Anwendung muss regelmässig überprüft werden. Es liegen für die medikamentöse Therapie der BPSD nur wenige qualitativ  befriedigende Studien vor – vorwiegend solche mit Neuroleptika. Diese Pharmaka sind bei Symptomen wie Aggressionen wirksam, bei anderen, wie z.B. Wandern, Horten, Schreien, jedoch nicht. Besondere Vorsicht ist bei der Lewybody-Demenz bezüglich Therapie mit Neuroleptika geboten. Wegen erhöhter Schlaganfallrisiken unter Neuroleptika ist im Einzelfall eine Risikoabwägung vorzunehmen [34–36]. ChE-H und Memantin sind für die Behandlung diverser BPSD geeignet. Sie gehören somit zur Basistherapie der Demenz [37].” (p. 147-148) |
| Adequate appreciation of the patient (e.g. adequate consideration of the patient as a person; adequate consideration of existing preferences of the patient) | I | “Da Demenzkranke oft auch gesellschaftliche Tabus verletzen  können, besteht die Gefahr einer Isolierung der Patienten und ihrer Angehörigen.” (p. 4)  “In gleicher Weise ist die Erfassung der Urteilsfähigkeit von Patientinnen und Patienten in verschiedenen Demenzstadien schwierig. Die Schweizerische Akademie der Medizinischen Wissenschaften hat Richtlinien zur Urteilsfähigkeit erlassen [40]. Die Umsetzung dieser Grundsätze in der Praxis ist jedoch schwierig, und hier ist interdisziplinär mit Beteiligung der Betroffenen nach Lösungen zu suchen.” (p. 20) |
| **2. Assessing patient decision-making capacity** |  |  |
| Dealing with varying concepts/understanding of “patient’s capacity” among carers | N | n/a |
| Adequate consideration of setting or content in assessing decision-making capacity (e.g. guidance on how to structure the setting for the capacity assessment; involvement of relatives) | I | “In gleicher Weise ist die Erfassung der Urteilsfähigkeit von Patientinnen und Patienten in verschiedenen Demenzstadien schwierig. Die Schweizerische Akademie der Medizinischen Wissenschaften hat Richtlinien zur Urteilsfähigkeit erlassen [40]. Die Umsetzung dieser Grundsätze in der Praxis ist jedoch schwierig, und hier ist interdisziplinär mit Beteiligung der Betroffenen nach Lösungen zu suchen.” (p. 20) |
| Adequate assessment of patient’s decision-making capacity (e.g. inadequate or biased assessment) | I | “In gleicher Weise ist die Erfassung der Urteilsfähigkeit von Patientinnen und Patienten in verschiedenen Demenzstadien schwierig. Die Schweizerische Akademie der Medizinischen Wissenschaften hat Richtlinien zur Urteilsfähigkeit erlassen [40]. Die Umsetzung dieser Grundsätze in der Praxis ist jedoch schwierig, und hier ist interdisziplinär mit Beteiligung der Betroffenen nach Lösungen zu suchen.” (p. 20) |
| **3. Information and disclosure** |  |  |
| Adequate consideration of the complexity of informing patients with dementia (e.g. the amount and manner of provision of information) | N | n/a |
| Adequate involvement of relatives in the care process | I | “In gleicher Weise ist die Erfassung der Urteilsfähigkeit von Patientinnen und Patienten in verschiedenen Demenzstadien schwierig. Die Schweizerische Akademie der Medizinischen Wissenschaften hat Richtlinien zur Urteilsfähigkeit erlassen [40]. Die Umsetzung dieser Grundsätze in der Praxis ist jedoch schwierig, und hier ist interdisziplinär mit Beteiligung der Betroffenen nach Lösungen zu suchen.” (p. 20) |
| **4. Decision-making & consent** |  |  |
| Support of patient’s decision-making capacity | I | “Spezifische Ziele der psychosozialen Massnahmen sind zum einen die Optimierung von Wohlbefinden und Alltagsfunktionen der Patienten über die Verbesserung ihrer kognitiven, funktionellen und psychopathologischen Störungen. Ebenso wichtig ist das Empowerment der Betreuungspersonen in Bezug auf den Umgang mit der Belastung und den  Erhalt der eigenen (auch psychischen) Gesundheit.” (p. 16-17) |
| Responsible surrogate decision-making (e.g. interpretation of advances directives from the perspective of the patient; seeking consent among different surrogates prior to making decisions) | N | n/a |
| Dealing with the need for advanced care planning (e.g. sensibly informing patients and relatives about the types of decision that might need to be made; informing them of tools such as advance directives*)* | R  +/- | “Bei einem unauffälligen Befund kann der Patient beruhigt werden. Beim Befund einer Demenz im Frühstadium erhält der Patient die Möglichkeit, seine Therapiewünsche mit den Angehörigen zu besprechen, eine Patientenverfügung zu formulieren und Vollmachten zu regeln. Gleichzeitig können sich Angehörige frühzeitig mit dem Krankheitsbild der Demenz auseinandersetzen und, wenn nötig, Hilfeleistungen von aussen in Anspruch nehmen.” (p. 4) |
| Adequate consideration of existing advance directives in medical decision making (e.g. guidance on how to include advance directives in medical decision making; recommendations on how to deal with conflicts between content of advance directives and present behavior and expressions in patients with severe dementia). | N | n/a |
| **5. Social and context-dependent aspects** |  |  |
| Caring for relatives (e.g. recognizing needs of relatives; preventing distress and burnout) | R  +/- | “Ebenso wichtig ist das Empowerment der Betreuungspersonen in  Bezug auf den Umgang mit der Belastung und den Erhalt der eigenen (auch psychischen) Gesundheit.” (p. 148)  “Alle an Demenz erkrankten Menschen leben in einem Umfeld. Deshalb müssen psychosoziale Massnahmen dem Kranken und allen Betreuenden dienen. (p. 4)-17)000000000000000000000000000000000000000000000000000000000000000000000000000000000000000000000000000000000000000000000Mögliche Ziele sind: Wissen und Fertigkeiten gewinnen, Wohlbefinden erhalten, Vertrauen in die eigenen Fähigkeiten entwickeln, mit Belastungen umgehen und schwierige Entscheidungen fällen können, Gesundheitsstörungen vorbeugen und behandeln, die Zukunft planen können, Scham- und Schuldgefühle vermindern, Trauer verarbeiten usw.” (p. 17) |
| Caring for clinical personnel and professional carers (e.g. needs of professionals; preventing distress and burn out) | R  +/- | “Alle an Demenz erkrankten Menschen leben in einem Umfeld. Deshalb müssen psychosoziale Massnahmen dem Kranken und allen Betreuenden dienen. (p. 4)-17)000000000000000000000000000000000000000000000000000000000000000000000000000000000000000000000000000000000000000000000Mögliche Ziele sind: Wissen und Fertigkeiten gewinnen, Wohlbefinden erhalten, Vertrauen in die eigenen Fähigkeiten entwickeln, mit Belastungen umgehen und schwierige Entscheidungen fällen können, Gesundheitsstörungen vorbeugen und behandeln, die Zukunft planen können, Scham- und Schuldgefühle vermindern, Trauer verarbeiten usw.” (p. 17) |
| Adequate assessment of the patient’s potential to harm a third person (e.g. in decisions on the ability to drive) | E | “Die Fahrtauglichkeit ist bei mittelschwerer und schwerer Demenz in der Regel nicht mehr gegeben. Bei leichter Demenz ist sie meist eingeschränkt  oder nicht mehr gegeben, wenn eine der folgenden Bedingungen erfüllt ist: – fremdanamnestisch auffälliges Fahrverhalten oder Unfälle (auch Bagatellunfälle); – Störungen der (geteilten) Aufmerksamkeit,  Störung der Raumverarbeitung; – Tagesschläfrigkeit. Wenn die Fahrtauglichkeit bezweifelt warden muss und der Patient nicht freiwillig auf den Fahrausweis verzichten möchte, sollte eine Meldung an das kantonale Strassenverkehrsamt bzw. die Motorfahrzeugkontrolle gemacht werden. Dort findet eine expertenbegleitete Kontrollfahrt statt,  deren Ergebnis dann endgültig ist.” (p. 145) |
| Responsible handling of costs and allocation of limited resources (e.g. avoiding futile diagnostic and treatment) | I | “Häufig bestehen Probleme bei der Finanzierung der nötigen Massnahmen.” (p. 144) |
| **6. Care process & process evaluation** |  |  |
| Continuing assessment of potential benefits and harms in individual care strategies | N | n/a |
| Adequate patient empowerment (e.g. environmental modification adapted to the person and the degree of impairment) | N | n/a |
| Critical self-monitoring of carers (e.g. team supervision; strategies to rethink the care process) | E | “Die psychosozialen Massnahmen sind stets die tragenden Säulen des Behandlungskonzepts. Ziel dieser Massnahmen ist die Optimierung der  Lebensqualität der Patienten und ihrer Angehörigen/Betreuenden. Neue Analysen zeigen, dass diese Interventionen bedeutsame Effektstärken aufweisen (siehe [39]). Konkrete Vorgehensweisen für Patienten und Betreuende sind in jedem Einzelfall den Bedürfnissen anzupassen und regelmässig auf ihre Angemessenheit zu überprüfen. Wichtige ratgebende und unterstützende Institutionen sind Haus- oder Spezialärzte, Memory  Clinics und andere spezialisierte Institutionen, ambulante Pflegezentren, Selbsthilfegruppen, alle Angebote der Schweizerischen Alzheimervereinigung und, soweit vorhanden, lokale spezialisierte Institutionen mit temporären Entlastungsangeboten für die Betreuer oder für die Dauerbeherbergung von an Demenz erkrankten Menschen. Angehörige mit Erfahrung in der Pflege und Betreuung von Demenzkranken können  kompetente Berater für andere betroffene Familien und Institutionen sein. Spezifische Ziele der psychosozialen Massnahmen sind zum einen die Optimierung von Wohlbefinden und Alltagsfunktionen der Patienten über die Verbesserung ihrer kognitiven, funktionellen und psychopathologischen Störungen.” (p. 148) |
| Evaluation of abuse and neglect of patients | N | n/a |
| **7. Special situations for decision-making** |  |  |
| Sexual relationships (e.g. prevention of abuse or exploitation) | N | n/a |
| Thorough consideration of the indication for genetic testing (e.g. prevention of stigmatization) | I | “Gezielte genetische Analysen bei präsenilen familiären Demenzformen (z.B. Chorea Huntington, Mutationen auf den Chromosomen 1, 14 oder 21) können diagnostisch hilfreich sein. Eine entsprechende genetische Beratung ist aber vor und nach der Analyse unabdingbar.” (p. 146) |
| Usage of GPS and other monitoring techniques (e.g. adequately balancing potential benefits, harms, and patient consent) | N | n/a |
| Particularities in drug treatment (e.g. horough consideration before prescribing) | N | n/a |
| Covert medication (e.g. balancing short-term medical benefits vs. long-term impact on patient–physician relationship) |  |  |
| Thorough consideration of the indication for brain imaging (e.g. dealing with current lack of evidence for clinical validity of brain imaging in diagnosing dementia) | I | “Die Durchführung einer MRI-Untersuchung oder eines CT (meist ohne Kontrast) wird als routinemässige initiale Evaluation bei allen Demenzpatienten empfohlen. Die CT-Untersuchung dient vornehmlich dem Ausschluss sekundärer Demenzformen (Subduralhämatom, Tumor, Blutung, vaskuläre Läsion); die MRI-Untersuchung kann auch Fragen spezifischer Atrophiemuster (z.B. Hippocampus) beantworten und ist  insbesondere bei jüngeren Patienten und atypischer Symptomalogie dringend zu empfehlen. Funktionelle bildgebende Verfahren wie PET und SPECT sind speziellen Fragestellungen vorbehalten. Sie können für die Früherkennung sowie Differentialdiagnose degenerativer Demenzen (Abgrenzung zwischen frontotemporaler Demenz, Demenz mit Lewy-Körperchen und Alzheimer Krankheit) sehr hilfreich sein.” (p. 146) |
| Usage of physical restraints (e.g. balancing patient’s best interests with autonomy) | N | n/a |
| Tube feeding (e.g. adequate decision making in the patient’s best interests) | N | n/a |
| Adequate end of life/palliative care (e.g. prevention of poor care or neglect) | N | n/a |
| Dealing with suicidality (e.g. appropriate estimation of quality of life in suicidal patients with dementia) | N | n/a |
