# Supplementary material for: Inclusion of Ethical Issues in Dementia Guidelines: A Thematic Text Analysis
Source: PLoS Med. 2013 Aug 13;10(8):e1001498. doi: 10.1371/journal.pmed.1001498 (PMC3742442; doi:10.1371/journal.pmed.1001498)
Supplement: Table S2 — Text examples regarding the DSEI “Adequate involvement of relatives in the care process” for a selection of five CPGs. (DOC) [file pmed.1001498.s002.doc]

Table S2: Text examples regarding the DSEI “Adequate involvement of relatives in the care process” for a selection of 5 CPGs

| **Guideline** | **Text examples** |
| --- | --- |
| SIGN [32] | “There is a consensus that both people with dementia and their carers are entitled to receive relevant information. […] Patients and carers should be offered information tailored to the patient's perceived needs. … Formal permission to disclose the diagnosis to carers should be sought.” |
| NICE [26] | “Health and social care professionals should inform people with dementia and their carers about advocacy services and voluntary support, and should encourage their use. If required, such services should be available for both people with dementia and their carers independently of each other. […] |
| Malay [30] | “Following disclosure to patient, support should be provided to patients and caregivers. […] Both […] are entitled to receive relevant information regarding dementia, treatment, available support services, as well as legal, financial and benefits advice.” |
| APA [35] | “An important task of the psychiatrist who cares for an individual with dementia is providing or coordinating the education of the patient and family regarding the illness and its natural history. Often the ﬁrst step is to communicate and explain the diagnosis of dementia, including the speciﬁc dementia etiology, if known. Terms should be clariﬁed at the outset to facilitate communication. […] it may be helpful to seek the family’s input regarding the nature and timing of any discussion with the patient” |
| CMA [25] | “Although each case should be considered individually, in general the diagnosis of dementia should be disclosed to the patient and family. This process should include a discussion of prognosis, diagnostic uncertainty, advance planning, driving issues, treatment options, support groups, and future plans. […]” |
